# Supplementary material for: β-Carotene alleviates substrate inhibition caused by asymmetric cooperativity
Source: Nat Commun. 2025 Mar 29;16:3065. doi: 10.1038/s41467-025-58259-7 (PMC11954892; doi:10.1038/s41467-025-58259-7)

Supplementary information for

β-Carotene alleviates substrate inhibition caused by asymmetric cooperativity

Jieren Liao, Umar F. Shahul Hameed, Timothy D. Hoffmann, Elisabeth Kurze, Guangxin Sun, Wieland Steinchen, Alessandro Nicoli, Antonella Di Pizio, Christina Kuttler, Chuankui Song, Dragana A. M. Catici, Farhah Assaad-Gerbert, Thomas Hoffmann, Stefan T. Arold, Wilfried G. Schwab*

Corresponding author: wilfried.schwab@tum.de

The PDF file includes:

Supplementary Notes 1-14
Supplementary Figures 1 to 40
Supplementary Table
Supplementary References

Supplementary Notes

Supplementary Note 1.

Molecular dynamics (MD) simulations illustrates the flexibility of Trp350.

To investigate the flexibility of Trp350, we ran Molecular Dynamics (MD) simulations of complex V, as it represented the most resolved structure (Supplementary Data 5), with and without ligands, for an aggregated time of 12 µs. The backbone of the protein proved to be stable through all the MD simulations (Supplementary Figure 22), but we noticed a high flexibility of the α-helix involving residues 42-61 and the loop between residues 305 and 328 (Supplementary Figure 23). On the other hand, when we examined the flexibility of the ligands, we observed that scopoletin in its pocket was highly dynamic in the absence of UDPG (Supplementary Figure 24). This could explain why scopoletin is rotated by 180° in complex VIII. The simulated systems provide insights into the conformational landscape of the protein, starting from the unproductive complex V. We used the MD trajectories to monitor the motion of Trp350 and see how it is affected by the presence of the ligands. Trp350 exhibits considerable flexibility after removal of the ligands, moving through all of the rotameric states depicted by the structures and even more (Supplementary Figure 25 and 26). In the simulation without ligands, starting from complex V, we obtained six defined chi1 ($\chi1$)/chi2 ($\chi2$) combinations, which are illustrated by the point clouds in Supplementary Figure 26 and have been described previously[1]. In the simulations of the apo protein, Trp350 can explore states observed in complexes I, II, III, IV, V, VIII, and IX. In the simulations of the unproductive complex V with UDPG, Trp350 has a reduced dynamics and does not adopt any of the conformations observed in complexes I and II. Angle chi2 (χ_2), which determines the side chain orientation, is stabilized. Therefore, the populations of Trp350 conformers sampled by MD simulations illustrates how the ligands restrict the rotational mobility of Trp350. The strongest effect is observed by UDPG, supporting the hypothesis that the order of ligand binding affects the protein conformations. The simulated protein with scopoletin shows a similar torsion angle pattern compared to the apo enzyme, as scopoletin is not tightly bound and can be released from the protein (Supplementary Figure 26C,E). The Trp350 rotamers of the active complexes (VI_A, VI_D and VI_F) are not observed when scopoletin is in the protein (Supplementary Figure 26C,E), but when UDPG is liganded to the protein, these rotamers appear (Supplementary Figure 26B,D). Therefore, the dynamics and conformational changes of the protein are different when scopoletin or UDPG is bound.

Supplementary Note 2.

Hydrogen/deuterium exchange mass spectrometry (HDX) shows that scopoletin and (apo)carotenoids bind at similar positions in the protein.

Although β-carotene and retinol are not visible in *Nb*UGT72AY1 complexes by X-ray crystallography, the conformers formed in the presence of (apo)carotenoids (II, VI-IX) are structurally distinct from their (apo)carotenoid-free enzyme (I, III-V). To experimentally confirm the results of the *in-silico* molecular docking study (Supplementary Figures 14 to17), HDX-MS was performed to verify the interaction of β-carotene, β-apo-8‘-carotenal, and retinol with the protein (Supplementary Data 6)[2]. After addition of the (apo)carotenoids to the protein, a reduced HDX was observed in all cases (Supplementary Figure 27,28). The effect was most pronounced around Phe87, an amino acid in the binding pocket of scopoletin[3,4], indicating binding in the active site. In particular, upon addition of β-carotene, the greatly reduced isotope exchange of other amino acids of the protein (Supplementary Figure 29A) indicates additional nonspecific binding sites as

predicted by docking (Supplementary Figure 14). These findings were also confirmed with the help of Woods plots (Supplementary Figures 30-37)[5]. After addition of the effectors, a reduced HDX around Phe87 was noted. This result proves the interaction of the (apo)carotenoids with the enzyme, resulting in the observed conformational change (Fig. 2). Thus, as competitive inhibitors, the effectors remove water molecules from the active site and protect UDP-glucose from unwanted hydrolysis (Fig. 1D). Similarly, C13-norisoprenoids such as ionones and ionols likely inhibit the UDP-glucose glucohydrolase activity of *Nb*UGT72AY1[4].

Supplementary Note 3.
Melting curves indicate different thermostability of the protein in combination with substrates and ligands.

Differential scanning fluorimetry (DSF) was used to measure the thermal denaturation temperatures and thus the stability of *Nb*UGT72AY1 as well as the stability in combination with 100 µM substrates and the effector retinol (Supplementary Figure 38). Retinol (100 µM) was tested due to its favourable water-solubility. The apo-enzyme showed a melting temperature (Tm) of 55 ± 0.4 °C, while the addition of acceptor and donor substrate increased Tm by 2 and 4.5 °C, respectively (Supplementary Figure 38B). Both together even increased the Tm by 10 °C. In contrast, the effector retinol reduced the Tm of the protein by 2 °C and that of the enzyme occupied by acceptor and donor substrate by as much as 4 °C. This result confirms the functional interaction of the apocarotenoid with the enzyme leading to a loss of stability, which can be explained by higher flexibility of the protein structure. This hypothesis is supported by the non-resolvable closing loops in the crystal structures obtained in the presence of retinol. However, the loss of enzyme stability has the beneficial effect of reducing SI by the acceptor substrate scopoletin. This finding is consistent with results showing that mutations in AK of E. coli can lead to a stability-dependent increase in SI by the acceptor substrate AMP, thereby impairing the overall activity of the enzyme at high stability[6]. Our results go beyond this and show that effectors can reduce and almost prevent SI of UGT, concomitant with loss of stability.

Supplementary Note 4.
The kinetic equation was revised to explain SI in *Nb*UGT72AY1.

A second acceptor-binding site was recently postulated as the cause of SI in *Nb*UGT72AY1[3]. The SI enzyme kinetics could be explained with a mathematical relation derived from the Michaelis-Menten equation, Hill equation, and the equation for two binding sites (Supplementary Note 7). This relationship had been developed for the intensively studied aspartate transcarbamylase (ATC)[7,8], and also described the SI of an anthocyanidin *Fv*UGT1 from *Fragaria vesca*[9], among others[10]. However, X-ray crystal analysis performed with *Nb*UGT72AY1 failed to confirm a second binding site for the acceptor substrate, and ITC measurements corroborated a 1:1 stoichiometry of protein and scopoletin (Fig. 2; Supplementary Figure 20). Therefore, based on the hypothesis derived from the structures, equations were developed to explain the kinetic data. Equations based only on the Michaelis-Menten relation cannot describe the sigmoid curve shape of the SI of *Nb*UGT72AY1 (Supplementary Notes 8,9). However, by using the Hill equation, it was possible to derive a mathematical solution that is a simplified version of the LiCata and Allewell equation[7] and closely reproduces the kinetic data of *Nb*UGT72AY1. The Hill coefficient is a key parameter of protein-ligand binding that measures the degree of cooperativity between subunits of a protein complex capable of binding a ligand in several of its subunits. It is also a measure of the cooperative interaction between binding sites within a protein and is generally used to estimate the number of ligands required to bind to a protein to effect a structural or catalytic

change. The Hill equation also comes into effect when the protein is assumed to occur in different conformations and the ligands bind only to the accessible binding site, causing a population shift that translates into an enhancement of the activity[11]. In this case, the Hill coefficient is a measure of the protein population shift (Supplementary Note 10). We therefore derived an equation for the enzyme rate based on the Hill relationship for a bi-substrate enzyme (Supplementary Note 11), and then extended the equation for proteins that bind acceptor and donor substrates to two different conformers, while one conformer does not catalyse the reaction (Supplementary Note 12; equ. 1). Equation 1 (Supplementary Note 13) could be simplified for variable concentrations of acceptor (equ. 2) or donor substrate (equ. 3). The resulting relationship (equ. 4) valid for both substrates differs from the equation (equ. 5) proposed by[7] in the deletion of the mathematical terms for a second acceptor substrate. Equation 4 (Supplementary Figure 45) was used to successfully approximate all the kinetic data of *Nb*UGT72AY1 and its mutant proteins.

Supplementary Note 5.
Analysis of enzyme mutants identifies amino acids that are crucial for SI.

To confirm the importance of structural changes during catalysis and to support the postulated hypothesis, a series of single, double, and chimera mutants were generated, and their enzyme properties investigated (Supplementary Data 4). In a preliminary study, derived from HDX data, F87 and two overlapping sequence segments V154-D209 (chimera) and V154-D192 (chimera A) were already identified as important factors of SI in *Nb*UGT72AY1[3]. Calculation of the kinetic parameters using simplified equation 4 (Supplementary Figure 45) revealed that SI was attenuated in the three mutants because of the greatly increased K_D values for scopoletin (39 ± 4, 362 ± 56 and 331 ± 49 µM, respectively versus 5-20 ± 3 µM in the various wild type controls). Isothermal titration calorimetry (ITC) measurements confirmed a K_D of 4 ± 1 µM for scopoletin with ΔG of -7.3 ± 1.0 kcal/mol (Supplementary Figure 20). The F87I mutant also showed increased v_{max} (1474 ± 50 versus 218-871 ± 57 nmol/min/mg for the various controls). To identify the amino acids of the chimera A sequence that contribute to SI, all residues that were different in a related non-SI UGT sequence from *Solanum tuberosum* were mutated to the amino acids occurring in the non-SI UGT. Eleven mutants were generated, with Y163H, V184M, and to a lesser extent L187M_L188M reducing the SI of the enzyme from *N. benthamiana*, but none of the mutants showed as strong an effect as chimera A (Supplementary Data 4). V184 is directed toward the scopoletin, and Y163H and L187_L188 are amino acids of the hydrophobic acceptor-binding pocket (Fig. 5). Furthermore, T145L, another residue in the scopoletin binding pocket, W350A, the first amino acid of the PSPG box (UDPG binding pocket) and Y317F, the amino acid of the closing loop projecting into the active site were mutated (Fig. 5). All three mutants significantly reduced the SI of *Nb*UGT72AY1 by increasing K_D levels to 45 ± 3, 54 ± 9, and 24 ± 2 µM, respectively, compared with wild type enzyme values, which ranged from 5-20 ± 3 µM (Supplementary Data 4). The results prove that the strong affinity of scopoletin for the enzyme is the driving force behind SI. If the affinity is attenuated, by mutation of amino acids of the scopoletin binding site, e.g. F87, T145, V184 and Y317, the substrate inhibits its conversion only at higher concentrations. This may even be accompanied by an overall increased reaction rate as shown by the F87I and T145L mutants. The same effect is achieved by adding the (apo)carotenoids (Supplementary Data 4). The effectors β-carotene, lycopene, β-apo-8'-carotenal and retinol increased the K_D values for scopoletin from 6-15 µM to 10-46 µM and v_{max} from 276-944 to 399-1832 nmol/min/mg. In this context, the effect of the W350A mutant is striking. Although W350 does not participate in the binding of scopoletin, this amino acid has a decisive influence on SI, as

already concluded from the crystal structures (Fig. 2) and confirmed by the kinetic investigation of the mutant (Supplementary Data 4).

Supplementary Note 6.
*Nb*UGT72AY1 shows hysteresis.

Hysteresis describes the slow response of an enzyme to changes in ligand concentration[12]. The response can be attributed to slow transitions in the enzyme structure associated with substrate binding or product release, and therefore hysteresis produces a time-dependent alteration in enzyme activity. Similarly, the reaction rates of *Nb*UGT72AY1 were different in assays in which the enzyme was pre-incubated with β-carotene and in assays in which β-carotene was added after pre-incubation with scopoletin (Supplementary Figure 39A). A more detailed time-dependent analysis of β-carotene pre-incubation and subsequent calculation of kinetic parameters using equation 2 showed that the change in K_S is completed at about 300 sec, while v_{max} shows a minimum at this time point (Supplementary Figure 39B). Thus, β-carotene induces a slow conformational change in the minute range. In contrast, a 30 sec pre-treatment with scopoletin already reduced v_{max} and K_S to a stable value (Supplementary Figure 39C).

Supplementary Note 7.
Kinetic equations

Equations, models and corresponding curves are shown. Michaelis Menten (v rate of formation of product, v_{max} maximum velocity, S substrate concentration, K_m Michaelis constant), Hill (n Hill coefficient), substrate inhibition (v_i rate in the presence of inhibition, K_i inhibition constant), substrate inhibition allostery assumed (x second Hill coefficient). E_0 total enzyme, k_{cat} catalytic rate constant, act activity.

model name	equation	model	curves
Michaelis-Menten (hyperbola)	$v = \frac{v_{max} * \frac{[S]}{K_m}}{1 + \frac{[S]}{K_m}}$	$E + S \underset{}{\overset{K_m}{\rightleftharpoons}} ES \xrightarrow{k_{cat}} E + P$	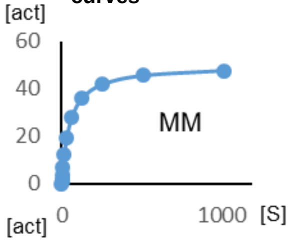
Hill (multiple binding; cooperativity)	$v = \frac{v_{max} * \frac{[S]^n}{K_m^n}}{1 + \frac{[S]^n}{K_m^n}}$	$E + nS \overset{K_m}{\rightleftharpoons} ES_n \xrightarrow{k_{cat}} E + P_n$	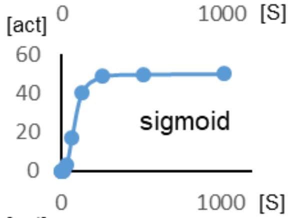
Substrate inhibition (SI) (two-binding sites)	$v = \frac{v_{max} * \frac{[S]}{K_m} + v_i * \frac{[S] * [S]}{K_m * K_i}}{1 + \frac{[S]}{K_m} + \frac{[S] * [S]}{K_m * K_i}}$	$E + S \overset{K_m}{\rightleftharpoons} ES \xrightarrow{k_{cat}} E + P$; $ES + S \overset{K_i}{\rightleftharpoons} ESS \xrightarrow{k_{cat(i)}} ES + P$; $v_{max} = k_{cat} * [E_0]$	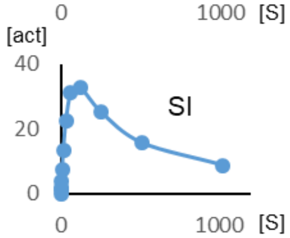
Substrate inhibition allostery assumed (partially competitive inhibition)	$v = \frac{v_{max} * \frac{[S]^n}{K_m^n} + v_i * \frac{[S]^n * [S]^x}{K_m^n * K_i^x}}{1 + \frac{[S]^n}{K_m^n} + \frac{[S]^n * [S]^x}{K_m^n * K_i^x}}$	$E + nS \overset{K_m}{\rightleftharpoons} ES_n \xrightarrow{k_{cat}} E + P$; $ES_n + xS \overset{K_i}{\rightleftharpoons} ES_{n+x} \xrightarrow{k_{cat(i)}} E + P$; $v_{max} = k_{cat} * [E_0]$	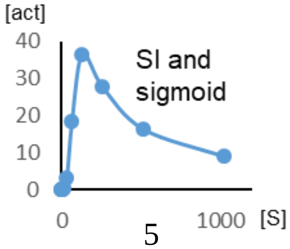

Supplementary Note 8.

Calculation of the reaction velocity equation for a random-ordered bi-substrate enzyme.

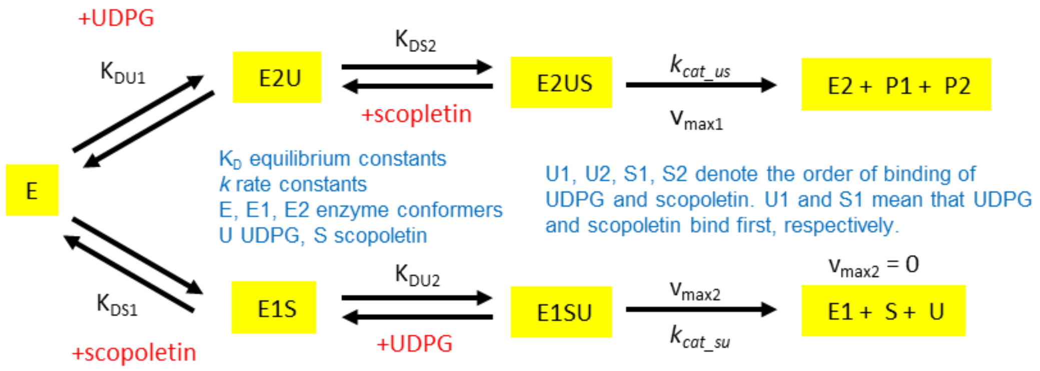

Based on the model shown and the following rules, a rate equation for glycosylation was derived.
The denominator of the velocity equation is the sum of all specific terms (protein complexes).
The specific term for free enzyme is always 1. E.g. $1 + [U]/K_{DU1} + [S]/K_{DS1} + \ldots$
The numerator is the sum of specific terms for catalytically active species, each weighted by their respective v_{max} values.

$$v = \frac{v_{max1} * \frac{[S]*[U]}{K_{DU1}*K_{DS2}} + v_{max2} * \frac{[S]*[U]}{K_{DS1}*K_{DU2}}}{1+\frac{[U]}{K_{DU1}} + \frac{[S]*[U]}{K_{DU1}*K_{DS2}} + \frac{[S]}{K_{DS1}} + \frac{[S]*[U]}{K_{DS1}*K_{DU2}}} \quad if\ v_{max2} = 0 \quad = \frac{v_{max1} * \frac{[S]*[U]}{K_{DU1}*K_{DS2}}}{1+\frac{[U]}{K_{DU1}} + \frac{[S]*[U]}{K_{DU1}*K_{DS2}} + \frac{[S]}{K_{DS1}} + \frac{[S]*[U]}{K_{DS1}*K_{DU2}}}$$

X-ray crystal structures indicate one binding site for UDPG; it follows: $K_{DU1} = K_{DU2} = K_{DU}$.
Scopoletin is non-competitive inhibitor of UDPG and thus reduces v_{max1} by $(1/(1 + [S]/K_{DS1}))$ and competitive inhibitor in the second conformer (E1) and increases K_{DS2} by $(1 + [S]/K_{DS1})$, (refer to Supplementary Note 9).

$$v = \frac{v_{max1} * \frac{1}{(1+\frac{[S]}{K_{DS1}})} * \frac{[S]*[U]}{K_{DU}*K_{DS2}*(1+\frac{[S]}{K_{DS1}})}}{1+\frac{[U]}{K_{DU}} + \frac{[S]*[U]}{K_{DU}*K_{DS2}*(1+\frac{[S]}{K_{DS1}})} + \frac{[S]}{K_{DS1}} + \frac{[S]*[U]}{K_{DS1}*K_{DU}}}$$

general equation for random-ordered bi-substrate enzyme

Resolution of the equation as a **function of UDPG concentration** by reducing the mathematical fraction by /S and $*K_{DU}*K_{DS2}$ yields

$$v = \frac{v_{max1} * \frac{1}{(1+\frac{[S]}{K_{DS1}})^2} * [U]}{K_{DU} * \left(\frac{K_{DS2}}{[S]} + \frac{K_{DS2}}{K_{DS1}}\right) + [U] * \left(\frac{K_{DS2}}{[S]} + \frac{1}{(1+\frac{[S]}{K_{DS1}})} + \frac{K_{DS2}}{K_{DS1}}\right)}$$

X-ray crystal structures indicate one binding site for scopoletin; it follows: $K_{DS1} = K_{DS2} = K$.

$$v = \frac{v_{max1} * \frac{1}{(1+\frac{[S]}{K})^2} * [U]}{K_{DU} * \left(\frac{K}{[S]} + 1\right) + [U] * \left(\frac{K}{[S]} + \frac{1}{(1+\frac{[S]}{K})} + 1\right)}$$

Since the red coloured terms are constants, the equation represents a Michaelis-Menten relation

Resolution of the general equation (see above) as a **function of scopoletin concentration** by reducing the mathematical fraction by /U and $*K_{DU}*K_{DS2}$ yields

$$v = \frac{v_{max1} * \frac{1}{(1+\frac{[S]}{K_{DS1}})^2} * [S]}{K_{DS2} * \left(1 + \frac{K_{DU}}{[U]}\right) + [S] * \left(\frac{K_{DS2}*K_{DU}}{[U]*K_{DS1}} + \frac{1}{(1+\frac{[S]}{K_{DS1}})} + \frac{K_{DS2}}{K_{DS1}}\right)}$$

X-ray crystal structures indicate one binding site for scopoletin; it follows: $K_{DS1} = K_{DS2} = K$.

$$v = \frac{v_{max1} * \frac{1}{(1+\frac{[S]}{K})^2} * [S]}{K * \left(1 + \frac{K_{DU}}{[U]}\right) + [S] * \left(\frac{K_{DU}}{[U]} + \frac{1}{(1+\frac{[S]}{K})} + 1\right)}$$

The corresponding graph shows a typical SI curve but not the S-shaped curve of a sigmoid function

Supplementary Note 9.
Comparison of enzyme models.

(**A**) Classic model for competitive inhibition. (**B**) Classic model for non-competitive inhibition. (**C**) Model derived in this study. Scopoletin acts as a competitive inhibitor for the acceptor because it can bind to the second protein conformer. (**D**) Scopoletin acts as a non-competitive inhibitor for the donor because it can bind to the enzyme and enzyme-substrate (UDPG) complex. (**E**) Initial velocity equations for competitive and non-competitive inhibition models. *S* substrate, I inhibitor, E enzyme, E1 and E2 enzyme conformer 1 and 2, respectively, P product, v_{max} maximum velocity, K_m Michaelis constant, K_i inhibition constant.

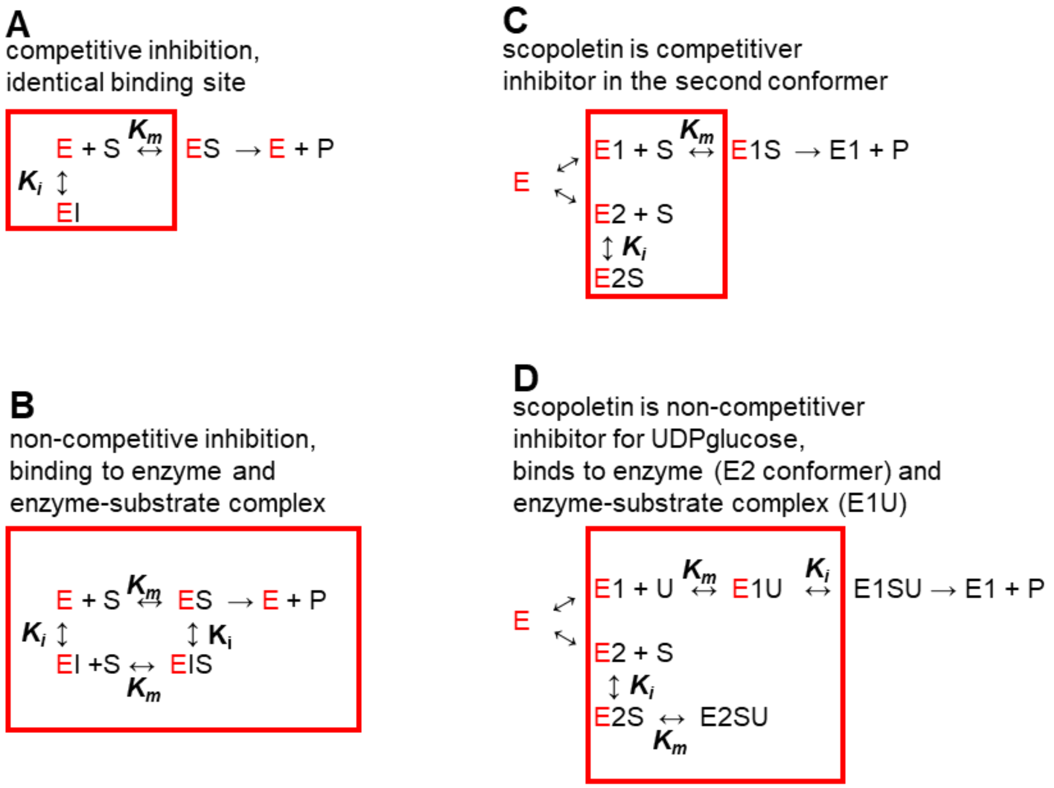

E

mechanism	initial velocity equation
competitive	$\frac{v_{max} * [S]}{K_m \left(1 + \frac{[I]}{K_i}\right) + [S]}$
non-competitive	$\frac{v_{max} * [S]}{\left(1 + \frac{[I]}{K_i}\right)(K_m + [S])}$

Supplementary Note 10.
Comparison of derivations of the Hill equation.

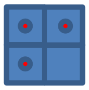

Classic (subunits)

A protein *P* is considered with *n* binding sites for ligands *L*.

$$P + nL \underset{k_d}{\overset{k_a}{\rightleftharpoons}} PL_n$$

The binding of *L* to *P* can be represented by the chemical equilibrium expression where k_a (rate of association of the *PL* complex) and k_d (rate of dissociation of the *PL* complex) are the reaction rate constants. The apparent dissociation constant K_d, is given by

$$K_d = \frac{k_d}{k_a} = \frac{[P] * [L]^n}{[PL_n]}$$

The ratio of the concentration of occupied protein to total protein concentration is given by

$$\theta = \frac{occupied\ protein}{total\ protein} = \frac{[PL_n]}{[P] + [PL_n]}$$

By using the expression obtained earlier for K_d, we can replace $[PL_n]$ with $[P] \bullet [L]^n/K_d$ to yield a simplified expression for θ.

$$\theta = \frac{\frac{[P]*[L]^n}{K_d}}{[P]+\frac{[P]*[L]^n}{K_d}} = \frac{[P]*[L]^n}{[P]*K_d + [P]*[L]^n} = \frac{[L]^n}{K_d+[L]^n}$$

Assuming that the protein was initially completely unbound at a concentration $[P_0]$, then at any time, $[P] + [PL_n] = [P_0]$ and $\theta = [PL_n]/[P_0]$.
The Hill equation is written as

$$[PL_n] = [P_0] * \frac{[L]^n}{K_d+[L]^n}$$

The equations assume that the protein has n sites to which ligands can bind. In practice, the Hill coefficient n rarely provides an accurate value of the number of ligand binding sites

New view (conformers)

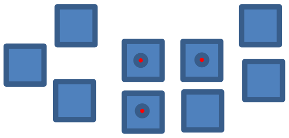

A protein *P* exists as different conformers in dynamic equilibrium, *n* ligands can bind to *n* conformers if the only binding site is accessible

$$P + nL \underset{k_d}{\overset{k_a}{\rightleftharpoons}} nPL$$

The binding of *L* to *P* can be represented by the chemical equilibrium expression where k_a (rate of association of the *PL* complex) and k_d (rate of dissociation of the PL complex) are the reaction rate constants. The apparent dissociation constant K_d, is given by

$$K_d = \frac{[P] * [L]^n}{[PL]^n}$$

The ratio of the concentration of occupied protein to total protein concentration is given by

$$\theta = \frac{occupied\ conformers}{total\ protein} = \frac{[PL]^n}{[P] + [PL]^n}$$

By using the expression obtained earlier for K_d, we can replace $[PL]^n$ with $[P] \bullet [L]^n/K_d$ to yield a simplified expression for θ.

$$\theta = \frac{\frac{[P]*[L]^n}{K_d}}{[P]+\frac{[P]*[L]^n}{K_d}} = \frac{[P]*[L]^n}{[P]*K_d + [P]*[L]^n} = \frac{[L]^n}{K_d+[L]^n}$$

Assuming that the protein was initially completely unbound at a concentration $[P_0]$, then at any time, $[P] + [PL]^n = [P_0]$ and $\theta = [PL]^n/[P_0]$.
The Hill equation is written as

$$[PL]^n = [P_0] * \frac{[L]^n}{K_d+[L]^n}$$

Here, *n* denotes the number of ligands that bind to protein conformers with the dissociation constant K_d. The Hill coefficient *n* establishes a relationship between the conformers that can bind the ligand with a given K_d value and the conformers that are unable to bind the ligand due to spatial constraints.

Supplementary Note 11.
Derivation of a Hill equation for a bi-substrate enzyme.

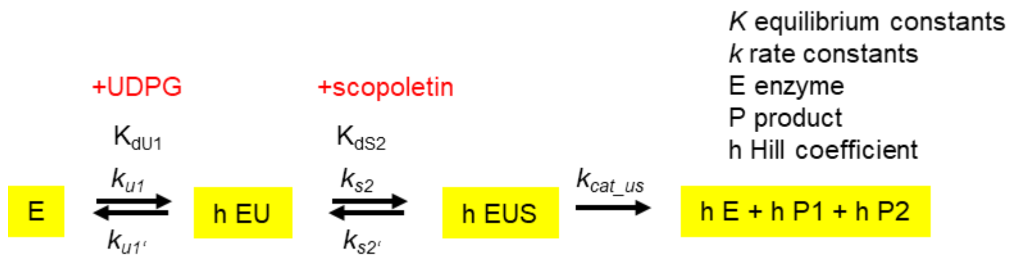

$$K_{dU1} = \frac{[E] * [U]^h}{[EU]^h} = K_{DU1}{}^h \qquad K_{dS2} = \frac{[EU]^h * [S]^h}{[EUS]^h} = K_{DS2}{}^h$$

NOTE: K_d is equal to the ratio of the dissociation rate of the ligand-receptor complex to its association rate $K_{dU1} = k_{u1'}/k_{u1}$. K_d is the equilibrium constant for dissociation.

K_D is defined so that $(K_D)^h = K_d = k_d/k_a$ this is also known as the microscopic dissociation constant and is the ligand concentration occupying half of the binding sites.

$[EU]^h$ reduced $\quad * \frac{[U]^h}{K_{dU1}}$

$$\theta = \frac{occupied\ with\ both\ substrates}{total\ protein} = \frac{[EUS]^h}{[E]+[EU]^h+[EUS]^h} = \frac{\frac{[EU]^h*[S]^h}{K_{dS2}}}{K_{dU1}*\frac{[EU]^h}{[U]^h} + [EU]^h + \frac{[EU]^h*[S]^h}{K_{dS2}}} = \frac{\frac{[S]^h}{K_{dS2}}}{\frac{K_{dU1}}{[U]^h} + 1 + \frac{[S]^h}{K_{dS2}}} = \frac{\frac{[S]^h}{K_{dS2}} * \frac{[U]^h}{K_{dU1}}}{1 + \frac{[U]^h}{K_{dU1}} + \frac{[S]^h*[U]^h}{K_{dS2}*K_{dU1}}} = \frac{v}{v_{max}}$$

Supplementary Note 12.

Derivation of a Hill equation for a bi-substrate enzyme with two conformers, with one conformer inactive.

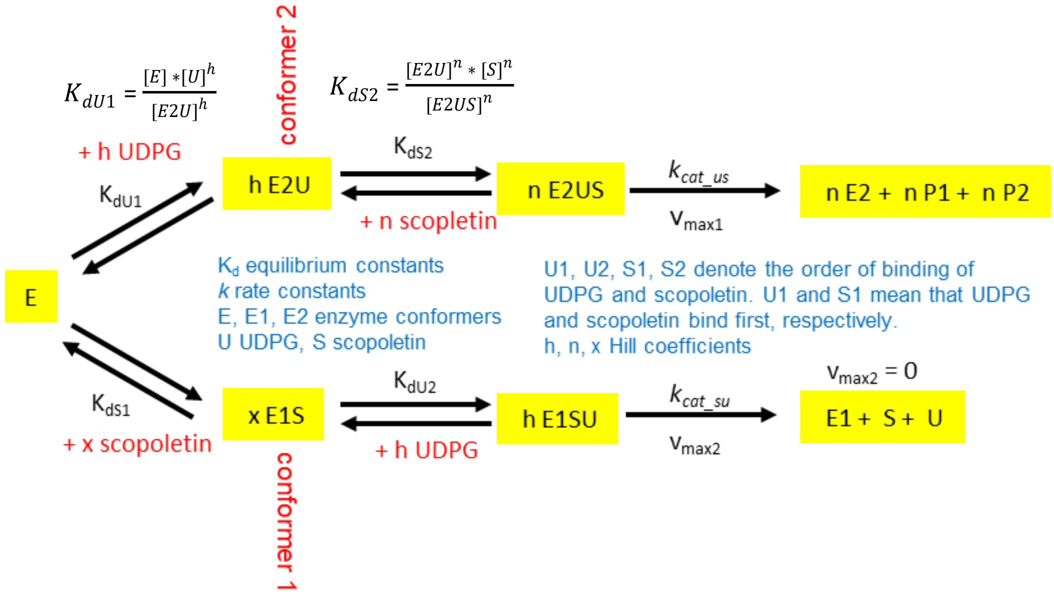

Different Hill coefficients were assumed for UDPG (h) and scopoletin, and further, because of scopoletin substrate inhibition, two Hill coefficients (n, x) were assumed for scopoletin, one for each conformer.

$$\theta = \frac{occupied\ \mathrm{R\ (active)}\ state}{total\ enzyme} = \frac{v}{v_{max}} = \frac{[E2US]^n}{[E]+[E2U]^h+[E2US]^n+[E1S]^x+[E1SU]^h} = \frac{\frac{[E]*[U]^h*[S]^n}{K_{dU1}*K_{dS2}}}{[E]+\frac{[E]*[U]^h}{K_{dU1}}+\frac{[E]*[U]^h*[S]^n}{K_{dU1}*K_{dS2}}+\frac{[E]*[S]^x}{K_{dS1}}+\frac{[E]*[S]^x*[U]^h}{K_{dS1}*K_{dU2}}}$$

$$\frac{v}{v_{max}} = \frac{\frac{[U]^h * [S]^n}{K_{dU1} * K_{dS2}}}{1 + \frac{[U]^h}{K_{dU1}} + \frac{[U]^h * [S]^n}{K_{dU1} * K_{dS2}} + \frac{[S]^x}{K_{dS1}} + \frac{[S]^x * [U]^h}{K_{dS1} * K_{dU2}}}$$

K_D is defined so that $(K_D)^{\text{Hill coefficient}} = K_d$ this is also known as the microscopic dissociation constant and is the ligand concentration occupying half of the binding sites.

$$\frac{v}{v_{max}} = \frac{\frac{[U]^h * [S]^n}{K_{DU1}{}^h * K_{DS2}{}^n}}{1 + \frac{[U]^h}{K_{DU1}{}^h} + \frac{[U]^h * [S]^n}{K_{DU1}{}^h * K_{DS2}{}^n} + \frac{[S]^x}{K_{DS1}{}^x} + \frac{[S]^x * [U]^h}{K_{DS1}{}^x * K_{DU2}{}^h}} \qquad \text{Equ. 1}$$

Supplementary Note 13.
Derivation of the enzyme rate equation for a monomeric bi-substrate protein that occurs in at least two conformations.

Both conformers can bind the substrate, but one is inactive.
Hill equation for a bisubstrate enzyme with two conformers, with one conformer inactive.

$$\frac{v}{v_{max}} = \frac{\frac{[U]^h * [S]^n}{K_{DU1}{}^h * K_{DS2}{}^n}}{1 + \frac{[U]^h}{K_{DU1}{}^h} + \frac{[U]^h * [S]^n}{K_{DU1}{}^h * K_{DS2}{}^n} + \frac{[S]^x}{K_{DS1}{}^x} + \frac{[S]^x * [U]^h}{K_{DS1}{}^x * K_{DU2}{}^h}} \qquad \text{Equ. 1}$$

Derivation such that **scopoletin is variable** by reducing $K_{DU1}{}^h$ / $[U]^h$ and assuming $K_{DU1} = K_{DU2} = K_U$ and $K_{DS1} = K_{DS2} = K_S$ gives

$$\frac{v}{v_{max}} = \frac{\frac{[S]^n}{K_S{}^n}}{1+\frac{K_U{}^h}{[U]^h}+\frac{[S]^n}{K_S{}^n}+\frac{[S]^x}{K_S{}^x}*\left(1+\frac{K_U{}^h}{[U]^h}\right)}$$

If [U] approaches infinity $K_U{}^h$ / $[U]^h$ becomes 0 and the equation simplifies to

$$\frac{v}{v_{max}} = \frac{\frac{[S]^n}{K_S{}^n}}{1+\frac{[S]^n}{K_S{}^n} + \frac{[S]^x}{K_S{}^x}} \qquad \text{Equ. 2}$$

Derivation such that **UDPG is variable** by reducing $K_{DS2}{}^n$ / $[S]^n$ and assuming $K_{DU1} = K_{DU2} = K_U$ and $K_{DS1} = K_{DS2} = K_S$ gives

$$\frac{v}{v_{max}} = \frac{\frac{[U]^h}{K_U{}^h}}{\left(\frac{K_S{}^n}{[S]^n}+K_S{}^{n-x}*[S]^{x-n}\right)+\frac{[U]^h}{K_U{}^h}*\left(1+\frac{K_S{}^n}{[S]^n}+K_S{}^{n-x}*[S]^{x-n}\right)}$$

If $\left(\frac{K_S{}^n}{[S]^n} + K_S{}^{n-x} * [S]^{x-n}\right) = 1$, the equation simplifies to

$$\frac{v}{v_{max}} = \frac{\frac{[U]^h}{K_U{}^h}}{1 + \frac{[U]^h}{K_U{}^h} * 2} = \frac{\frac{[U]^h}{K_U{}^h}}{1 + \frac{[U]^h}{K_U{}^h} + \frac{[U]^h}{K_U{}^h}} \qquad \text{Equ. 3}$$

and thus ressembles equ. 2 obtained for variable scopoletin
Consequently, we can formulate a general equation for SI in *Nb*UGT72AY1.

$$v = \frac{v_{max} * \frac{[S]^n}{K_D{}^n}}{1 + \frac{[S]^n}{K_D{}^n} + \frac{[S]^x}{K_D{}^x}} \qquad \text{Equ. 4}$$

Equation derived in this study. SI in a monomer with only one binding site but two conformers.

$$v = \frac{v_{max} * \frac{[S]^n}{K_m^n} + v_i * \frac{[S]^n * [S]^x}{K_m^n * K_i^x}}{1 + \frac{[S]^n}{K_m^n} + \frac{[S]^n * [S]^x}{K_m^n * K_i^x}} \qquad \text{Equ. 5}$$

LiCata and Allewell, 1997
SI based on the binding of two acceptors[7]

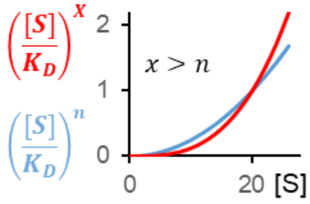

$\left(\frac{[S]}{K_D}\right)^x$ exceeds $\left(\frac{[S]}{K_D}\right)^n$ if $[S] = K_D$ and $\left(\frac{[S]}{K_D}\right) = 1$

then *S* starts to inhibit the reaction

Supplementary Note 14.
Derivation of the relationship between the intrinsic allosteric efficacy factor α and the Hill coefficient.

The allosteric descriptor α was first introduced by Tsai and Nussinov 2014 [15].

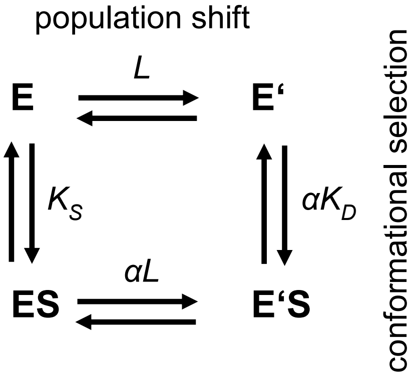

$$L = \frac{[E']}{[E]} \qquad K_D = \frac{[ES]}{[E][S]}$$

$$\alpha L = \frac{[E'S]}{[ES]} \qquad \alpha K_D = \frac{[E'S]}{[E'][S]}$$

$$response\ (allosteric) = \frac{\alpha L}{1+\alpha L}$$

$$\frac{v}{v_{max}} = \frac{\frac{[S]^n}{K_S^n}}{1 + \frac{[S]^n}{K_S^n}} \quad \text{Hill}$$

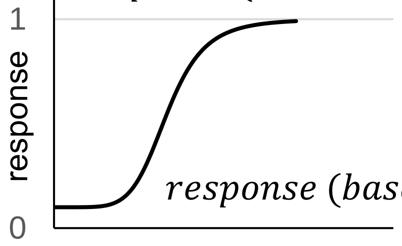

$$response\ (basal) = \frac{L}{1+L}$$

$$\frac{v}{v_{max}} = \frac{\frac{[S]}{K_S}}{1 + \frac{[S]}{K_S}} \quad \text{Michaelis-Menten}$$

$$aL = \frac{[S]^n}{K_S^n} \qquad a = \frac{[S]^{n-1}}{K_S^{n-1}}$$

Supplementary Figures

A

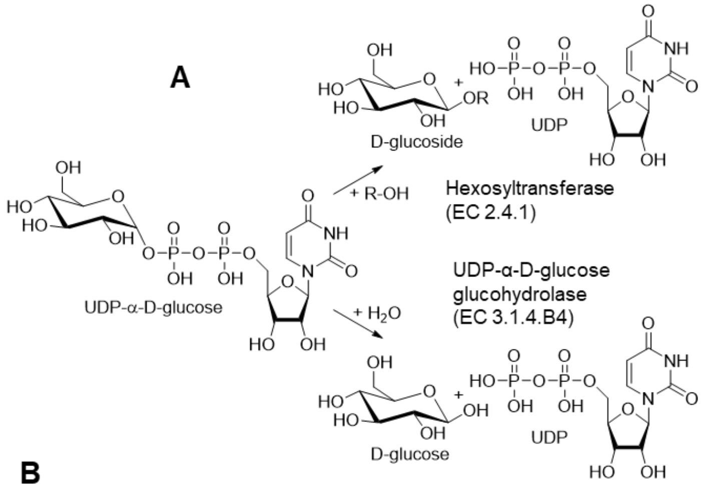

B

substrates	UGT 71AJ1	UGT 72AX1	UGT 72AY1	UGT 72B35	UGT 72B34	UGT 73A24	UGT 73A25	UGT 85A73
Retinol	0	0	0	0	0	0	0	0
Farnesol	0	0	21	0	0	100	37	5
α-Ionol	2	0	51	2	2	60	100	16
β-Ionol	2	3	49	2	1	76	100	24
2-Phenylethanol	0	0	56	0	0	59	26	100
Furanmethanethiol	0	0	0	0	0	0	0	0
Carvacrol	31	33	50	48	58	100	42	2
Geraniol	1	2	23	1	11	81	47	100
Hydroquinone	13	22	55	81	100	0	0	0
Kaempferol	87	18	92	100	71	24	37	5
Scopoletin	8	32	72	61	79	100	82	4
Mandelonitrile	0	4	0	0	0	0	0	100
3-cis-Hexenol	0	17	41	0	35	23	14	100
Perillyl alcohol	0	0	100	3	16	82	74	90
1-Octen-3-ol	0	25	55	2	13	56	33	100
Benzyl alcohol	10	31	32	44	100	0	0	83
Mandelic acid	0	0	0	0	0	0	0	0
α-Bisabolol	0	0	0	0	0	0	0	0
Lavandulol	2	19	33	1	16	100	73	30
Borneol	0	0	0	0	0	79	75	100
Carveol	14	29	46	5	26	25	11	100
Fenchyl alcohol	0	0	0	0	0	0	0	0
4-Carvomenthenol	0	0	0	0	0	0	0	100
Tyrosol	0	0	100	10	54	33	16	16
Myrtenol	1	5	29	3	32	37	58	100
3-Oxo-α-ionol	0	0	100	0	0	46	27	0
Converted substrates	11	13	18	14	15	17	17	19

C D

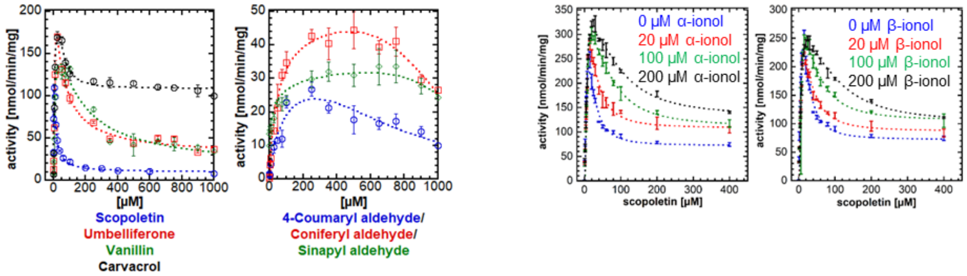

Supplementary Figure 1.

Biochemical characterization of *Nb*UGT72AY1 in previous studies. (**A**) *Nb*UGT72AY1 exhibits hexosyltransferase and UDP-α-D-glucose glucohydrolase activity. Adapted from [4] (**B**) Substrate promiscuity of *Nb*UGT72AY1. Adapted from [13] (**C**) *Nb*UGT72AY1 shows strong substrate inhibition (SI) with hydroxycoumarins and related substrates but reduced SI with monolignols. Adapted from [3] (**D**) SI by scopoletin is mitigated by apocarotenoids such as α- and β-ionol. Adapted from [4].

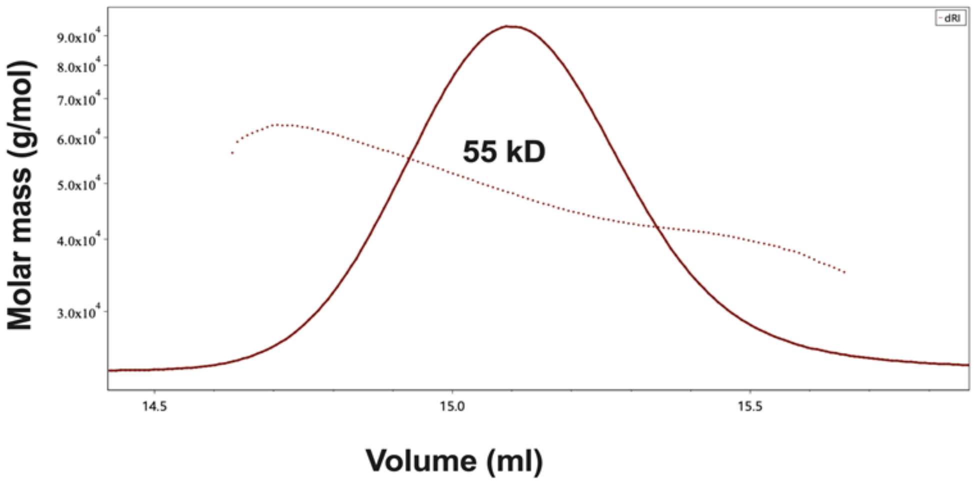

Supplementary Figure 2.

Molecular weight determination of *Nb*UGT72AY1 using SEC-MALS. The protein is a monomer. For details, see Methods.

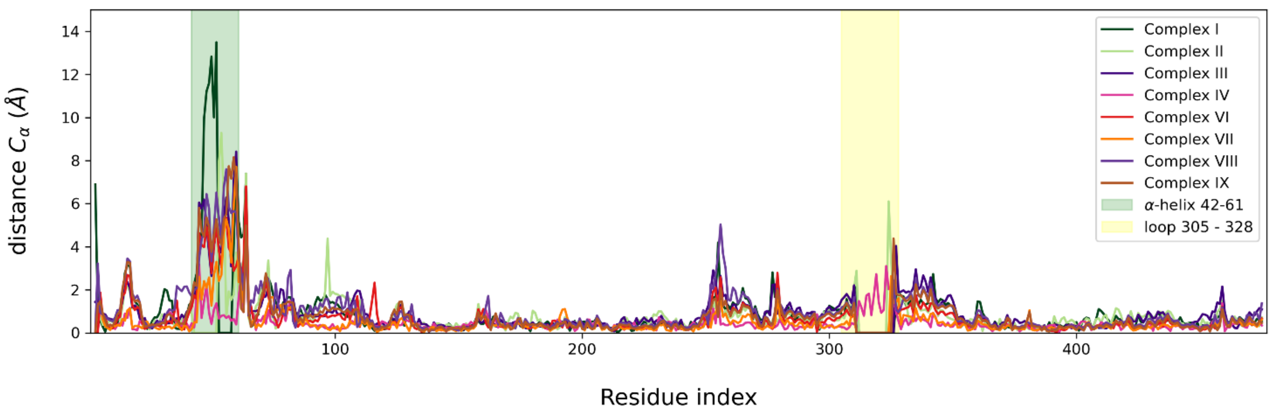

Supplementary Figure 3.

Distance between Cα of all structures vs. complex V (*Nb*UGT72AY1•scopoletin/UDP2FG: 9J9K). The region corresponding to the α-helix of residues 42-61 (highlighted in green) shows the highest diversity among the structures. The loop between residues 305 and 328 (highlighted in yellow) is solved only in complexes IV and V (structure used as reference).

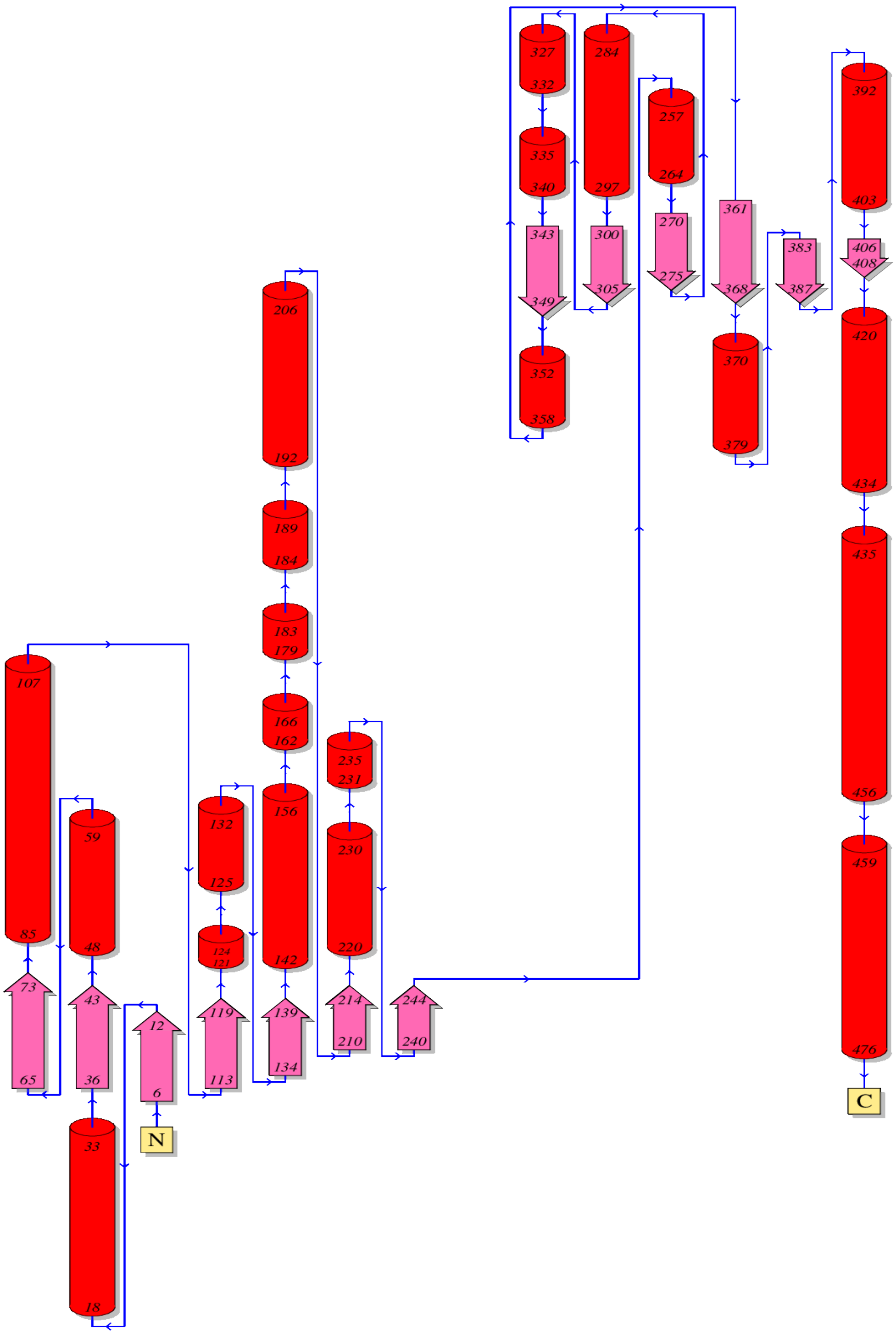

Supplementary Figure 4.

Topology of *Nb*UGT72AY1. Complex **V:** NbUGT72AY1•scopoletin/UDP2FG: 9J9K. Helices and sheets are shown as cylinders and arrows, respectively. N- and C-terminal ends are labeled.

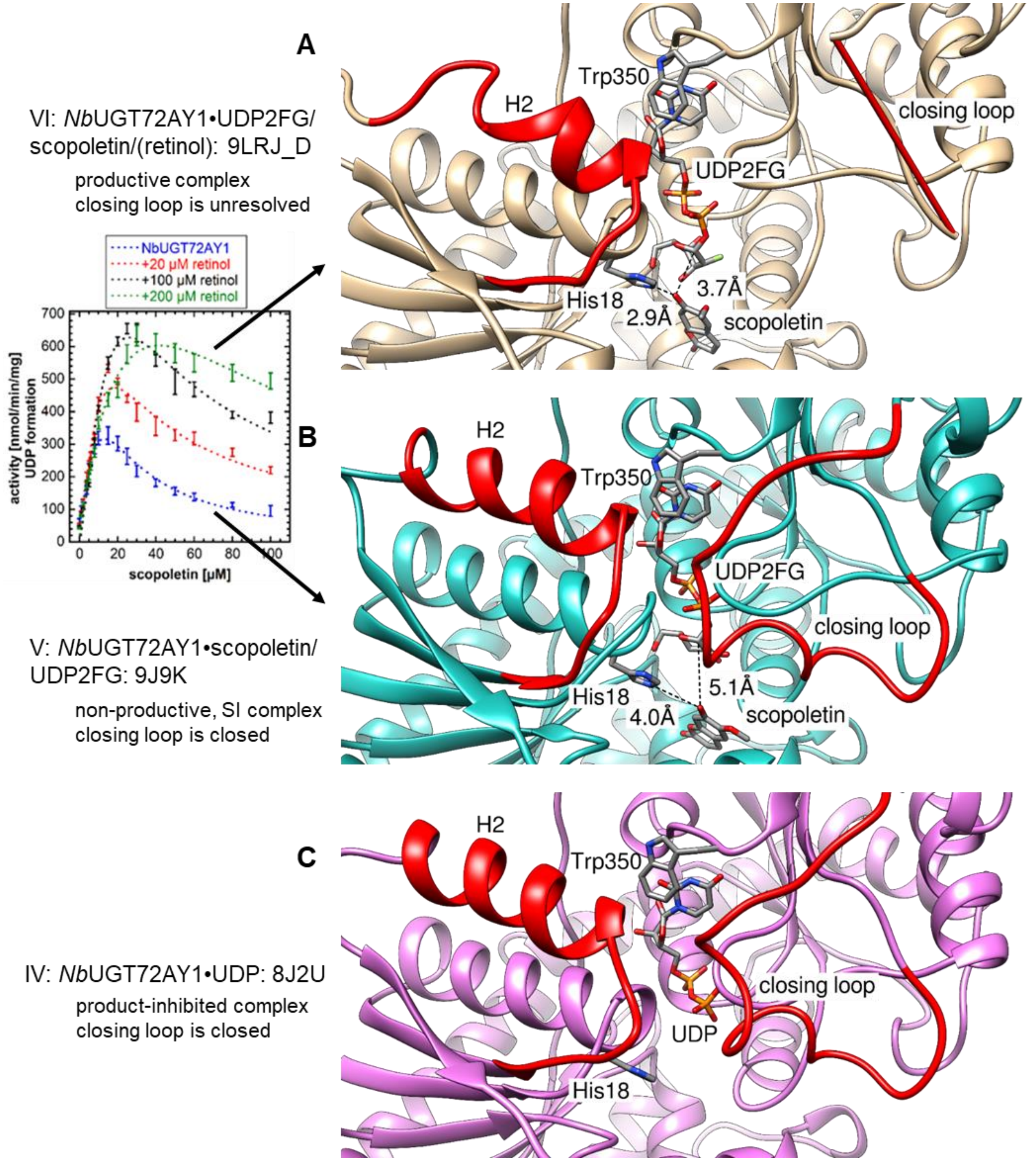

Supplementary Figure 5.

Close up of the structural differences in the productive, substrate-inhibited (SI), and product-inhibited *Nb*UGT72AY1 complexes. (**A**) The catalytically active complex **VI**: 9LRJ_D was obtained in the presence of retinol. (**B**) The SI complex is formed at high concentrations of the acceptor substrate. (**C**) The product UDP released from UDPG inhibits the enzyme. H2 denotes helix 2 (loop/helix transition).

A

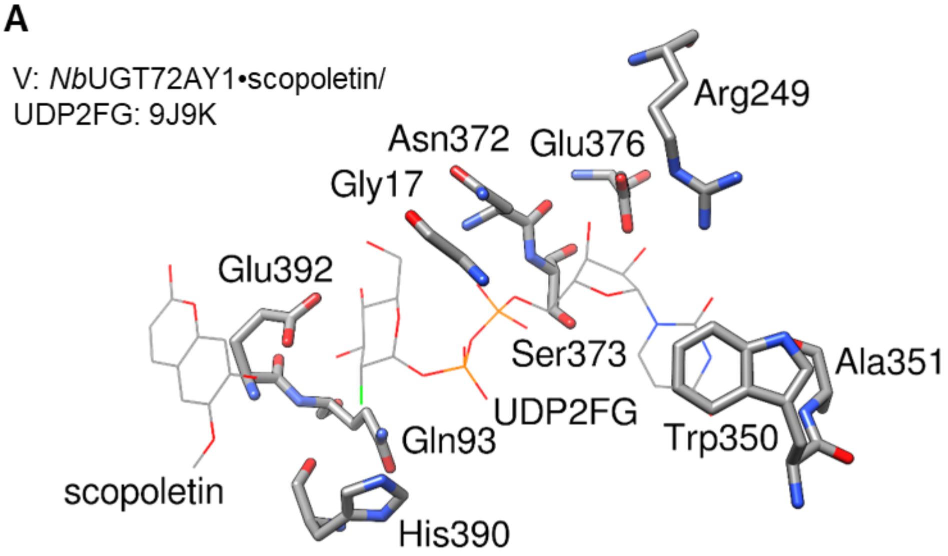

B

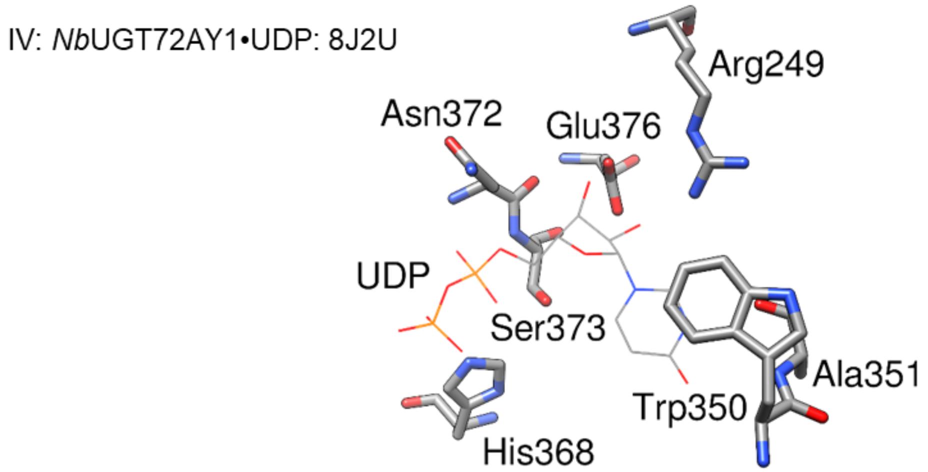

Supplementary Figure 6.

Close up of the structural similarity in the substrate-inhibited and product-inhibited complex. (**A**) Non-productive SI complex V: *Nb*UGT72AY1•scopoletin/UDP2FG: 9J9K. (**B**) Non-productive product-inhibited complex IV: *Nb*UGT72AY1•UDP: 8J2U. Amino acids in 3 Å spacing of UDP2FG and UDP are shown. In both cases, Asn372, Ser373, Glu376, Arg249, Ala351, and Trp350 frame the donor-binding site (< 3Å). UDP2FG, scopoletin, and UDP are displayed in the wire representation.

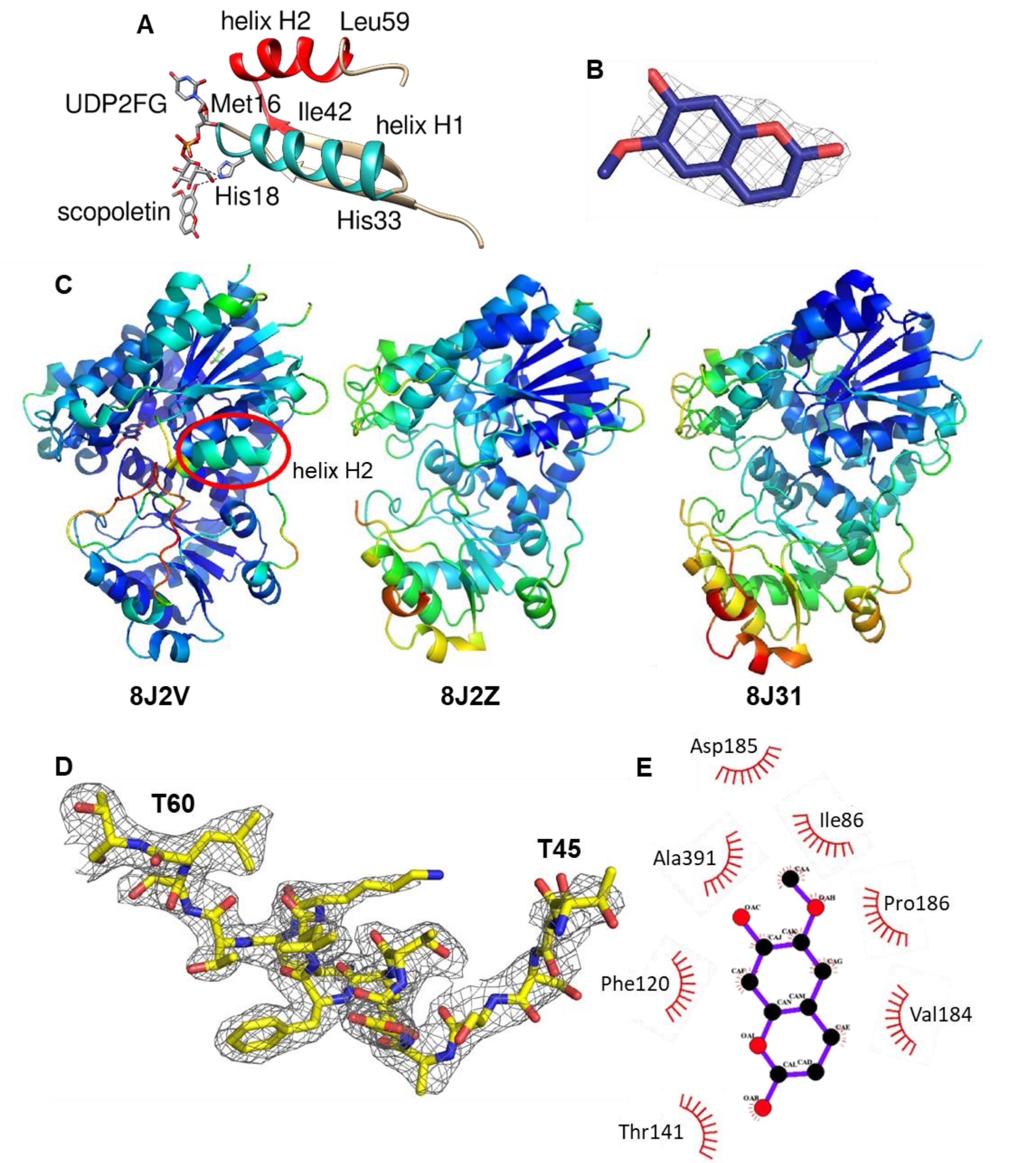

Supplementary Figure 7.
Analysis of protein structures. (A) Arrangement of α-helices H1 (Met16-His33) and H2 (Ile42-Leu59) in complex V: NbUGT72AY1•scopoletin/UDP2FG: 9J9K**.** (**B**) $2F_o$-F_c omit map at 1 **σ** cutoff for the scopoletin (blue stick) bound in the structure 8J2V. (**C)** Cartoon representation based on the b-factor of the residues in scopoletin bound structure (8J2V), apo structure (8J2Z), and β-carotene soaked structure (8J31) (**D**) $2F_o$-F_c omit map at 1 **σ** cutoff for the residues 45-60 (yellow sticks) in the scopoletin bound structure 8J2V. (**E**) Ligplot for Scopoletin (sticks) with the neighboring protein residues.

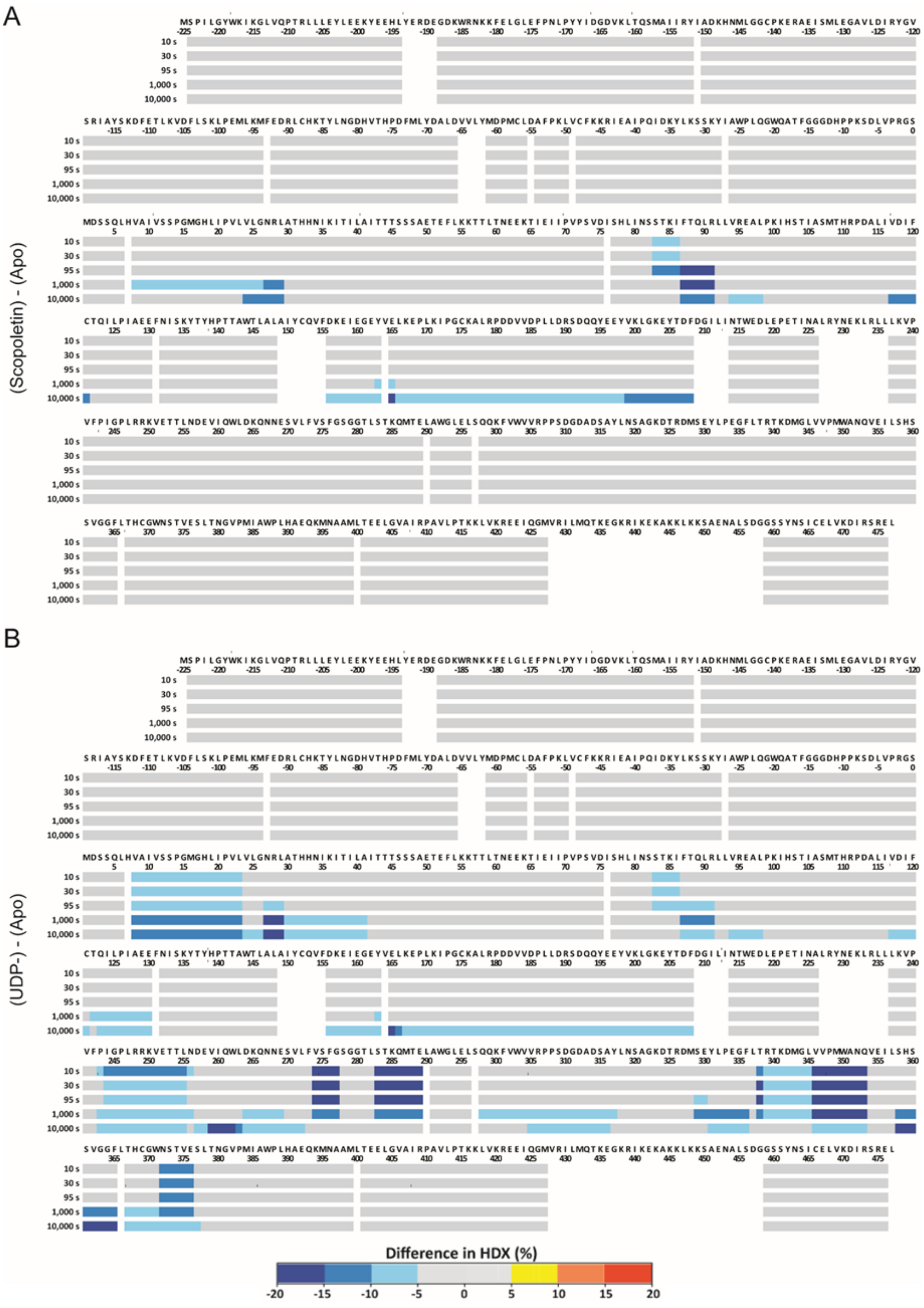

Supplementary Figure 8.

Conformational changes of *Nb*UGT72AY1 caused by substrate binding. (**A**) Differences in deuterium uptake between scopoletin bound and apo *Nb*UGT72AY1. (**B**) Differences in deuterium uptake between UDP bound and apo *Nb*UGT72AY1. Differences displayed on the amino acid sequence of the protein refer to the "Difference in HDX" color bar. Blue boxes show regions with less HDX in the presence of scopoletin or UDP. Regions with increased HDX in the presence of scopoletin or UDP were not observed. Adapted from Liao et al., 2023[3].

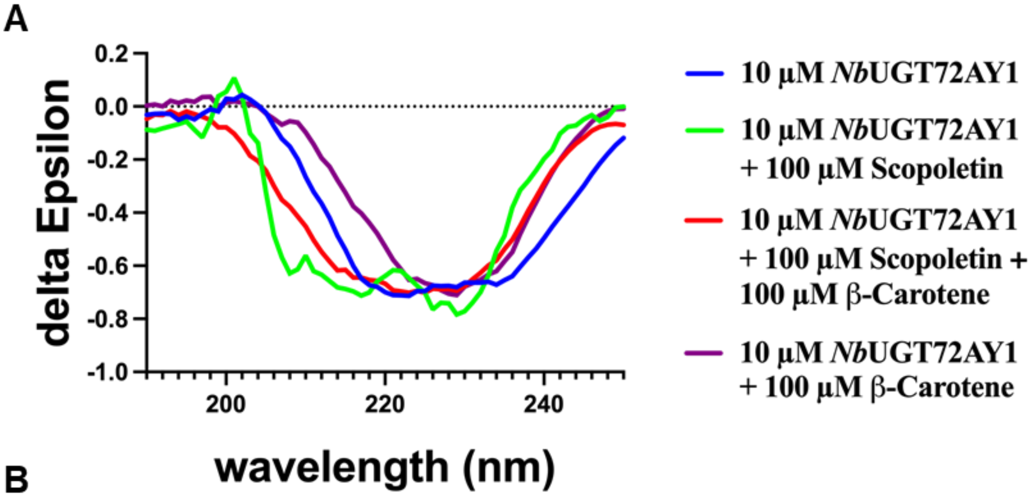

B

CD measurement NbUGT72AY1 + ligands				
**Estimated secondary structure content (%)**	10 µM protein	10 µM protein + 100 µM β-carotene	10 µM protein + 100 µM scopoletin	10 µM protein + 100 µM scopoletin + 100 µM β-carotene
α-Helix	29.0	16.8	31.4	22.5
β-Sheets	28.4	28.6	30.8	23.4
Turn	8.9	18.2	12.8	26.6
Others	33.7	36.4	25.0	27.4

Supplementary Figure 9.
Circular dichroism (CD) spectra of *Nb*UGT72AY1 in the far UV region with respect to different ligand additions. (**A**) CD spectra of *Nb*UGT72AY1 in the presence of various ligands. The results are an average of three repeats each with 10 scans taken and baseline corrected. (**B**) Tabular evaluation of the secondary structure content using the BeStSel CD deconvolution webserver (https://bestsel.elte.hu/index.php).

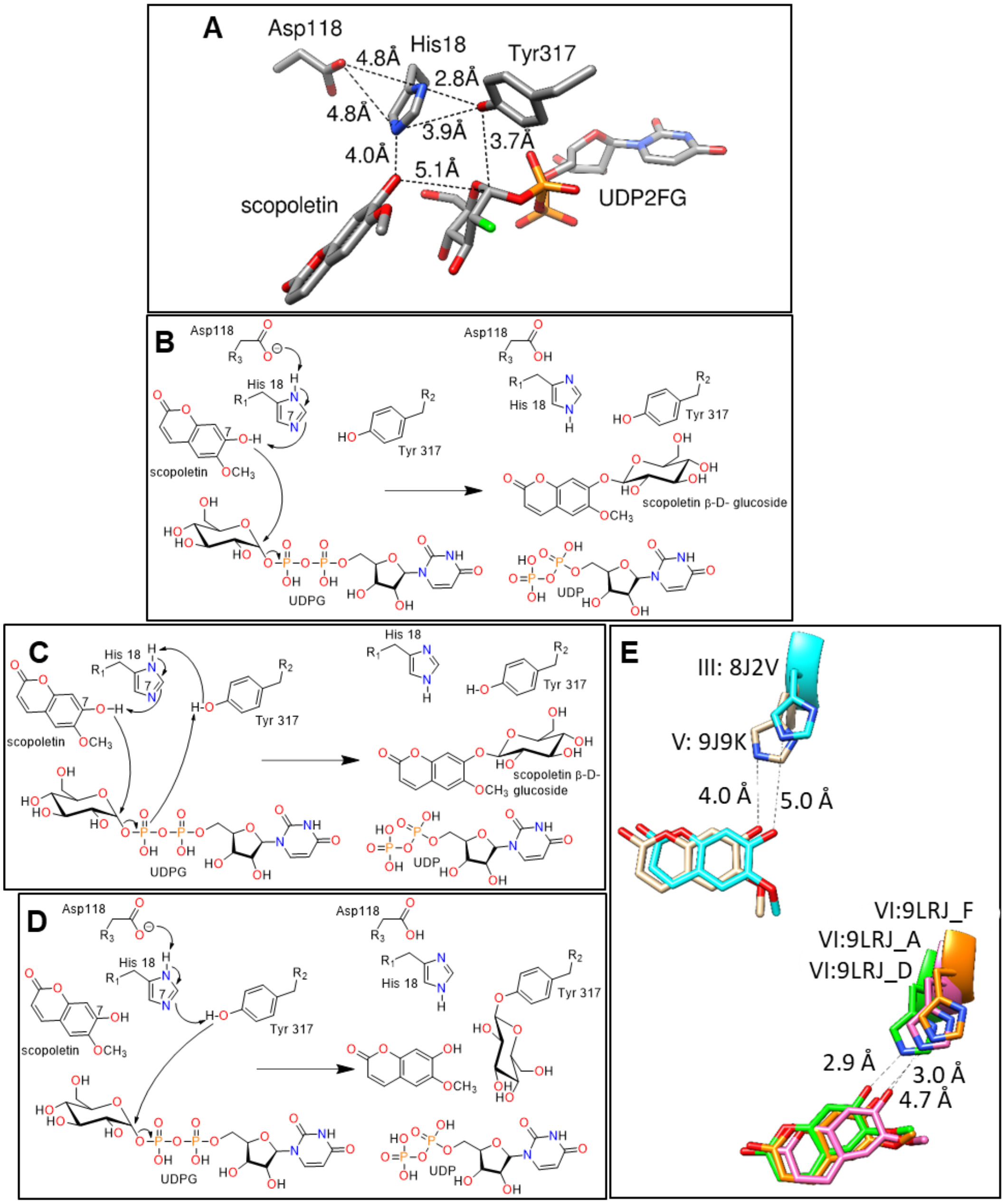

Supplementary Figure 10.

Possible roles of Tyr317 in the catalytic cycle of *Nb*UGT72AY1. (**A**) Arrangement of catalytically active His18, Tyr317 (amino acid of the closing loop), acceptor and donor substrate in the active site (based on V: *Nb*UGT72AY1•scopoletin/UDP2FG: 9J9K). (**B**) Classical reaction in which His18 serves as proton acceptor and enables nucleophilic attack of O7 of scopoletin at C1 of UDPG, whereas Asp118 activates His18 by deprotonation (**C**) Alternatively, Tyr317 could also activate His18 by transferring a proton from His18 to the β-phosphate group of UDPG. (**D**) Proposed inhibitory effect of Tyr317. Tyr317 could serve as alternative sugar acceptor. (**E**) Distance of His18-N7 to Scopoletin-7-OH in 3D structures of *Nb*UGT72AY1 that were crystallized with scopoletin. Left without (apo)carotenoid, right with retinol.

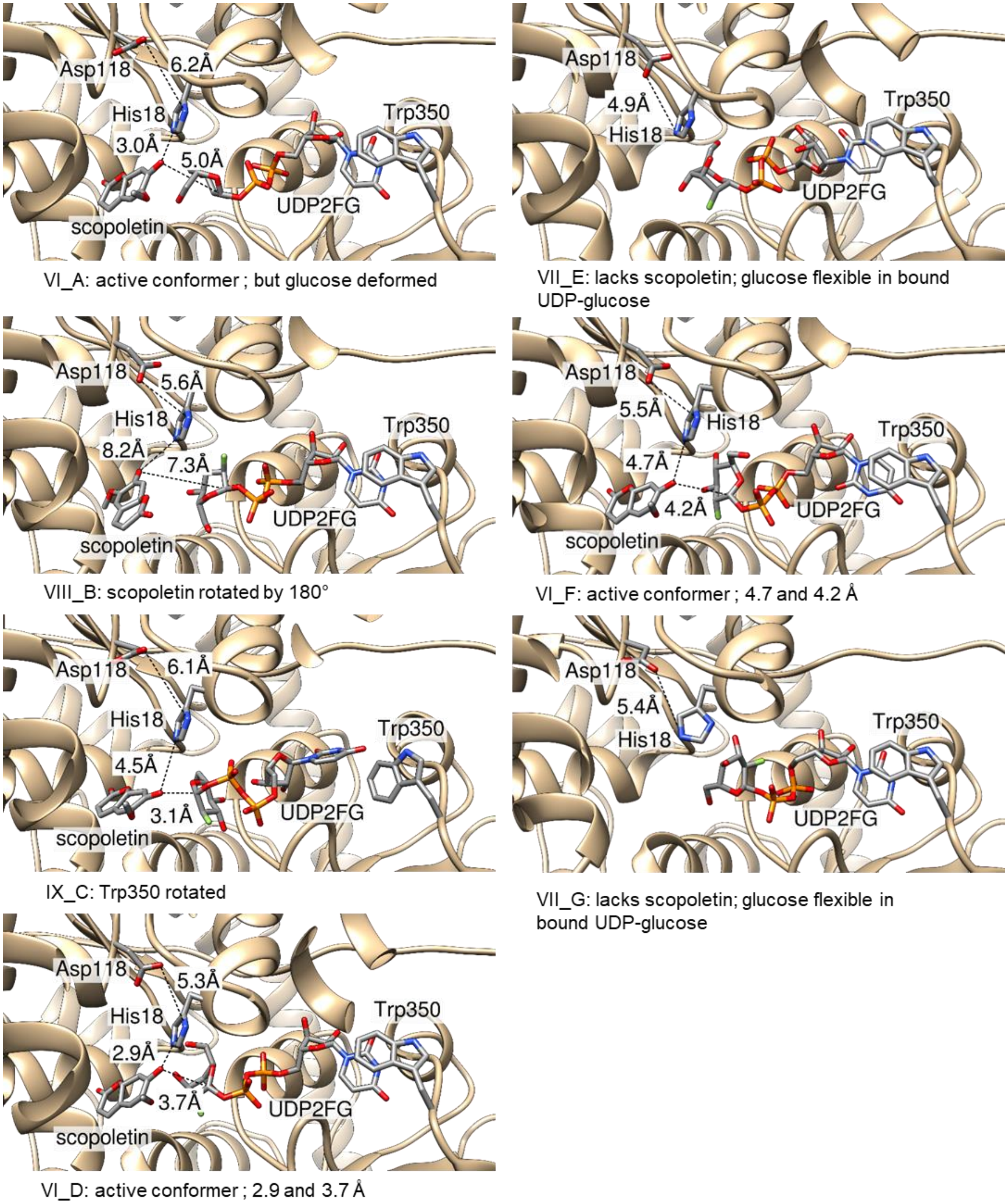

Supplementary Figure 11.

Close up of various complexes in 9LRJ. The distances (between Asp118-O4 and His18-N5, His18-N5 and scopoletin-N7, scopoletin-N7 and UDP2FG-C1) in the active site of seven *Nb*UGT72AY1 complexes obtained by co-crystallization of retinol, scopoletin and UDP2FG (9LRJ).

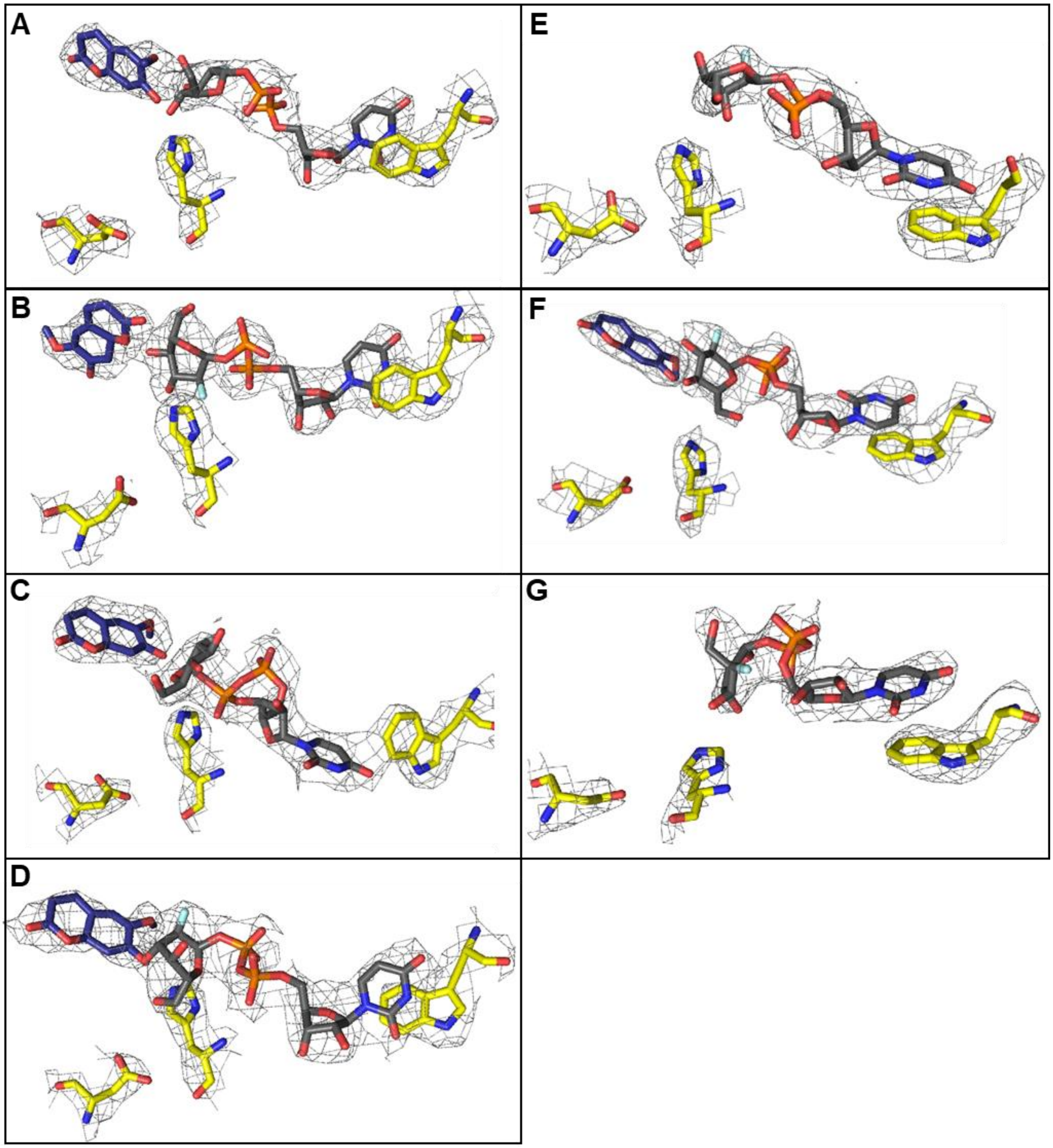

Supplementary Figure 12.

Close up of various complexes in 9LRJ. (**A-G**) $2F_o$-F_c omit map at 1 **σ** cutoff for the Supplementary Figure 11, where scopoletin (blue) and UDP2FG (gray) are shown as sticks and the residues H18, D118, and W350 are shown as yellow sticks.

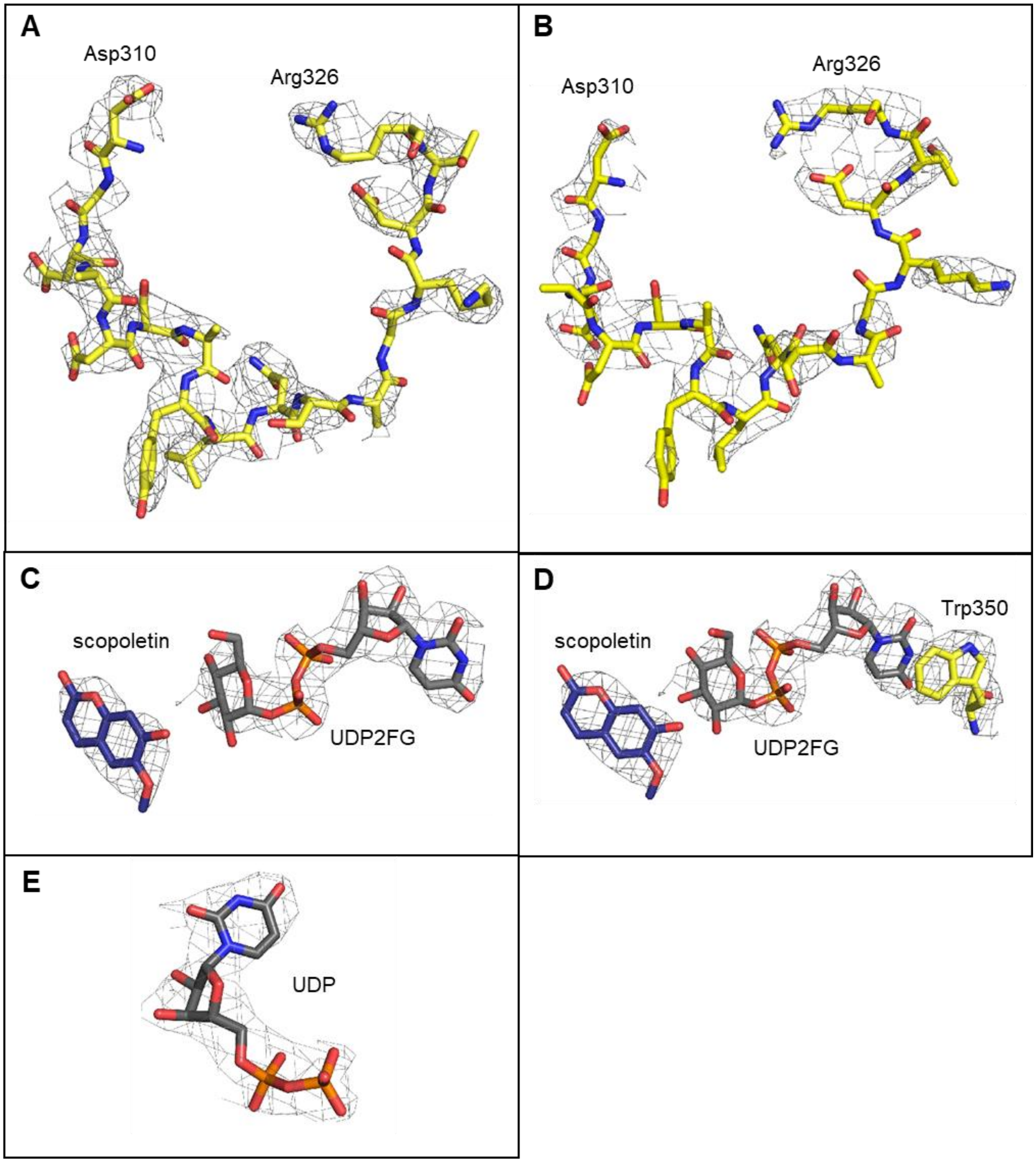

Supplementary Figure 13.

Electron density for the closing loop (310-326) and ligands in substrate-inhibited 9J9K and product-inhibited 8J2U. (**A**) $2F_o$-F_c omit map at 1 **σ** cutoff for the residues 310-326 in the Scopoletin/UDP2FG bound structure 9J9K. (**B**) $2F_o$-F_c omit map at 1 **σ** cutoff for the residues 310-326 in the UDP bound structure 8J2U. (**C**) $2F_o$-F_c omit map at 1 **σ** cutoff for the scopoletin (blue stick) and UDP2FG (gray stick) bound to the protein in 9J9K. (**D**) $2F_o$-F_c omit map at 1 **σ** cutoff for the Trp350 (yellow stick) in proximity to UDP2FG (gray stick) in 9J9K. (**E**) $2F_o$-F_c omit map at 1 **σ** cutoff for the UDP (gray stick) bound to the protein in 8J2U.

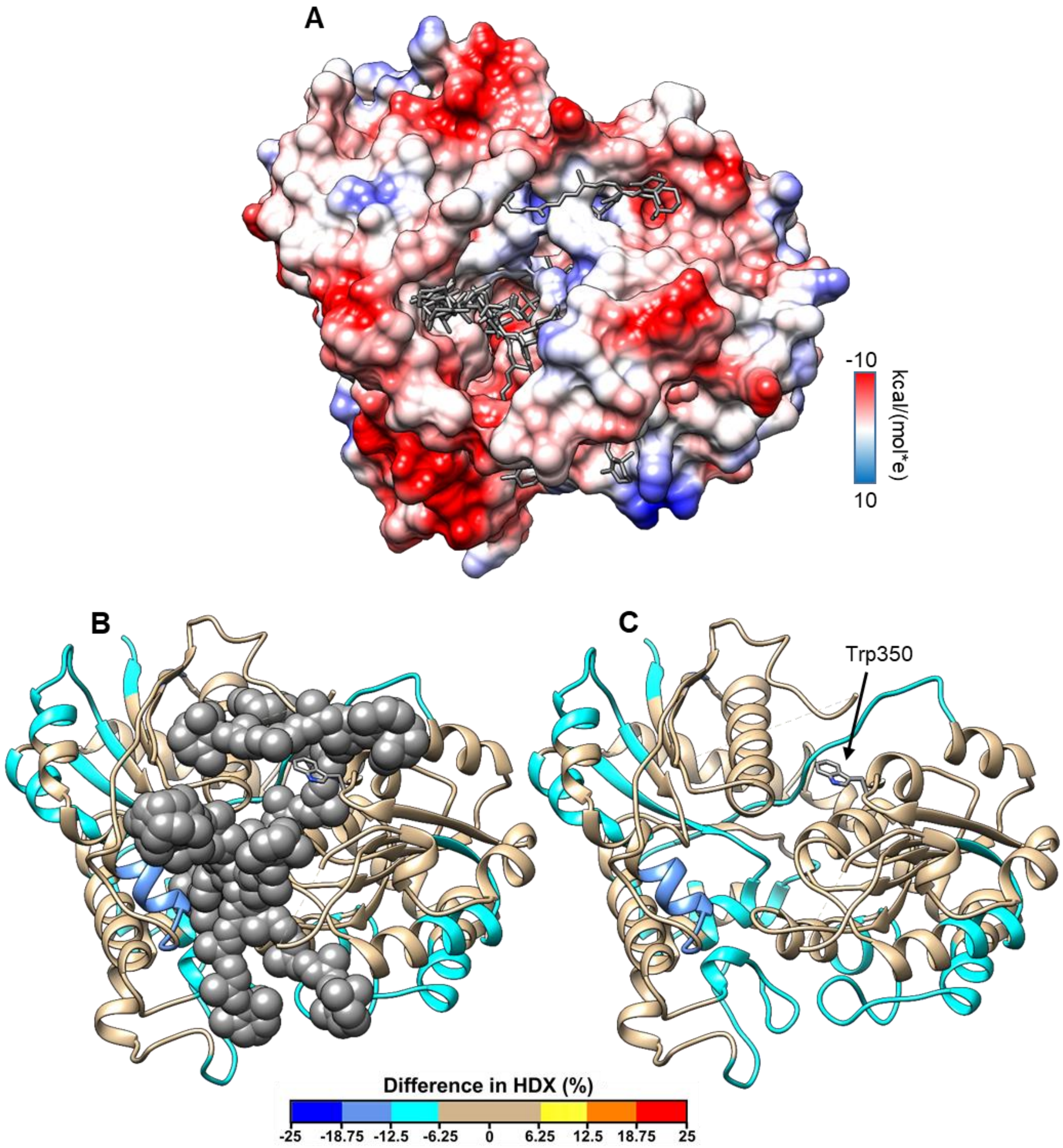

Supplementary Figure 14.

Computer-assisted molecular docking of β-carotene to predict the binding modes of the ligand β-carotene. (**A**) β-carotene (in stick presentation) docking was performed on the 3D structure obtained in the presence of β-carotene (complex II**;** missing loops added my Modeller implemented in Chimera) by Autodock Vina implemented in Chimera. Binding scores corresponding to binding energies of -9.6 to -8.0 kcal/mol were calculated. The coloring of the coulombic surface represents the electrostatic potential from -10 (red) through 0 (white) to 10 kcal/(mol*e) (blue). (**B**) Binding modes of β-carotene (sphere presentation) in the 3D structure (complex **II**) showing the differential hydrogen/deuterium exchange (HDX) results obtained after the addition of β-carotene to *Nb*UGT72AY1. (**C**) 3D-structure showing the HDX result.

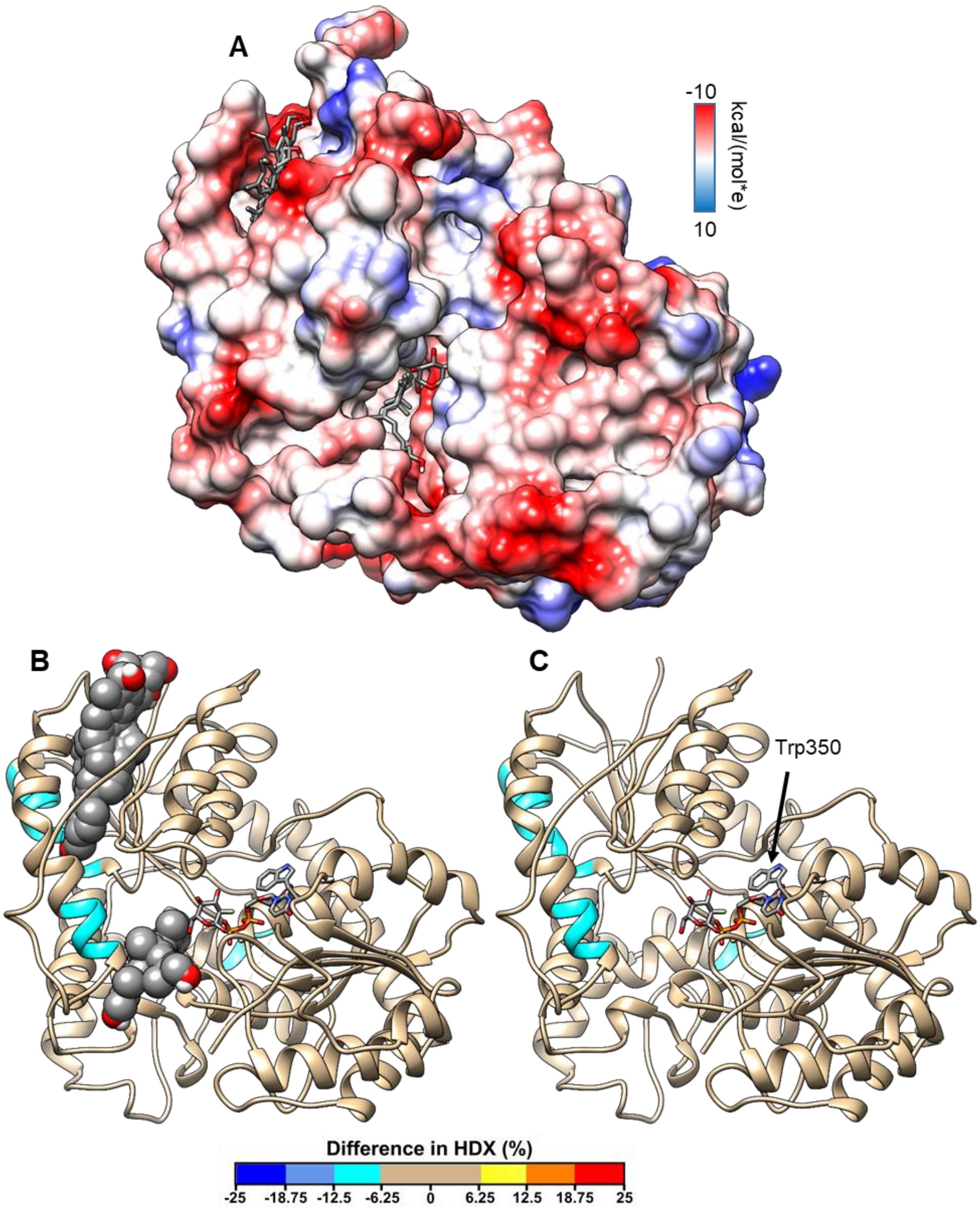

Supplementary Figure 15.

Computer-assisted molecular docking of retinol to predict the binding modes of the ligand retinol. (**A**) Retinol (in stick presentation) docking was performed on the 3D structure obtained in the presence of retinol and UDP2FG/(scopoletin) (complex VII**;** missing loops added my Modeller implemented in Chimera) by Autodock Vina implemented in Chimera. Binding scores corresponding to binding energies of -9.1 to -7.0 kcal/mol were calculated. The coloring of the coulombic surface represents the electrostatic potential from -10 (red) through 0 (white) to 10 kcal/(mol*e) (blue). (**B**) Binding modes of retinol (sphere presentation) in the 3D structure (complex VII) showing the differential hydrogen/deuterium exchange (HDX) results obtained after the addition of retinol to *Nb*UGT72AY1. (**C**) 3D-structure showing the HDX result.

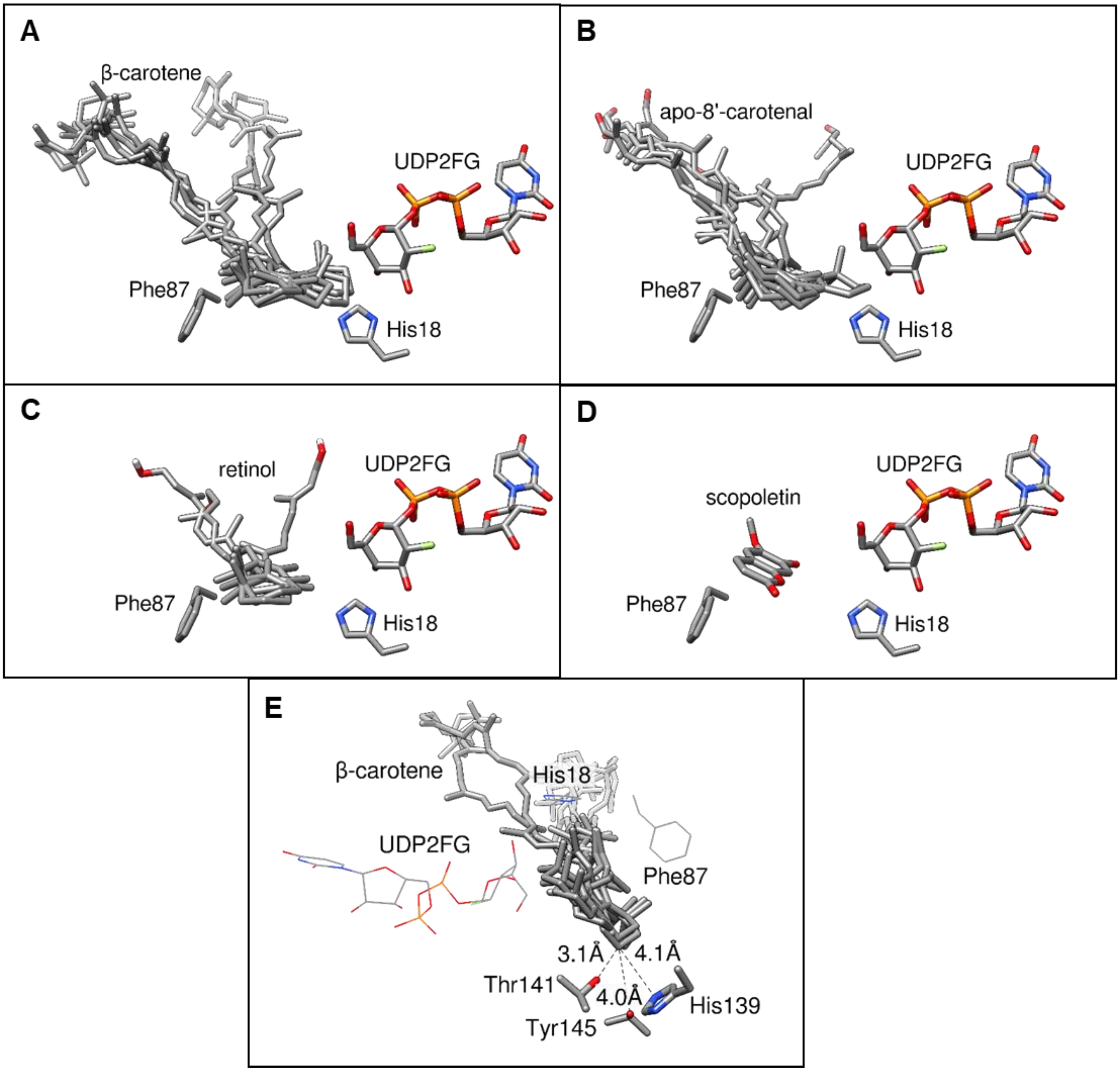

Supplementary Figure 16.

Computer-assisted molecular docking. (**A**) β-carotene, (**B**) apo-8‘-carotenal, and (**C**) retinol were docked on complex VII (*Nb*UGT72AY1•UDP2FG/(scopoletin)/(retinol): 9LRJ_G) to predict the binding modes of the ligands. (**D**) The scopoletin binding site in III (*Nb*UGT72AY1•scopoletin: 8J2V) was superimposed to visualize the acceptor in the active site. (**E**) His139, Tyr141, and Tyr145 are in close proximity to C3/C3‘ of β-carotene, the carbon atom to which -OH is bonded in zeaxanthin and lutein.

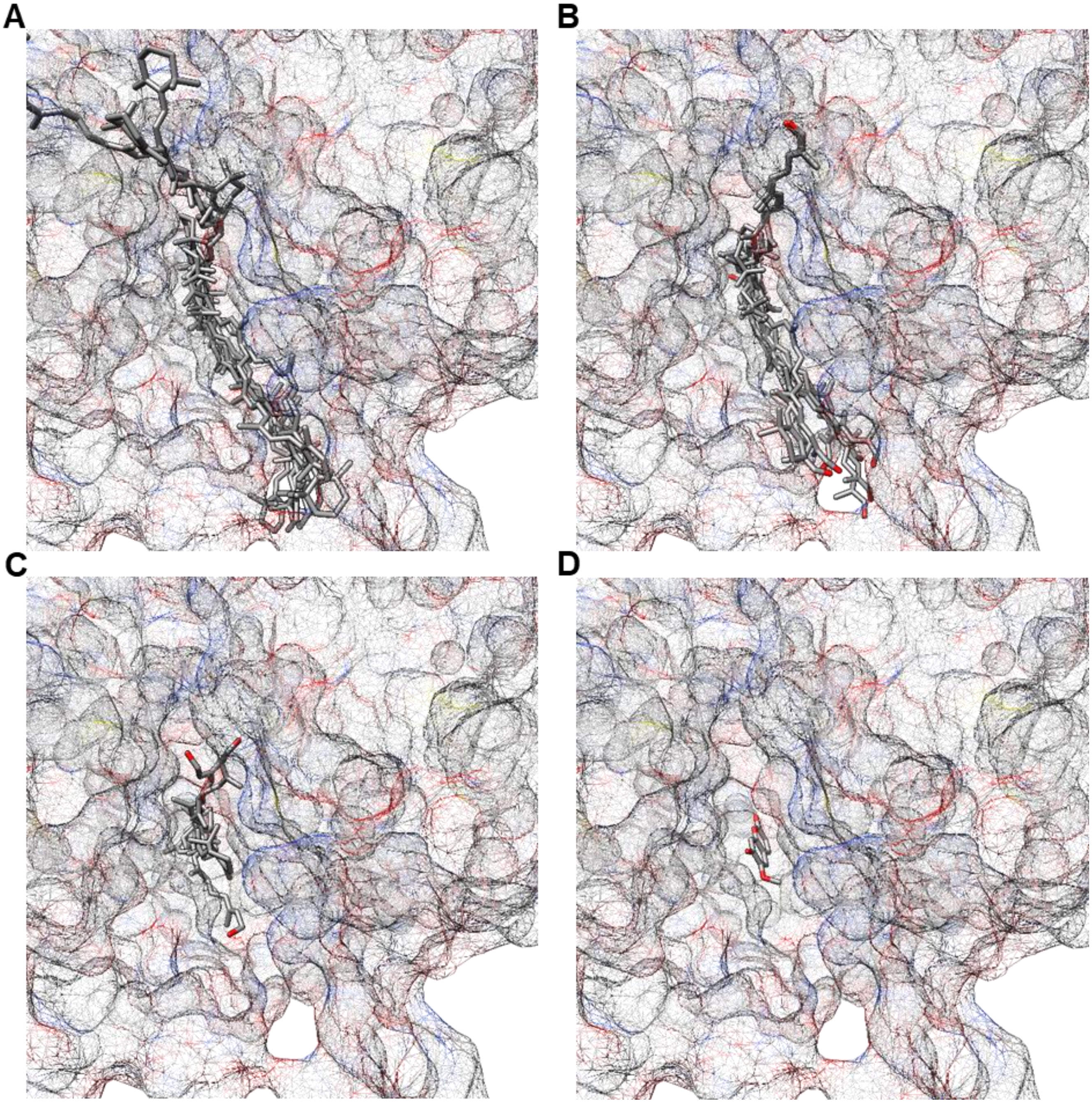

Supplementary Figure 17.

The effectors β-carotene, apo-8‘-carotenal, and retinol occupy the scopoletin binding site in *Nb*UGT72AY1. (**A**) β-carotene, (**B**) apo-8‘-carotenal, and (**C**) retinol were docked on complex VII (*Nb*UGT72AY1•UDP2FG/(scopoletin)/(retinol): 9LRJ_G) and (**D**) scopoletin (III: *Nb*UGT72AY1•scopoletin: 8J2V) was superimposed. The surface is shown as mesh and colored according to heteroatom: carbon gray, oxygen red, nitrogen blue and sulfur yellow.

A

structure	Trp350	Loop/helix transition	Loop open/closed
Apo (I)	Upright	Loop (not resolved)	Not resolved
+β-carotene (II)	Upright, rotated	Loop (not resolved)	Not resolved
+UDP (IV)	Parallel to uracil plane, π-π	Helix	Closed
+UDP2FG/scopoletin (V)	Parallel to uracil plane, π-π	Helix	Closed
+scopoletin (III)	Parallel to uracil plane, rotated	Helix	Not resolved
+UDP2FG/scopoletin/(retinol) (VI)	Parallel to uracil plane, π-π	Helix	Not resolved
+UDP2FG/(retinol) (VII)	Parallel to uracil plane, π-π	Helix	Not resolved

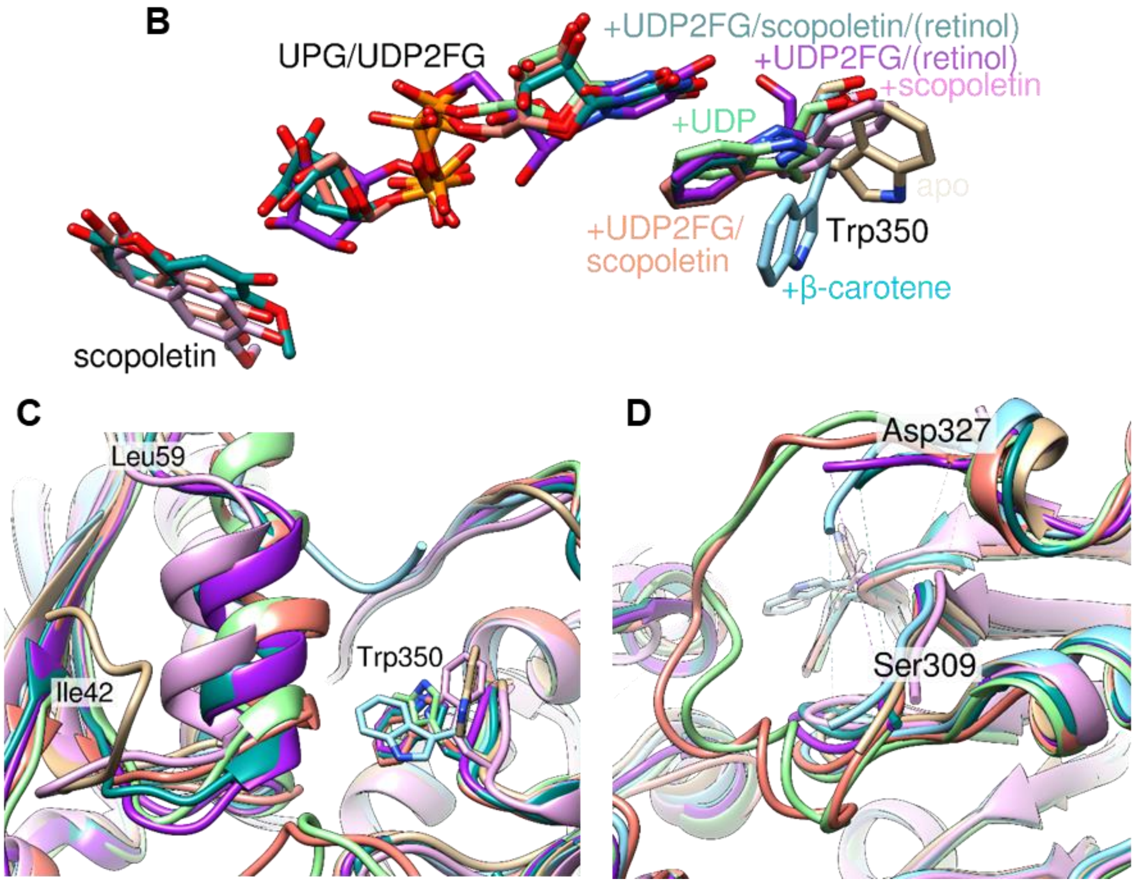

Supplementary Figure 18.

Summary of comparative analysis of *Nb*UGT72AY1 structures obtained with different ligands. (**A**) Table showing the most prominent differences between the 3D structures. (**B**) Structural overlay illustrating the spatial orientation of the Trp350 side chains together with respective ligands. (**C**) Structural overlay demonstrating the loop-helix transition between Ile42 and Leu59. (**D**) Structural overlay of the resolved residues of the closing loop (Ser309-Asp327). Color code is identical for **B**, **C**, and **D**.

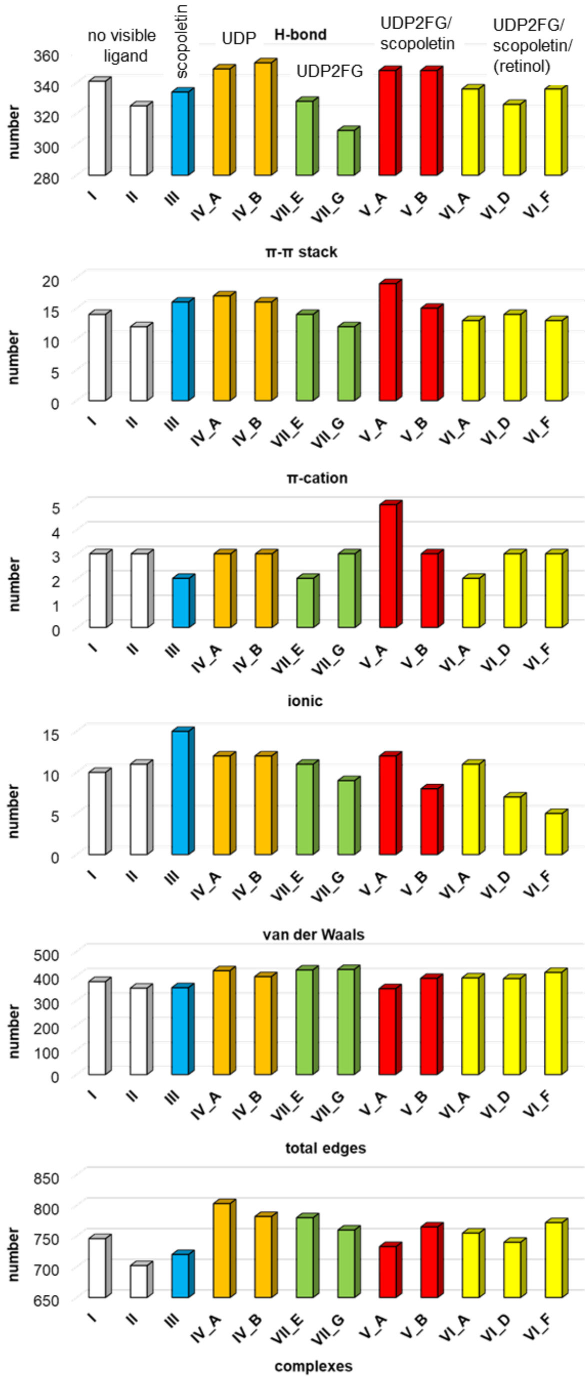

Supplementary Figure 19.

Summary of the calculation of non-covalent bonds in *Nb*UGT72AY1 structures and complexes. The number of H-, π-π stack, π-cation, ionic, and van der Waals bonds was determined using the RING server (https://ring.biocomputingup.it/)[14]. The complexes are grouped according to the number of bound ligands.

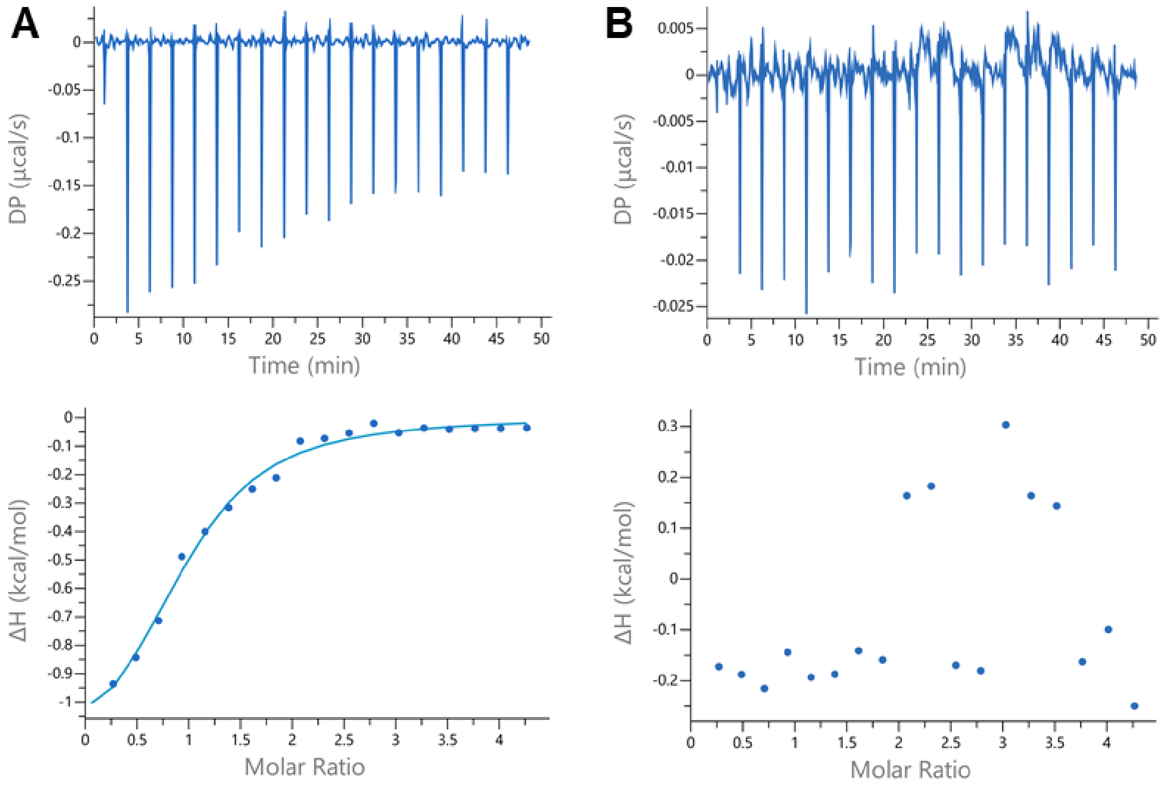

C

cell	syringe	N	K_d (µM)	ΔG (kcal/mol)	ΔH (kcal/mol)	-TΔS (kcal/mol/K)
*Nb*UGT72AY1	Scopoletin	0.92 ± 0.10	4.45 ± 1.12	-7.30 ± 0.95	-1.29 ± 0.15	-6.01 ± 0.75

Supplementary Figure 20.

Isothermal titration calorimetry (ITC) measurements of *Nb*UGT72AY1 and scopoletin. (**A**) The raw heat plot shows exothermic titration peak signals for each ligand injection into the protein inside the cell, and the lower plot shows the heat integration for the above panel. (**B**) The titration of ligand into the buffer. (**C**) Calculated parameters from the ITC measurements.

A

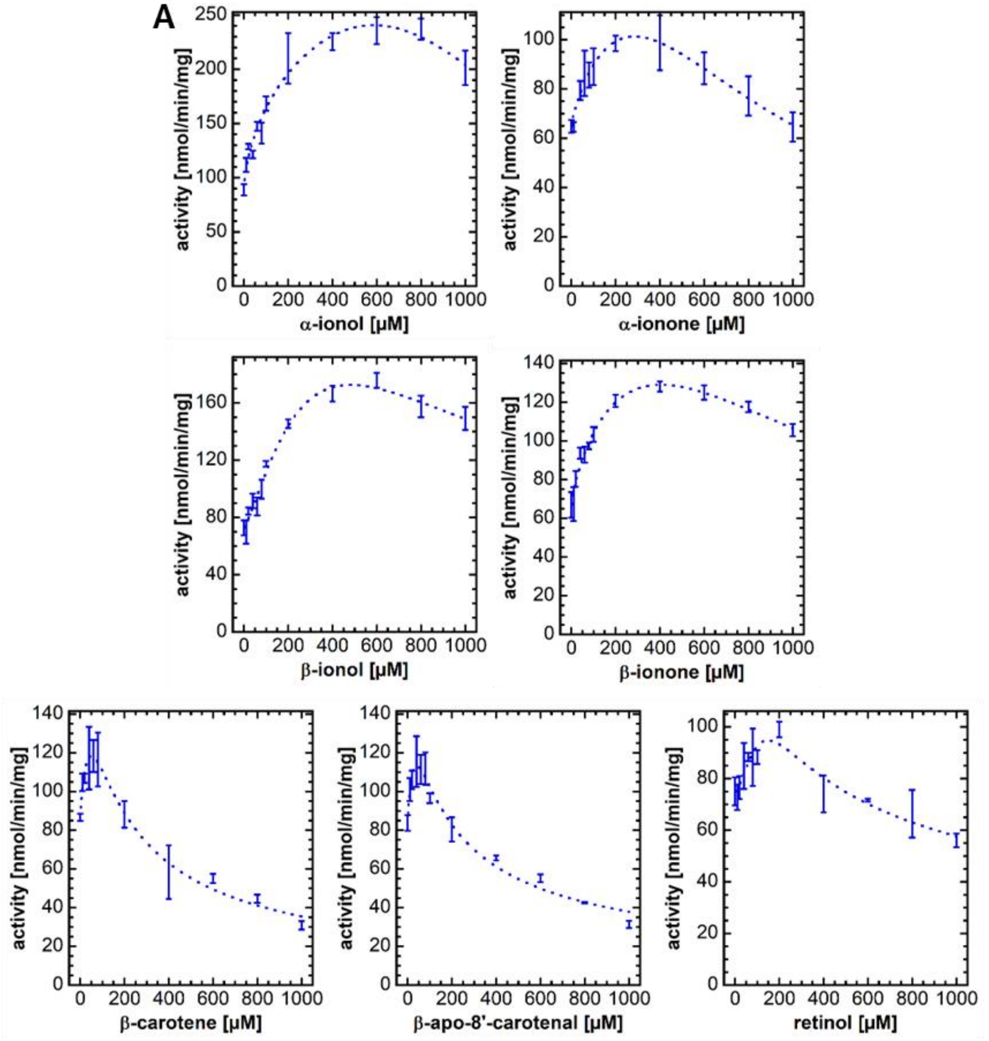

B

	v_{max}	± error	K_D	± error	n	± error	x	± error	R^2	Chi square
β-carotene	249	18	94	2	1.0	0.2	1.7	0.2	0.99	113
β-apo-8'-apocarotenal	243	21	73	30	1.0	0.3	1.6	0.3	0.98	166
retinol	208	14	179	79	1.7	0.7	2.3	0.6	0.91	134
α-ionol	513	47	1023	95	0.7	0.1	3.4	1.4	0.97	814
α-ionone	208	17	557	108	0.9	0.1	2.2	0.2	0.97	41
β-ionol	443	12	535	185	1.2	0.2	2.1	0.2	0.98	255
β-ionone	295	21	705	185	0.8	0.1	1.9	0.3	0.98	98

Supplementary Figure 21.

Impact of effectors on the scopoletin:UDP-glucose glucosyltransferase of NbUGT72AY1. (**A**) Different concentrations of α-ionol, α-ionone, β-ionol, β-ionone, β-carotene, β-apo-8'-carotenal, and retinol were added to enzyme assays consisting of 200 µM scopoletin, 100 µM UDPG and 0.5 µg NbUGT72AY1 to determine the effect on UGT activity. Reaction rates were determined using the UDP-GloTM assay. (**B**) Equation 2 was used to fit the curves. Source data are provided as a Source Data file. n=5 independent experiments.

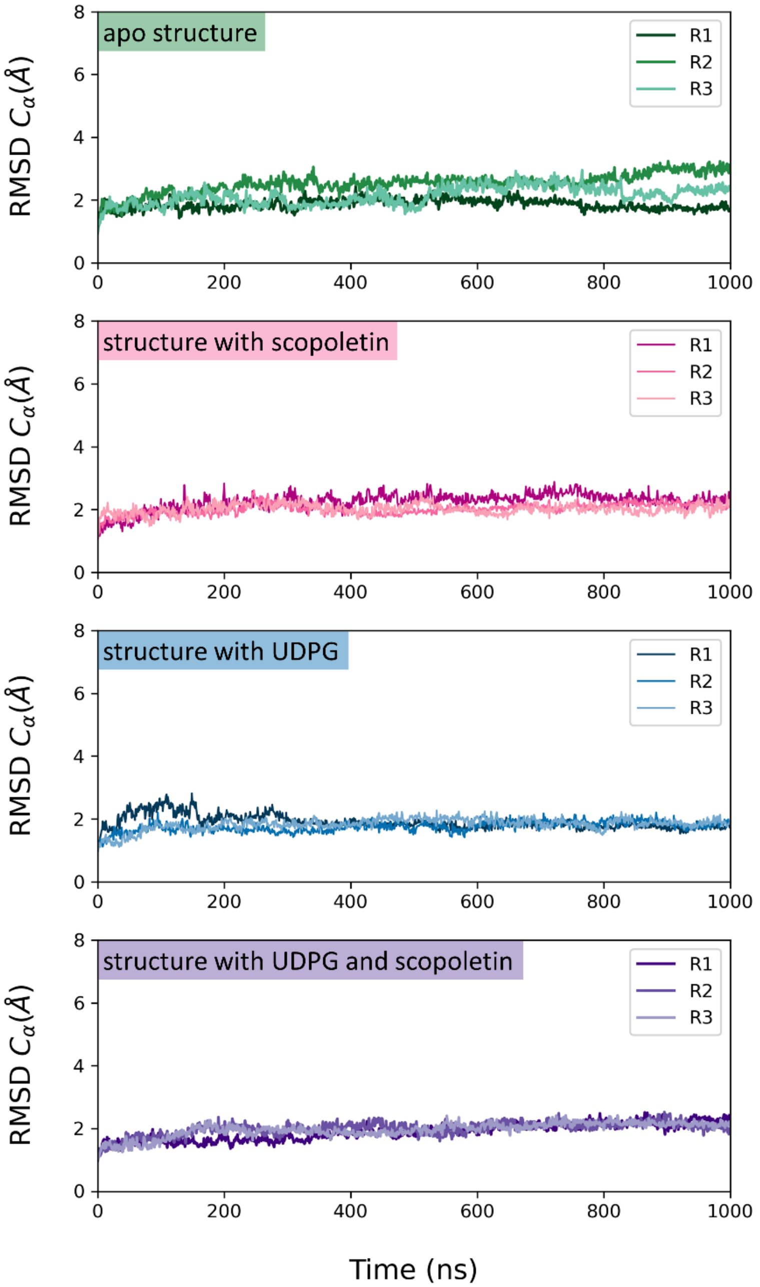

Supplementary Figure 22.

Root Mean Square Deviation (RMSD) of the backbone Cα during the MD simulations of *Nb*UGT72AY1. Four systems were investigated. *Nb*UGT72AY1 (PDB 9J9K, complex V) without ligands, in complex only with scopoletin, in complex only with UDPG, and in complex with UDPG and scopoletin. R1, R2, and R3 denote replicates.

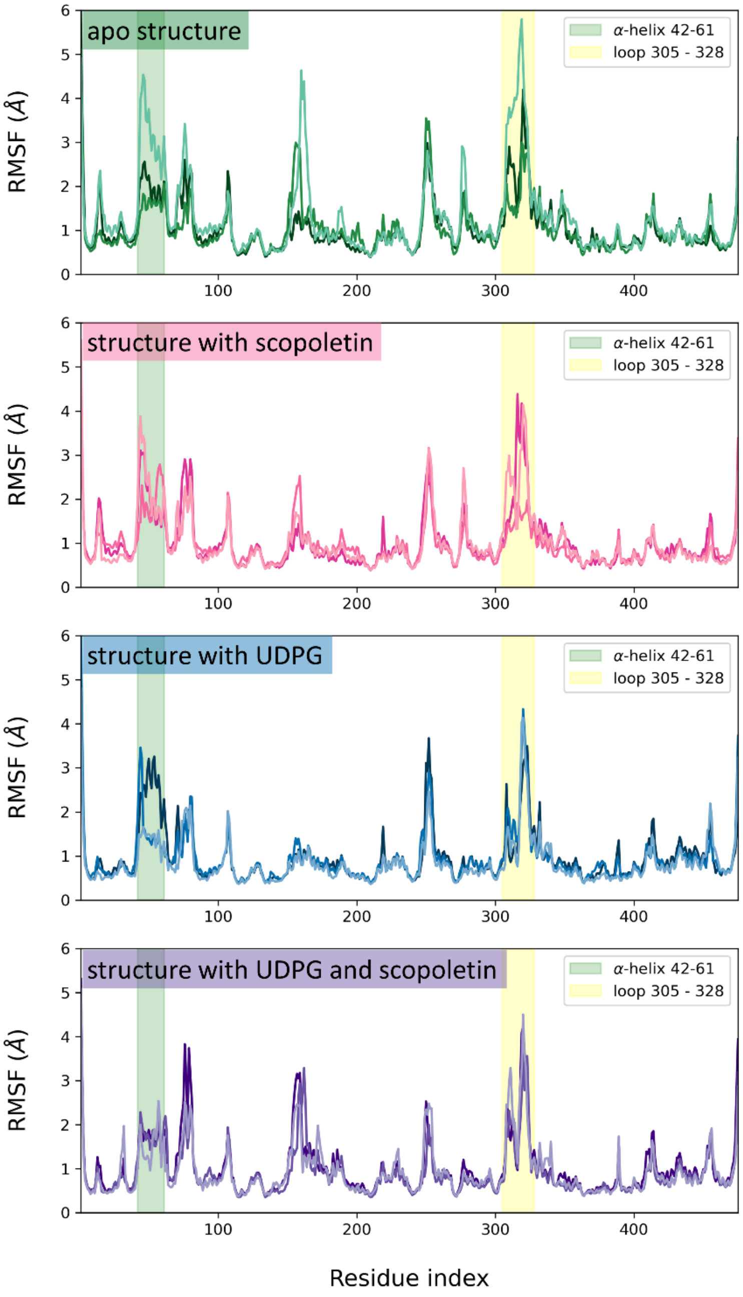

Supplementary Figure 23.

Root mean square fluctuation analysis (RMSF) of the backbone Cα during the MD simulations of *Nb*UGT72AY1. For details of the systems, see Supplementary Figure 22. Three replicates were carried out.

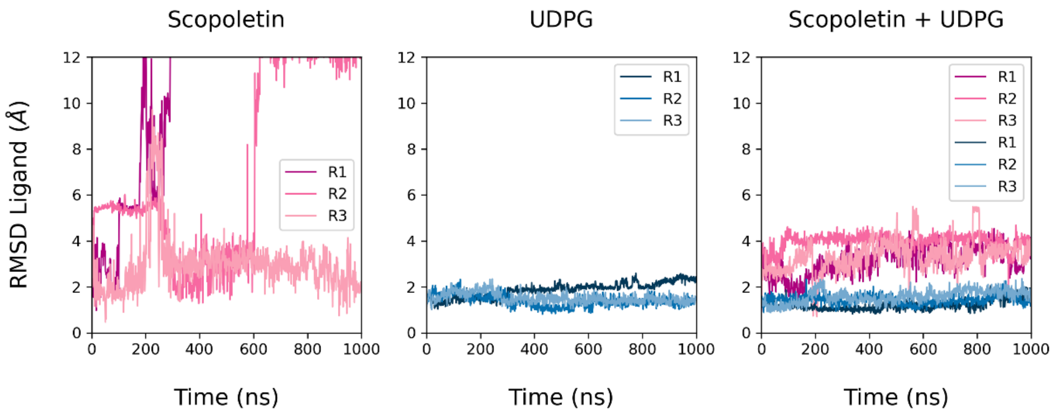

Supplementary Figure 24.

RMSD plots of heavy ligand atoms during the MD simulations of the ligand-bound systems *Nb*UGT72AY1 (PDB 9J9K, complex V) in complex with only scopoletin, with only UDPG, with scopoletin and UDPG. R1, R2, and R3 denote replicates.

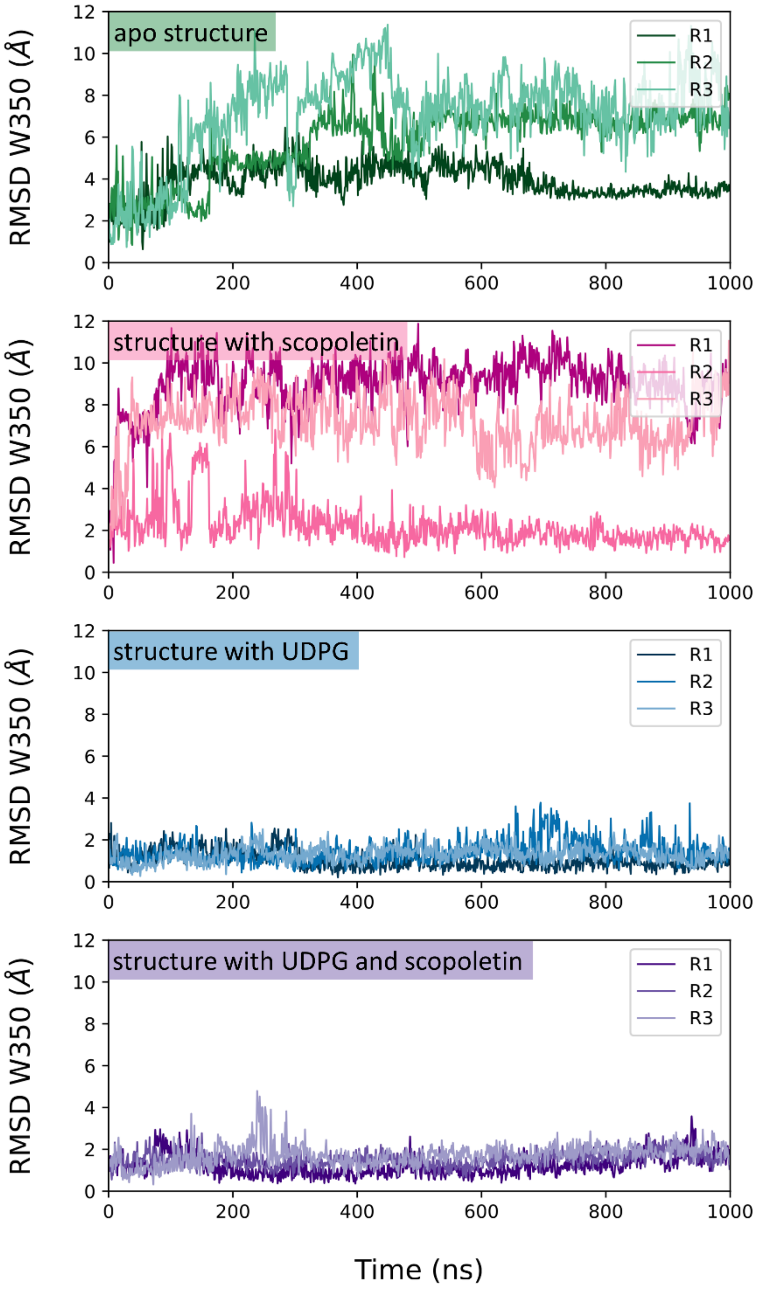

Supplementary Figure 25.

RMSD plots of Trp350 heavy atoms during the MD simulations. For details of the systems, see Supplementary Figure 22. Three replicates were carried out.

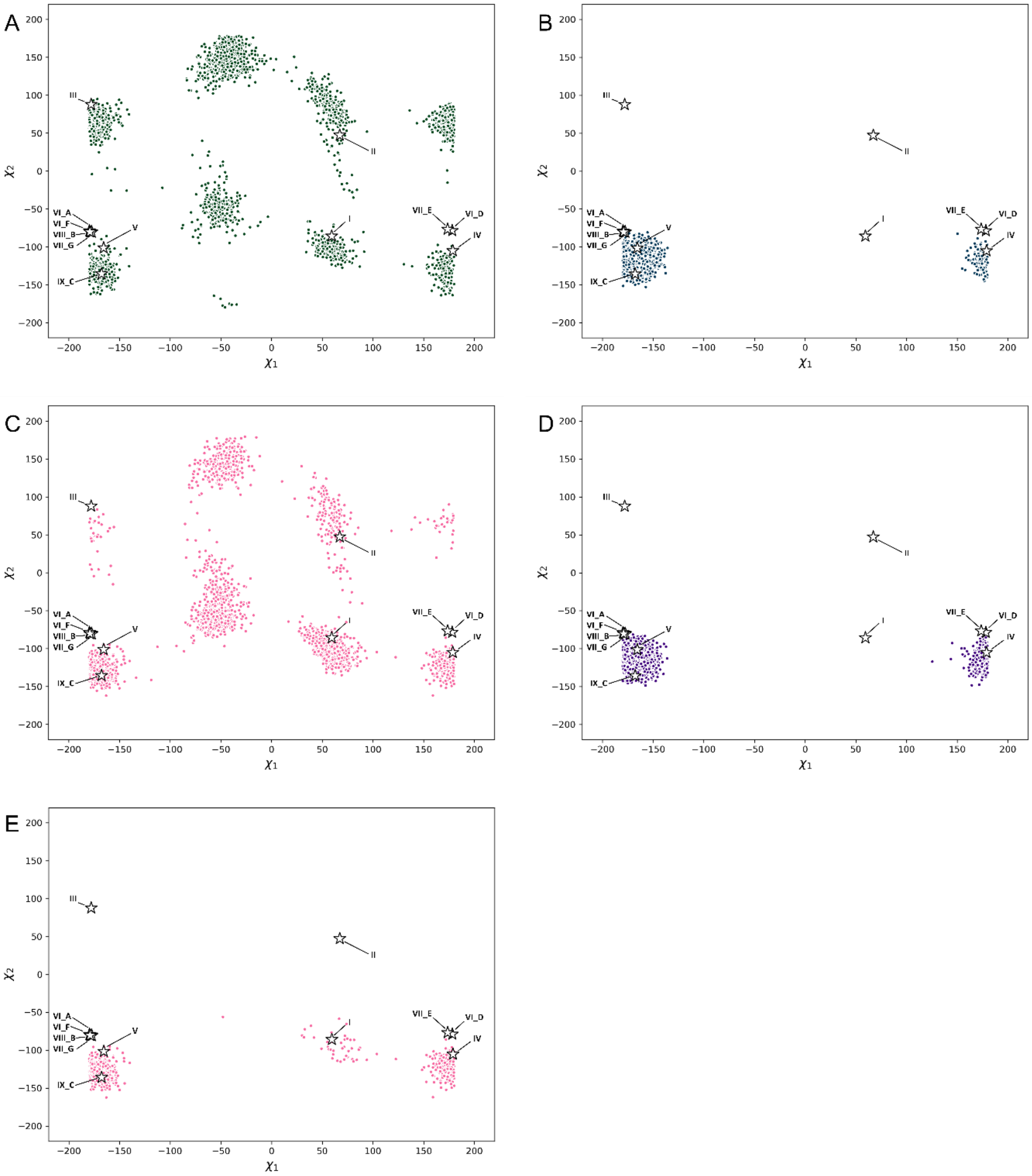

Supplementary Figure 26.

Chi1 (χ_1) and chi2 (χ_2) torsion angle analysis of Trp350. Torsion angles values are represented as green, magenta, blue, and purple dots. MD frames of the (**A**) apo protein (from complex V), (**B**) the protein (from complex **V**) in complex with UDPG, (**C**) the protein (from complex V) with scopoletin, and (**D**) the protein (from complex V) with scopoletin and UDPG. (**E**) Torsion angle analysis of Trp350 of the complex with scopoletin using only replica 2, where scopoletin stays in the binding pocket for the entire trajectory. Stars indicate chi1 (χ_1) and chi2 (χ_2) values as measured in the experimental structures (for details of the systems, see Figure 2).

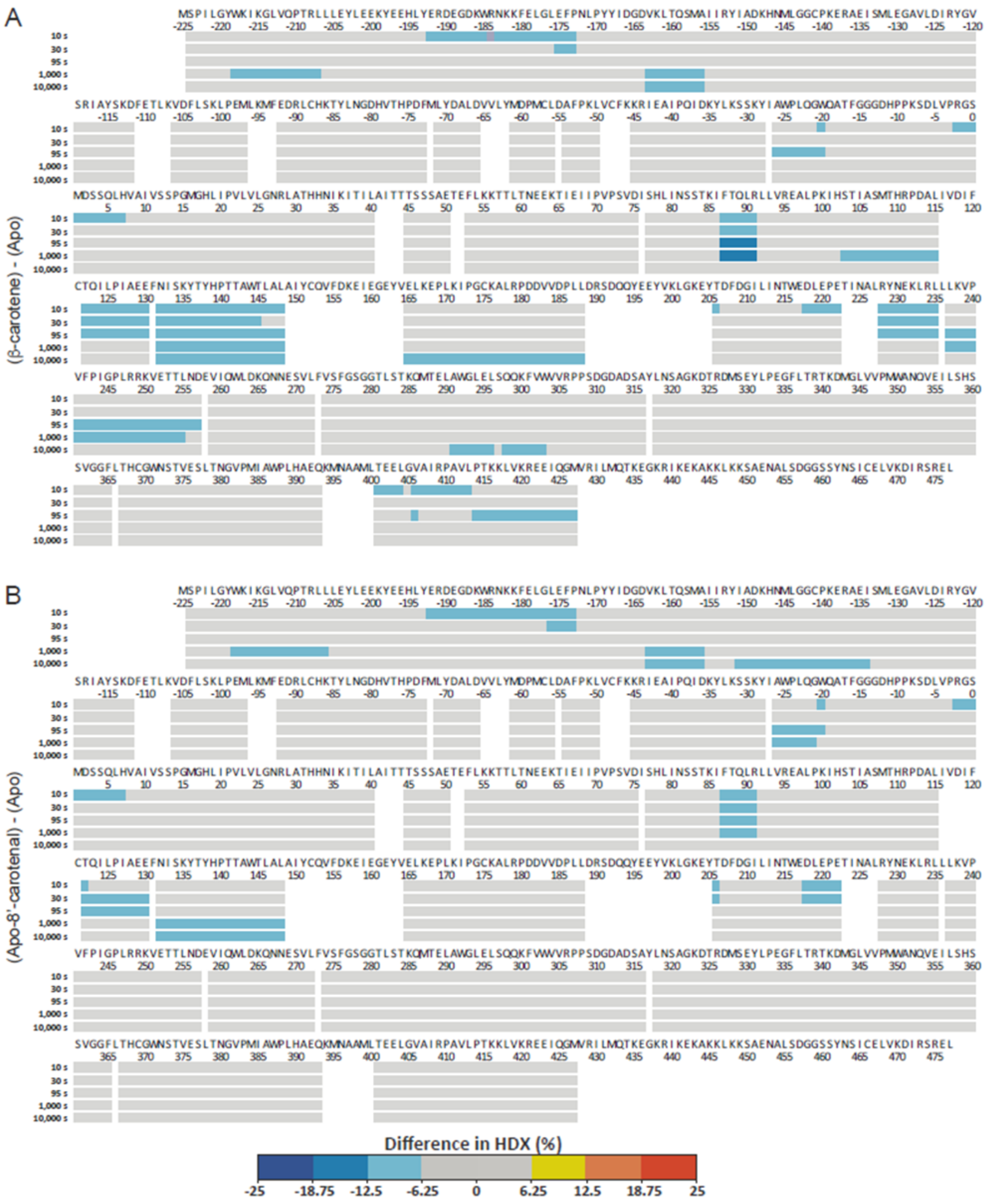

Supplementary Figure 27.

Difference in deuterium uptake by *Nb*UGT72AY1 as a consequence of ligand binding. Difference in deuterium uptake between (**A**) β-carotene- and (**B**) β-apo-8‘-carotenal-bound and apo *Nb*UGT72AY1. Blue color denotes reduced deuterium uptake in presence of ligand.

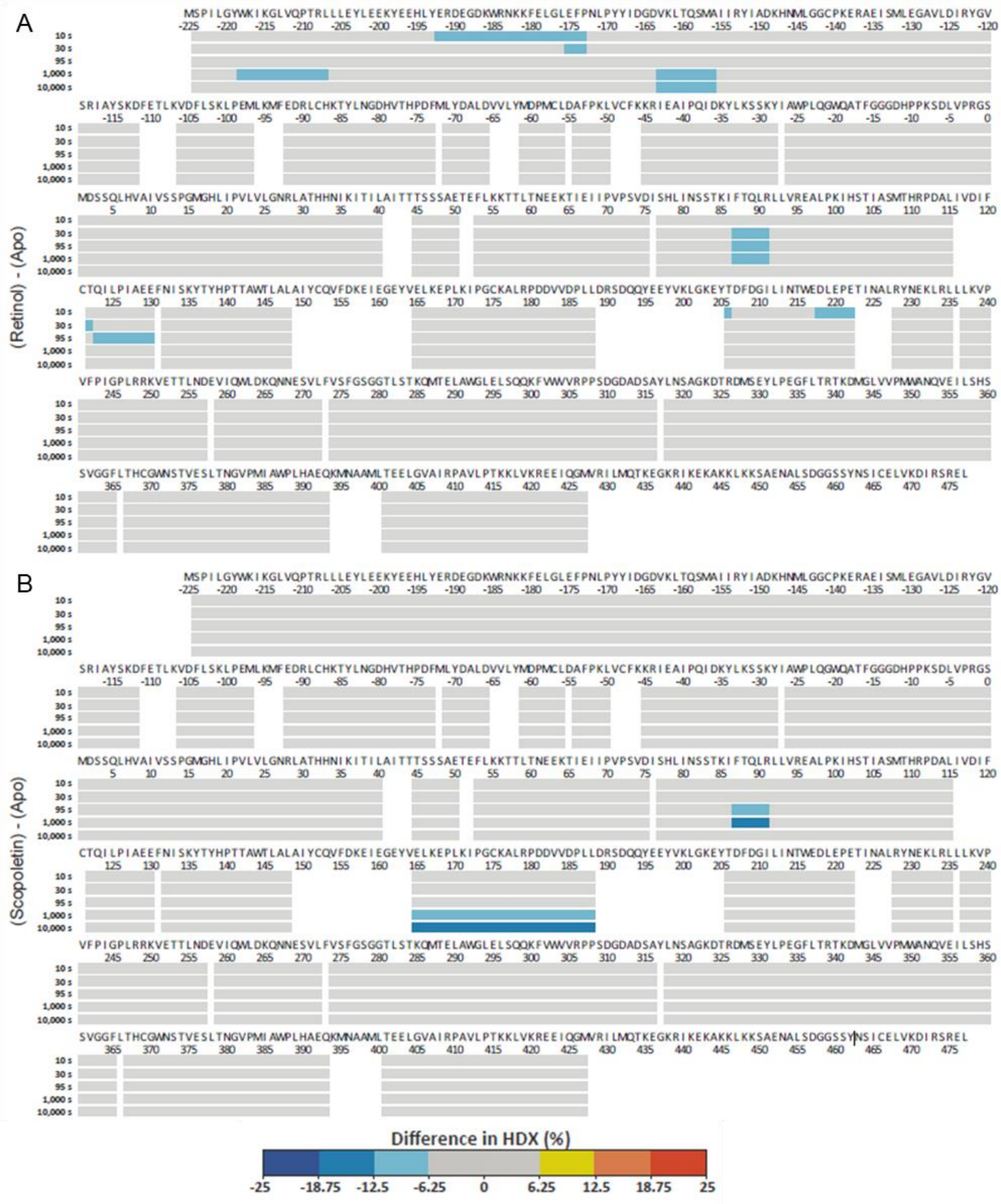

Supplementary Figure 28.

Difference in deuterium uptake by *Nb*UGT72AY1 as a consequence of ligand binding. Difference in deuterium uptake between (**A**) retinol- and (**B**) scopoletin-bound and apo *Nb*UGT72AY1. Blue color denotes reduced deuterium uptake in presence of ligand.

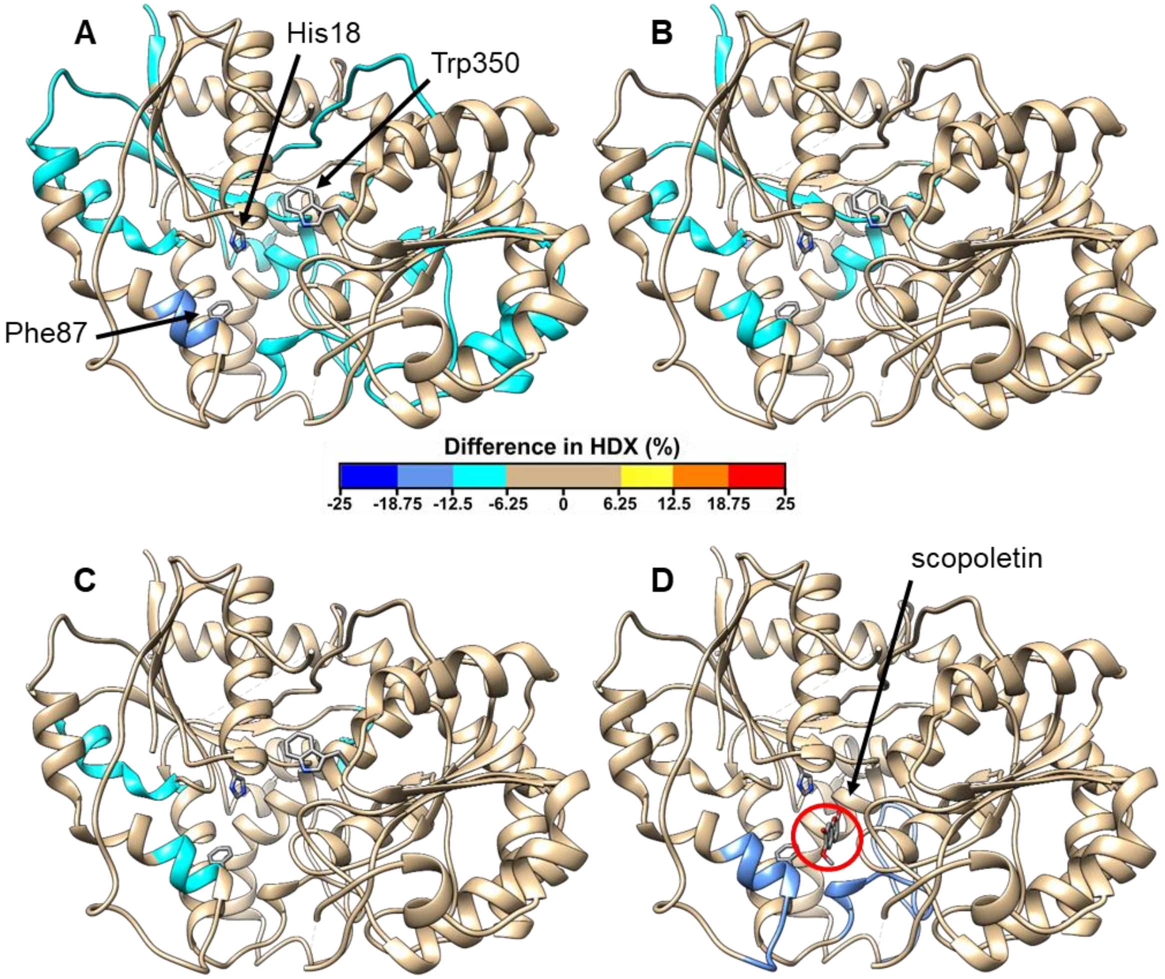

Supplementary Figure 29.

Difference in deuterium uptake by *Nb*UGT72AY1 as a consequence of ligand binding. Difference in deuterium uptake between (**A**) β-carotene-, (**B**) β-apo-8'-carotenal-, (**C**) retinol- and (**D**) scopoletin-bound and apo *Nb*UGT72AY1. Differences are displayed on structure II in form of a color code.

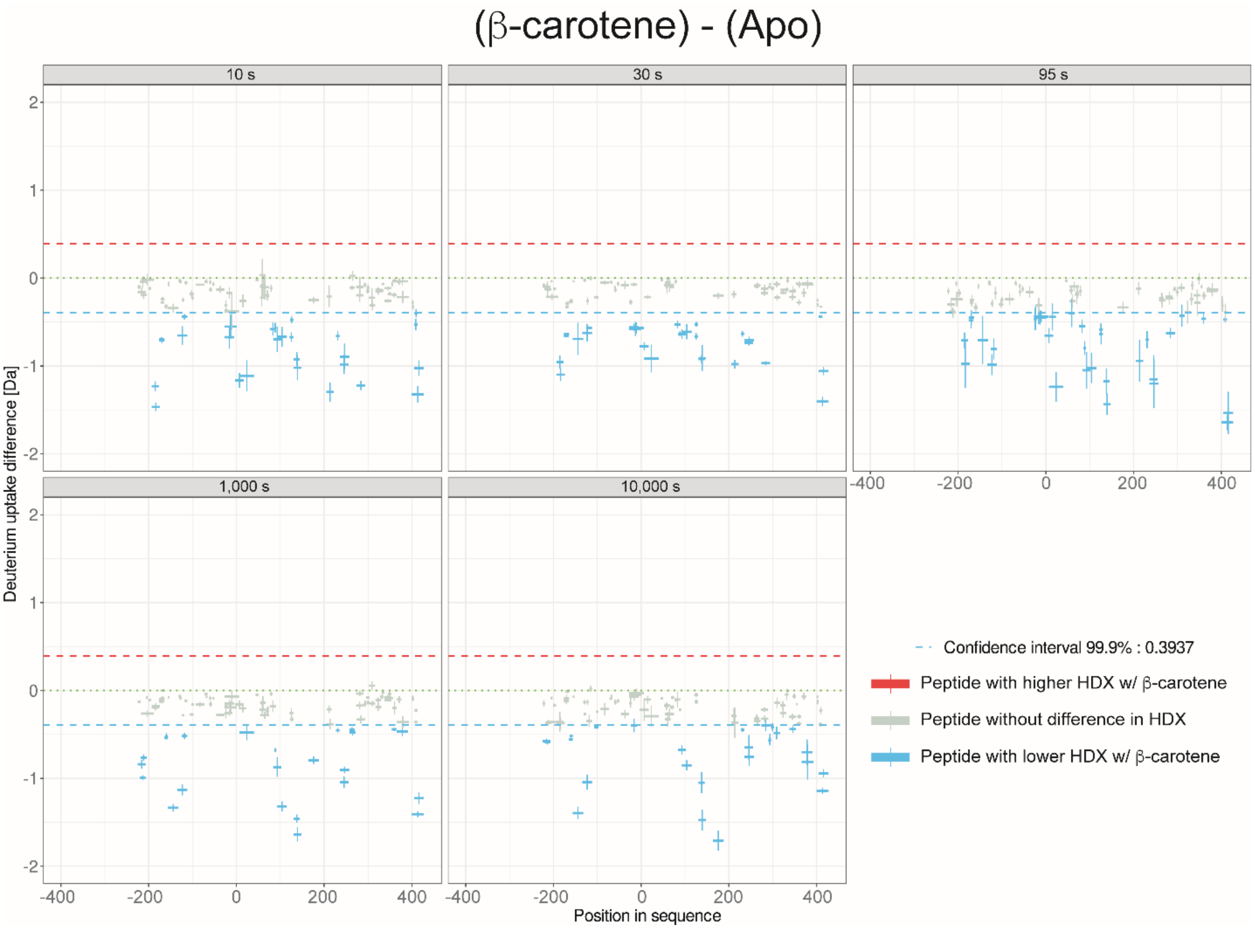

Supplementary Figure 30.

Absolute HDX difference of *Nb*UGT72AY1 between β-carotene and apo state. HDX changes were plotted per peptide (Supplementary Data 6) with HaDeX[5] and slightly modified graphically. Blue and red color denotes peptides with reduced or elevated absolute HDX in presence of β-carotene, respectively, and grey color indicates lack of significant changes.

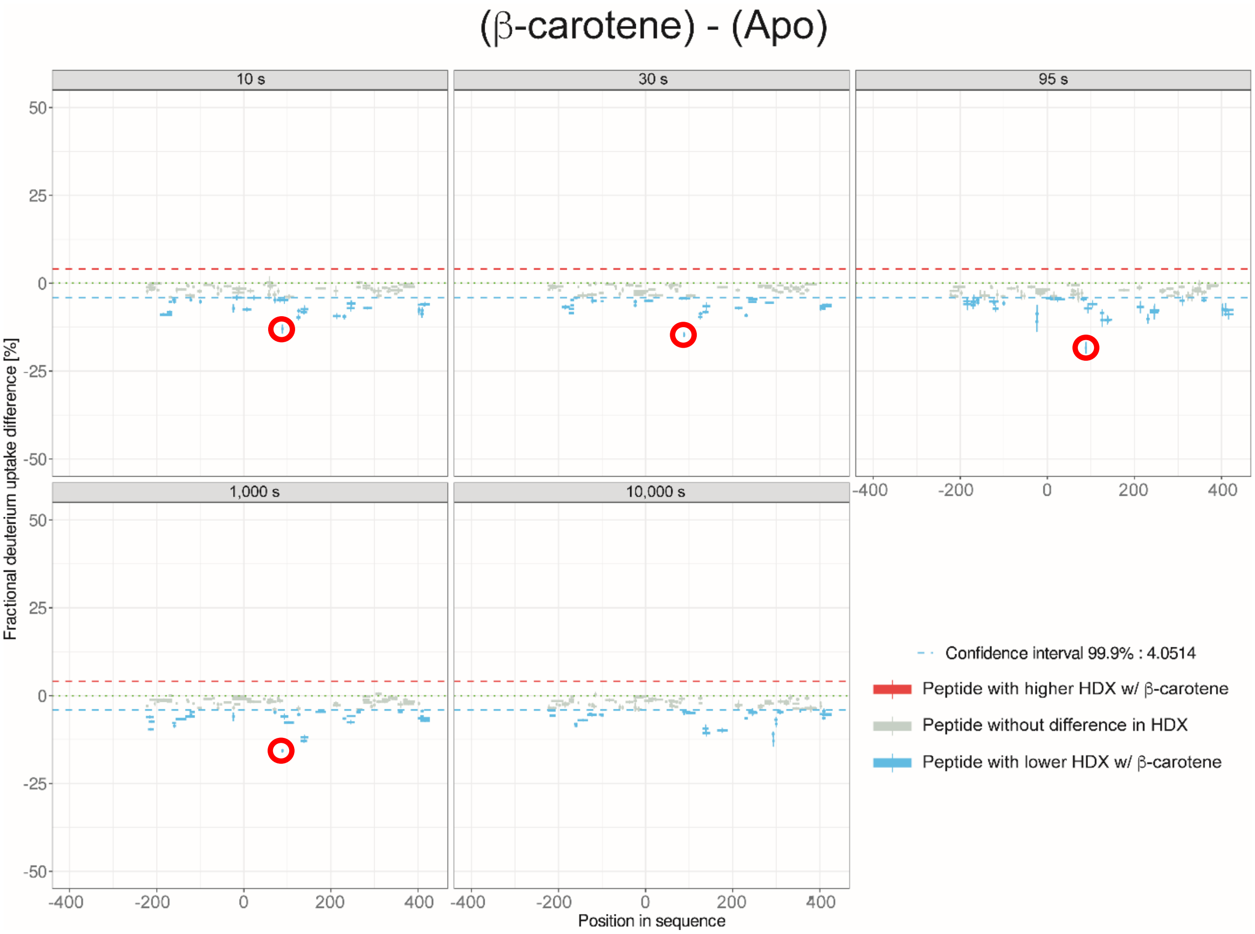

Supplementary Figure 31.

Fractional HDX difference of *Nb*UGT72AY1 between β-carotene and apo state. HDX changes were plotted per peptide (Supplementary Data 6) with HaDeX[5] and slightly modified graphically. Blue and red color denotes peptides with reduced or elevated fractional HDX in presence of β-carotene, respectively, and grey color indicates lack of significant changes. Red circle shows Phe87-Arg91.

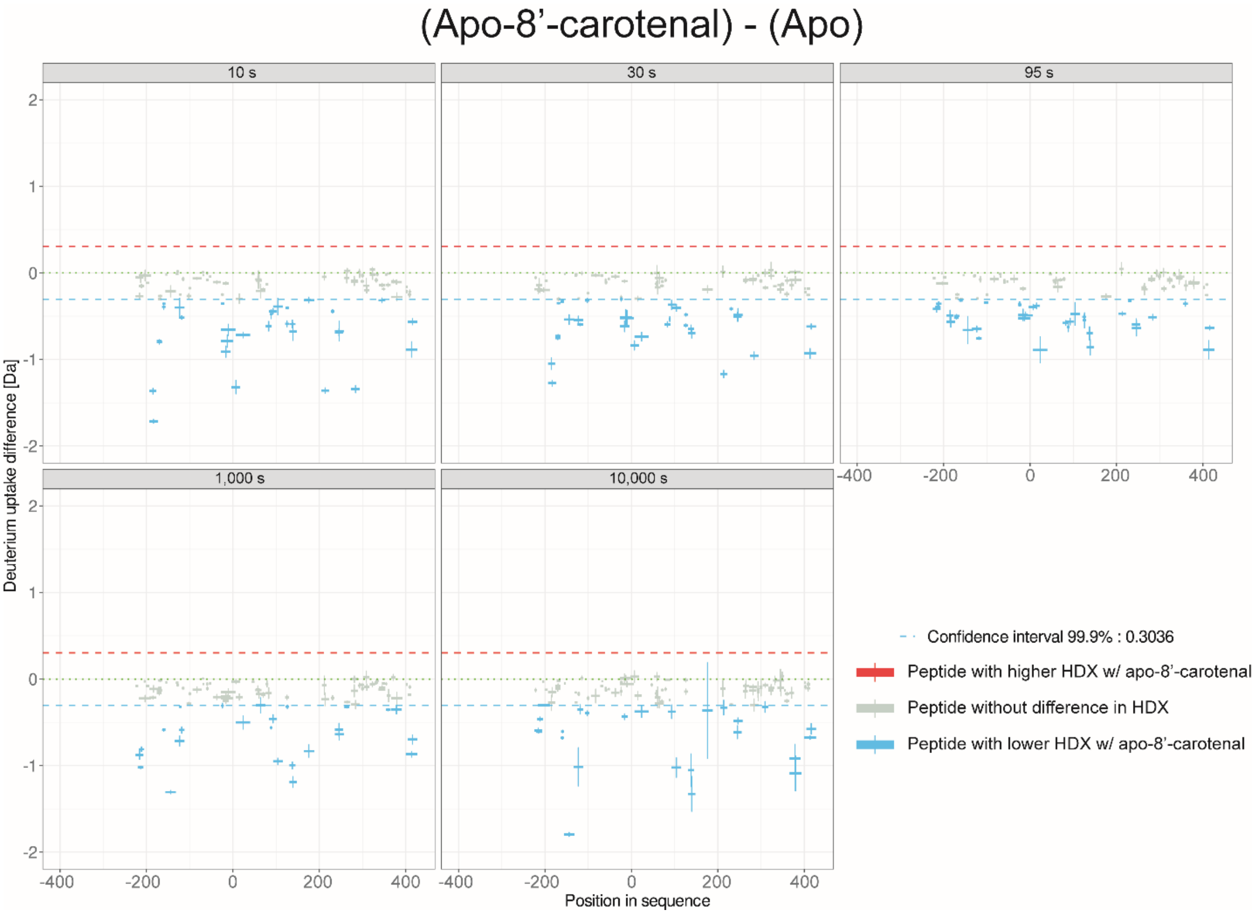

Supplementary Figure 32.

Absolute HDX difference of *Nb*UGT72AY1 between β-apo-8'-carotenal and apo state. HDX changes were plotted per peptide (Supplementary Data 6) with HaDeX[5] and slightly modified graphically. Blue and red color denotes peptides with reduced or elevated absolute HDX in presence of β-apo-8'-carotenal, respectively, and grey color indicates lack of significant changes.

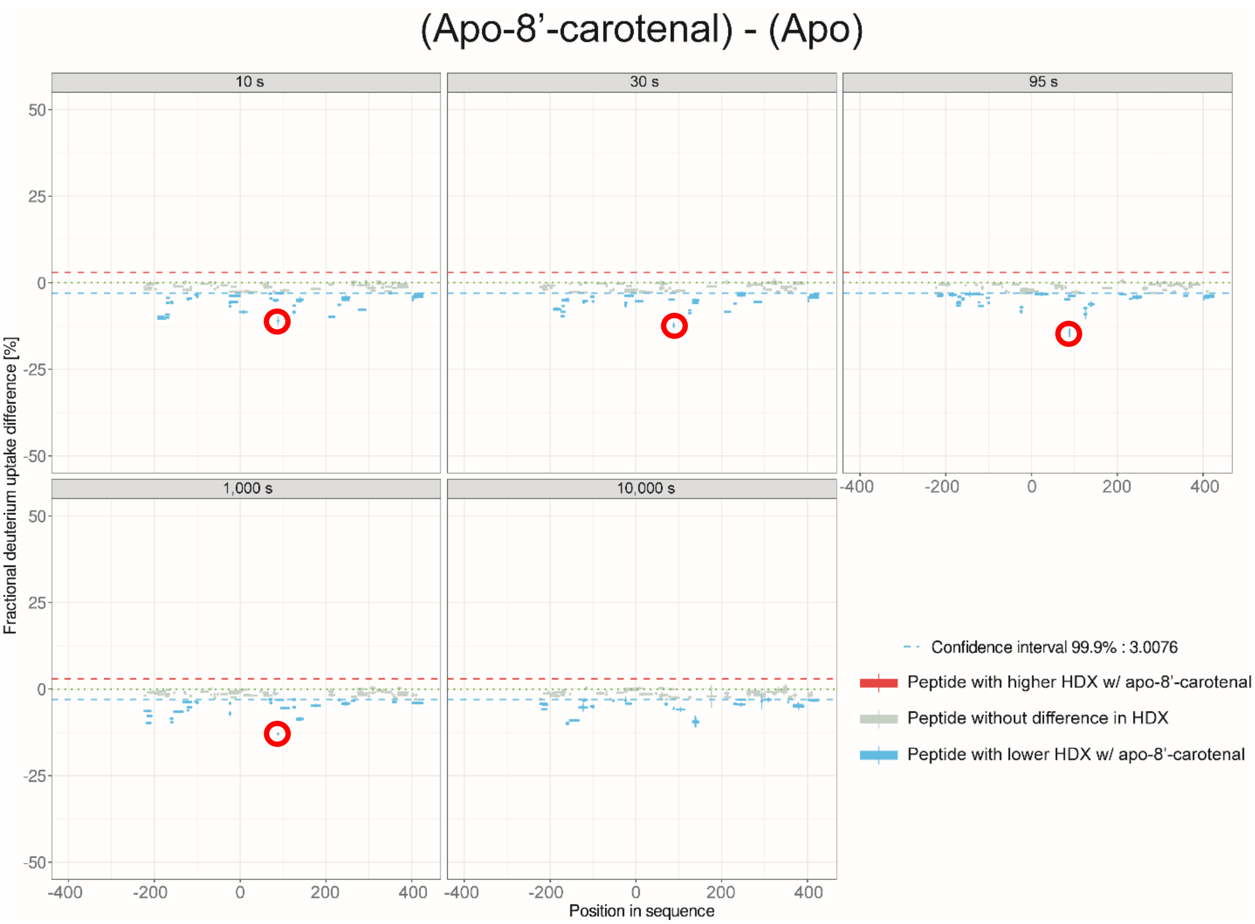

Supplementary Figure 33.

Fractional HDX difference of *Nb*UGT72AY1 between β-apo-8'-carotenal and apo state. HDX changes were plotted per peptide (Supplementary Data 6) with HaDeX[5] and slightly modified graphically. Blue and red color denotes peptides with reduced or elevated fractional HDX in presence of β-apo-8'-carotenal, respectively, and grey color indicates lack of significant changes. Red circle shows Phe87-Arg91.

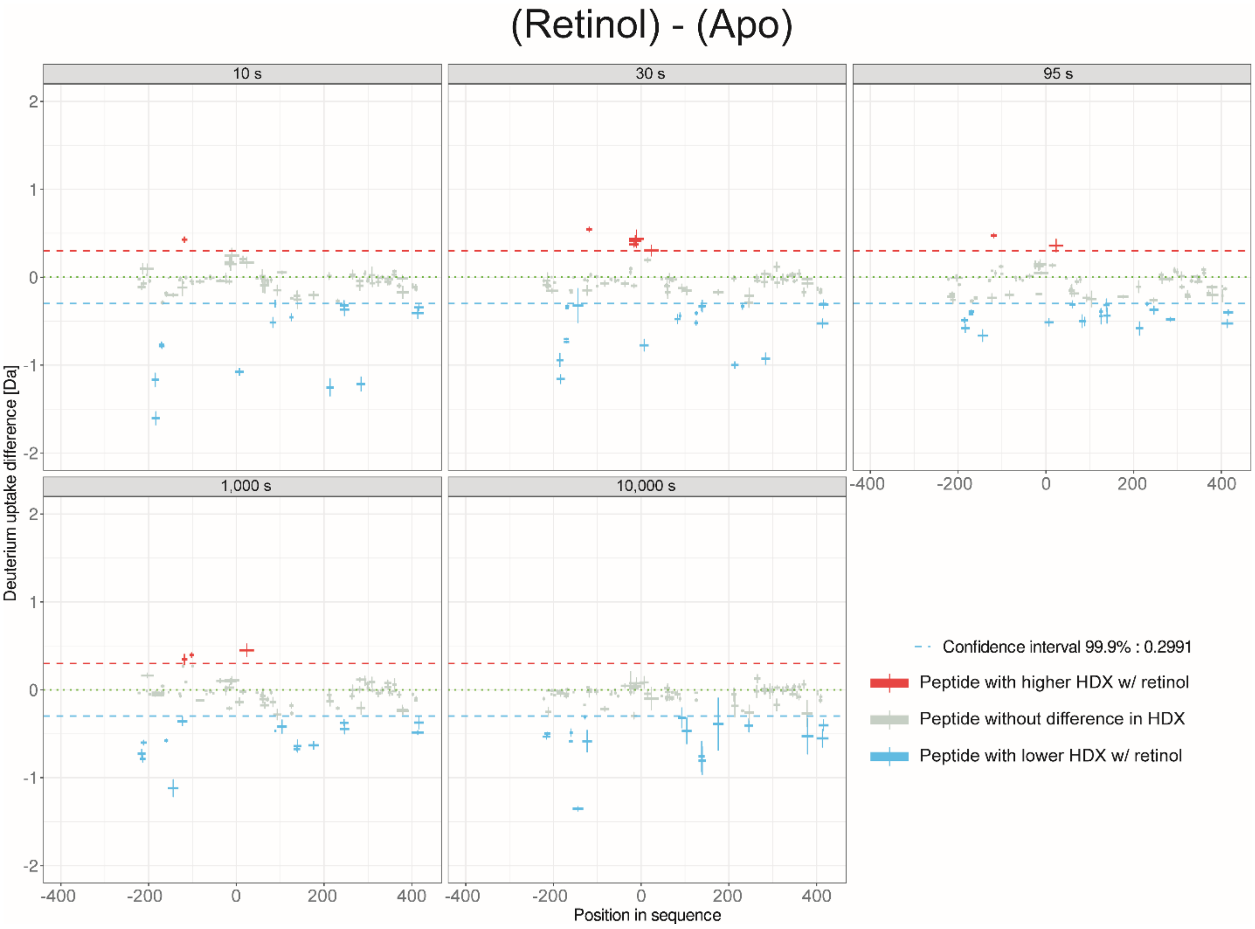

Supplementary Figure 34.

Absolute HDX difference of *Nb*UGT72AY1 between retinol and apo state. HDX changes were plotted per peptide (Supplementary Data 6) with HaDeX[5] and slightly modified graphically. Blue and red color denotes peptides with reduced or elevated absolute HDX in presence of retinol, respectively, and grey color indicates lack of significant changes.

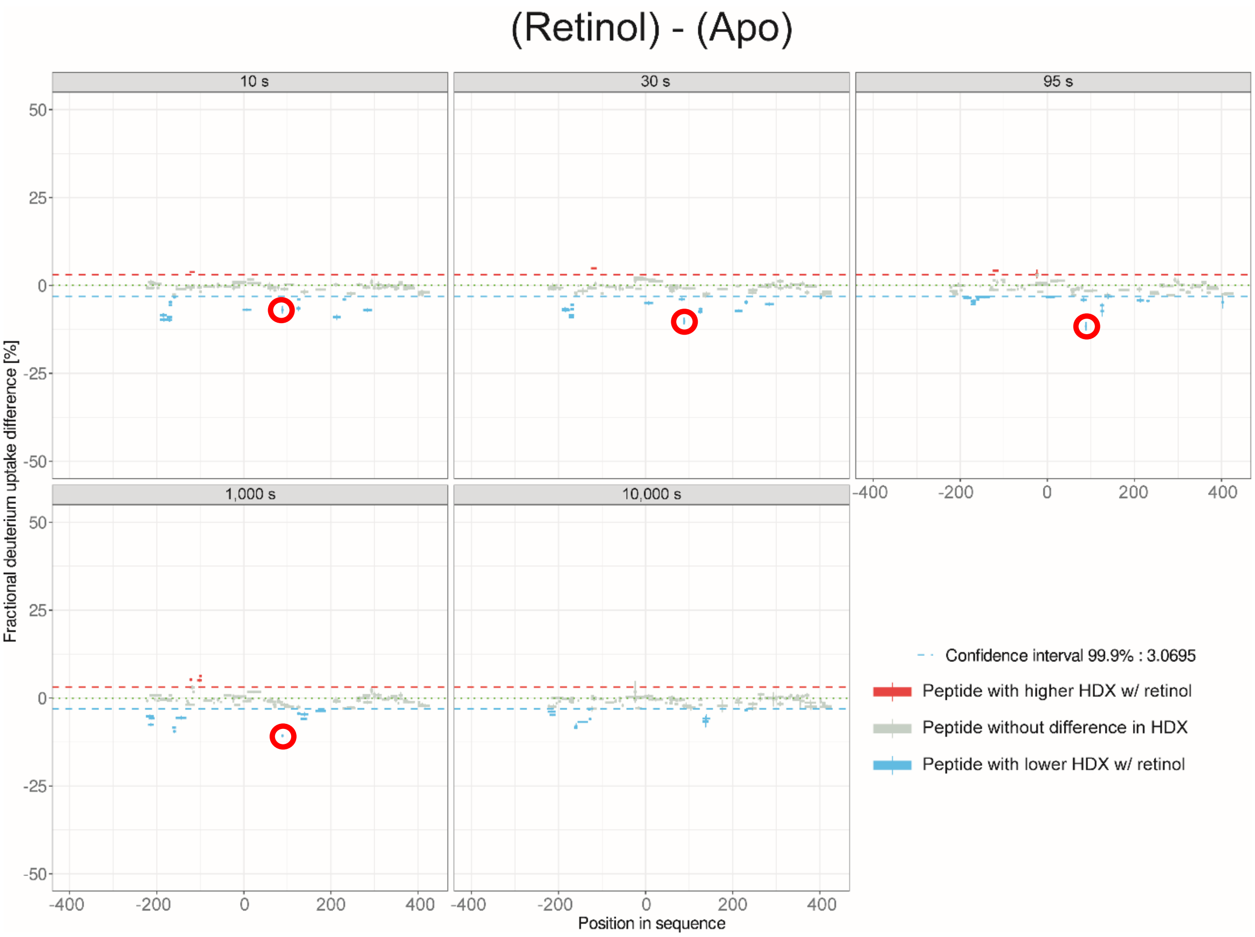

Supplementary Figure 35.

Fractional HDX difference of *Nb*UGT72AY1 between retinol and apo state. HDX changes were plotted per peptide (Supplementary Data 6) with HaDeX[5] and slightly modified graphically. Blue and red color denotes peptides with reduced or elevated fractional HDX presence of retinol, respectively, and grey color indicates lack of significant changes. Red circle shows Phe87-Arg91.

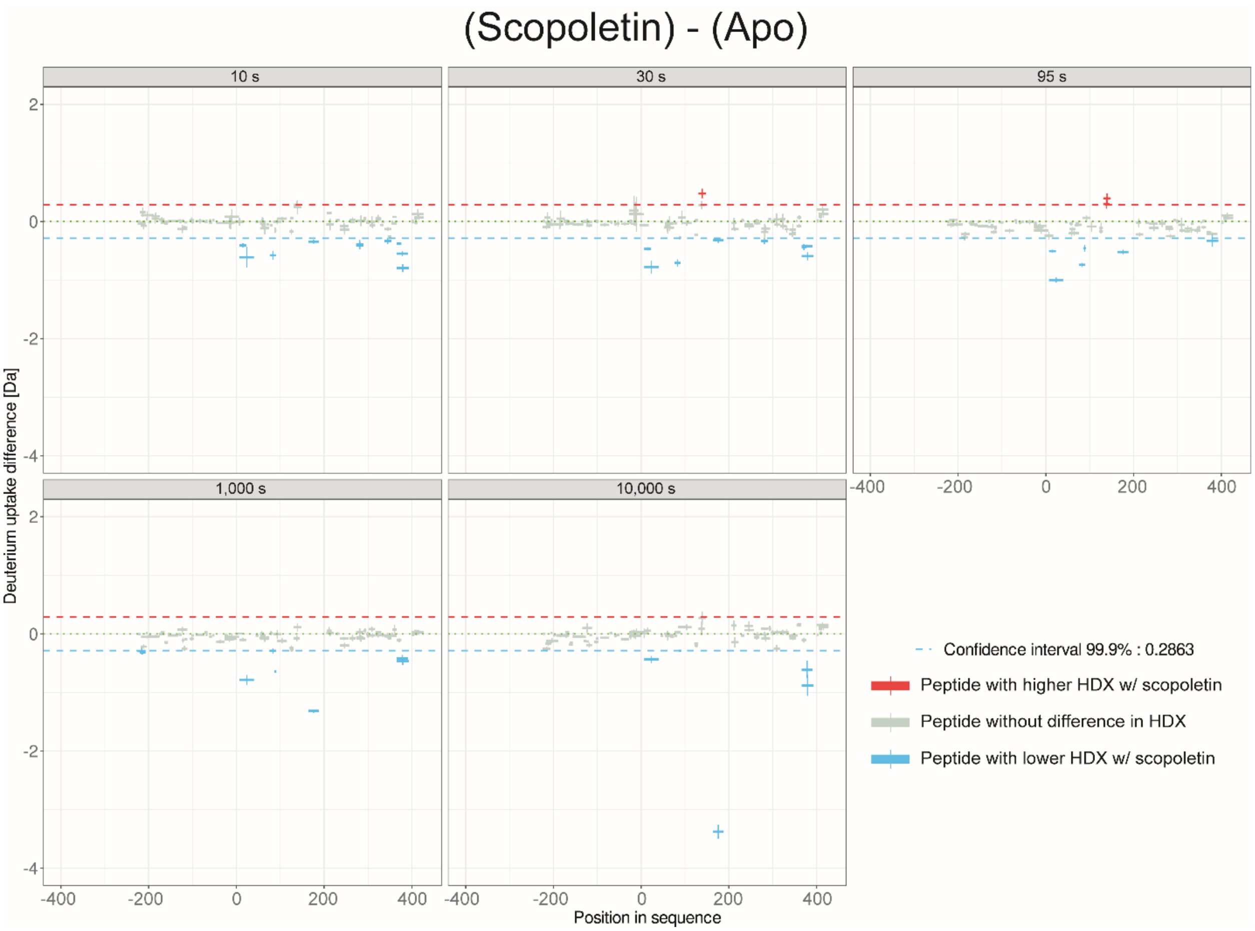

Supplementary Figure 36.

Absolute HDX difference of *Nb*UGT72AY1 between scopoletin and apo state. HDX changes were plotted per peptide (Supplementary Data 6) with HaDeX[5] and slightly modified graphically. Blue and red color denotes peptides with reduced or elevated absolute HDX in presence of scopoletin, respectively, and grey color indicates lack of significant changes.

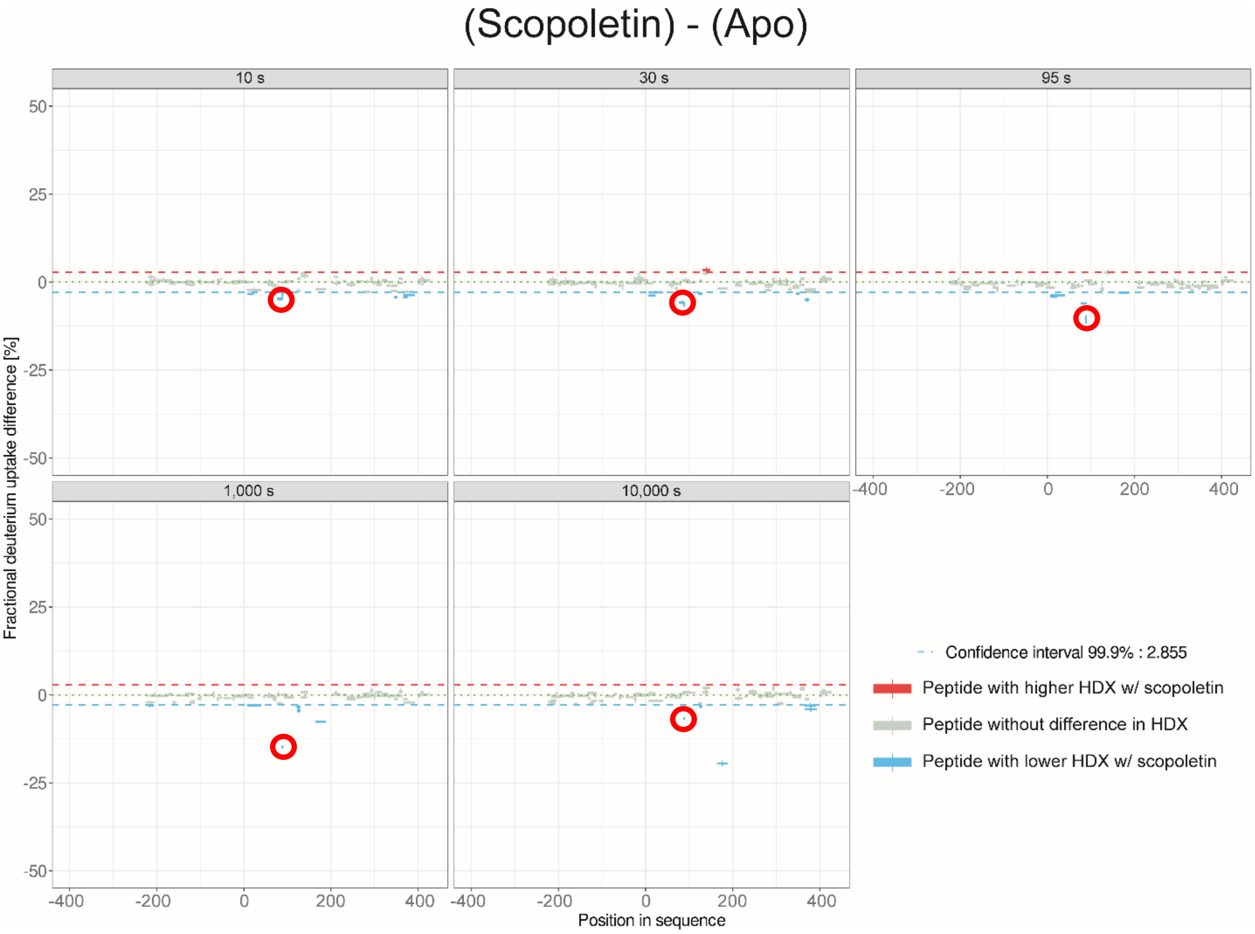

Supplementary Figure 37.

Fractional HDX difference of *Nb*UGT72AY1 between scopoletin and apo state. HDX changes were plotted per peptide (Supplementary Data 6) with HaDeX[5] and slightly modified graphically. Blue and red color denotes peptides with reduced or elevated fractional HDX in presence of scopoletin, respectively, and grey color indicates lack of significant changes. Red circle shows Phe87-Arg91.

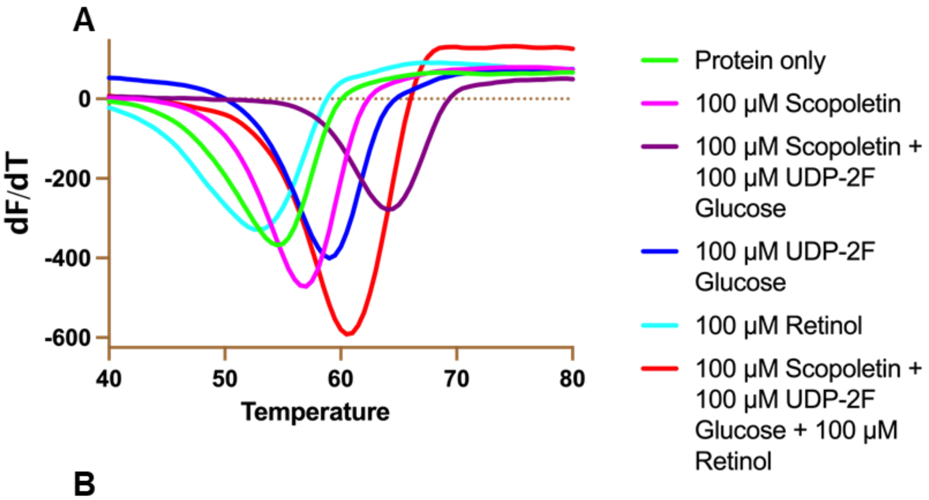

B

Sample	Melting Temperature Tm (℃)
Glucosyl Transferase	55 ± 0.4
Glucosyl Transferase + 100 µM Scopoletin	57 ± 0.3
Glucosyl Transferase + 100 µM UDP-2F Glucose	59.5 ± 0.5
Glucosyl Transferase + 100 µM Retinol	53 ± 0.5
Glucosyl Transferase + 100 µM Scopoletin + 100 µM UDP-2F Glucose	65 ± 0.5
Glucosyl Transferase + 100 µM Scopoletin + 100 µM UDP-2F Glucose + 100 µM Retinol	61 ± 0.3

Supplementary Figure 38.

Differential scanning fluorimetry (DSF). DSF to measure the unfolding of *Nb*UGT72AY1 and in complexes with different ligands as a function of temperature. (**A**) Melting curves. (**B**) Melting temperatures.

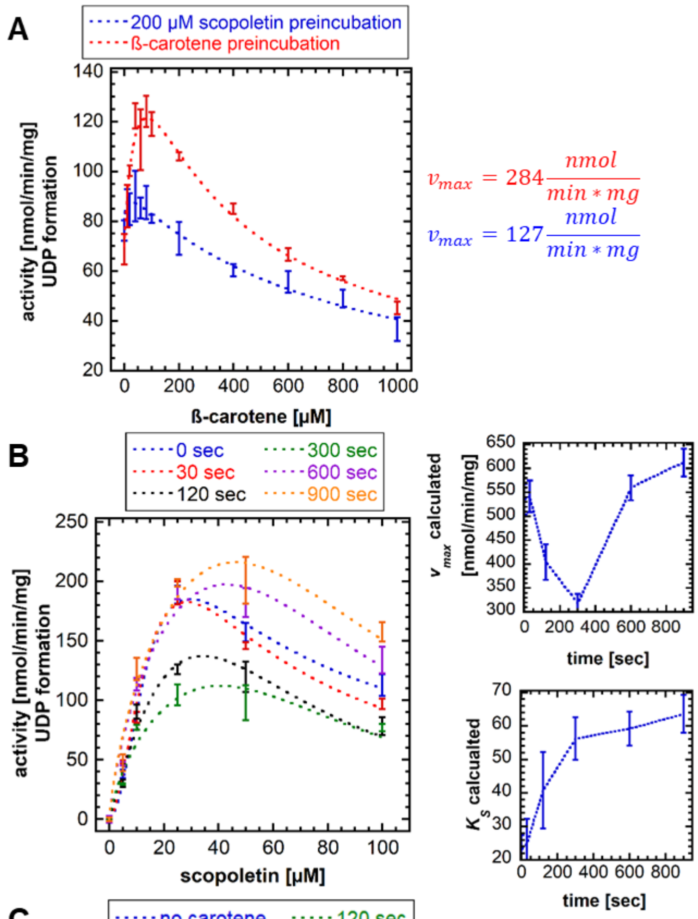

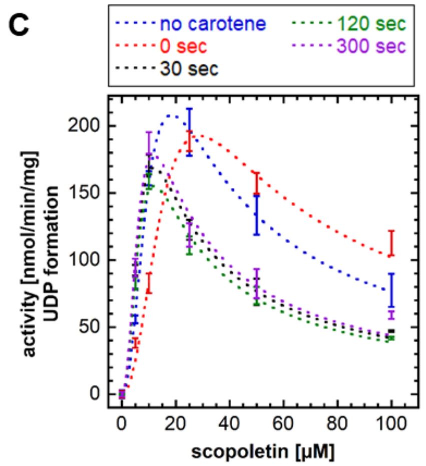

time	v_{max}	± error	K_S	± error	Hill *n*	Hill *x*	R^2	Chi square
no carotene	601	6	15	1	2	3	1.00	41
0 min	556	17	23	1	2	3	0.99	314
30 sec	488	10	9	1	2	3	0.99	107
120 sec	448	12	9	1	2	3	0.99	141
300 sec	514	26	9	1	2	3	0.96	709

Supplementary Figure 39.

***Nb*UGT72AY1 shows hysteresis.** (**A**) The master mix contained either increasing concentrations of β-carotene or 200 µM scopoletin, which were pre-incubated with the enzyme before the reaction was started. (**B**) One hundred µM β-carotene was pre-incubated with the enzyme for the indicated time intervals before starting the reaction with UDPG/scopoletin. (**C**) One hundred µM scopoletin was pre-incubated with the enzyme for the indicated time intervals before starting the reaction with UDPG/β-carotene. Reaction rates were determined using the UDP-Glo™ assay. Equation 2 was used to fit the curves. Source data are provided as a Source Data file. n=5 independent experiments.

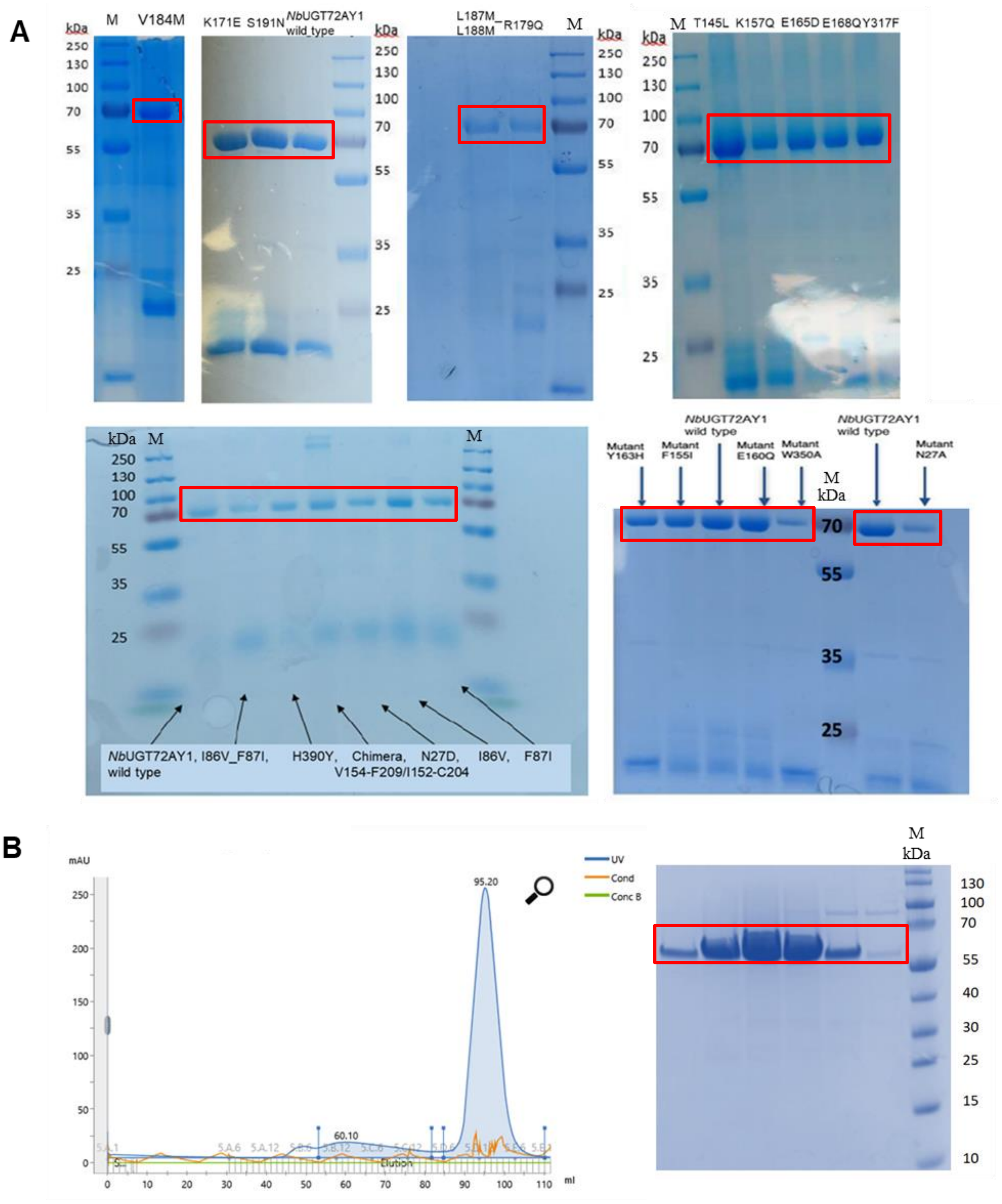

Supplementary Figure 40.

Protein analysis. (**A**) SDS-PAGE analysis of recombinant *Nb*UGT72AY1 wild type and mutants. (**B**) Size exclusion peak of the GST cleaved *Nb*UGT72AY1 and the SDS-PAGE gel of the fractions under the peak. M, maker proteins. *Nb*UGT72A1 and its mutants are outlined in red.

Supplementary Table.

Summary Table. MD simulations

**Reliability and reproducibility checklist for molecular dynamics simulations**<br>***All boxes must be marked YES by acceptance unless "Response not needed if No".**	**Yes**	**No**	**Response (Please state where this information can be found in the text)**
**1. Convergence of simulations and analysis**			
1a. Is an evaluation presented in the text to show that the property being measured has equilibrated in the simulations. (*e.g.* time-course analysis)?	☒	☐	Supplementary Figures 22-25<br>The structure was equilibrated by 4 ns of restrained MD simulations. In the RMSD analysis during production, the systems reach a plateau after a few nanoseconds.
1b. Then, is it described in the text how simulations are split into equilibration and production runs and how much data were analyzed from production runs?	☒	☐	Methods, Molecular Dynamics (MD) simulations.
1c. Are there at least 3 simulations per simulation condition with statistical analysis?	☒	☐	Methods, Molecular Dynamics (MD) simulations; Supplementary Figures 22-26.
1d. Is evidence provided in the text that the simulation results presented are independent of initial configuration?	☒	☐	Methods, Molecular Dynamics (MD) simulations – velocities after equilibration were randomized for each replicate.
**2. Connection to experiments**			
2a. Are calculations provided that can connect to experiments (*e.g.* loss or gain in function from mutagenesis, binding assays, NMR chemical shifts, J-couplings, SAXS curves, interaction distances or FRET distances, structure factors, diffusion coefficients, bulk modulus and other mechanical properties, *etc*.)?	☐	☒	
**3. Method choice**			
3a. Do simulations contain membranes, membrane proteins, intrinsically disordered proteins, glycans, nucleic acids, polymers, or cryptic ligand binding?	☐	☒	
3b. Is it described in the text whether the accuracy of the chosen model(s) is	☒	☐	We perform standard all-atom MD simulations with explicit solvent

sufficient to address the question(s) under investigation (e.g. all-atom vs. coarse-grained models, fixed charge vs. polarizable force fields, implicit vs. explicit solvent or membrane, force field and water model, etc.)?			(TIP3P) using the AMBER force field, which is the most commonly used for globular proteins (Supplementary Figures 25-26.
3c. Is the timescale of the event(s) under investigation beyond the brute-force MD simulation timescale in this study that enhanced sampling methods are needed?	☐	☒	
If **YES**, are the parameters and convergence criteria for the enhanced sampling method clearly stated?	☐	☐	
If **NO,** is the evidence provided in the text?	☒	☐	Supplementary Figure 26.
**4. Code and reproducibility**			
4a. Is a table provided describing the system setup that includes simulation box dimensions, total number of atoms, total number of water molecules, salt concentration, lipid composition (number of molecules and type)?	☒	☐	Supplementary Data 5
4b. Is it described in the text what simulation and analysis software and which versions are used?	☒	☐	Methods, Molecular Dynamics (MD) simulations
4c. Are other parameters for the system setup described in the text, such as protonation state, type of structural restraints if applied, nonbonded cutoff, thermostat and barostat, etc.?	☒	☐	Methods, Molecular Dynamics (MD) simulations
4d. Are initial coordinate and simulation input files and a coordinate file of the final output provided as supplementary files or in a public repository?	☒	☐	Deposited in https://zenodo.org/records/14001338
4e. Is there custom code or custom force field parameters?	☐	☒	
If **YES**, are they provided as supplementary files or in a public repository?	☐	☐	

Supplementary References

1. Eisenberg, A. S. & Juszczak, L. J. Correlation of TrpGly and GlyTrp Rotamer Structure with W7 and W10 UV Resonance Raman Modes and Fluorescence Emission Shifts. *J. Amino Acids* **2012,** 735076; 10.1155/2012/735076 (2012).

2. Houde, D., Berkowitz, S. A. & Engen, J. R. The utility of hydrogen/deuterium exchange mass spectrometry in biopharmaceutical comparability studies. *J. Pharm. Sci.* **100,** 2071–2086; 10.1002/jps.22432 (2011).

3. Liao, J. *et al.* Subfunctionalization of a monolignol to a phytoalexin glucosyltransferase is accompanied by substrate inhibition. *Plant Commun.* **4,** 100506; 10.1016/j.xplc.2022.100506 (2023).

4. Sun, G. *et al.* Apocarotenoids are allosteric effectors of a dimeric plant glycosyltransferase involved in defense and lignin formation. *New Phytol.* **238,** 2080–2098; 10.1111/nph.18875 (2023).

5. Puchała, W. *et al.* HaDeX: an R package and web-server for analysis of data from hydrogen-deuterium exchange mass spectrometry experiments. *Bioinformatics* **36,** 4516–4518; 10.1093/bioinformatics/btaa587 (2020).

6. Adkar, B. V., Bhattacharyya, S., Gilson, A. I., Zhang, W. & Shakhnovich, E. I. Substrate inhibition imposes fitness penalty at high protein stability. *Proc. Natl. Acad. Sci. U. S. A.* **116,** 11265–11274; 10.1073/pnas.1821447116 (2019).

7. LiCata, V. J. & Allewell, N. M. Is substrate inhibition a consequence of allostery in aspartate transcarbamylase? *Biophys. Chem.* **64,** 225–234; 10.1016/S0301-4622(96)02204-1 (1997).

8. Wang, J., Stieglitz, K. A., Cardia, J. P. & Kantrowitz, E. R. Structural basis for ordered substrate binding and cooperativity in aspartate transcarbamoylase. *Proc. Natl. Acad. Sci. U. S. A.* **102,** 8881–8886; 10.1073/pnas.0503742102 (2005).

9. Peng, H., Yang, T., Whitaker, B. D., Shangguan, L. & Fang, J. Calcium/calmodulin alleviates substrate inhibition in a strawberry UDP-glucosyltransferase involved in fruit anthocyanin biosynthesis. *BMC Plant Biol.* **16,** 197; 10.1186/s12870-016-0888-z (2016).

10. Krasauskas, R. *et al.* Purification and characterization of a new β-lactamase OXA-205 from Pseudomonas aeruginosa. *Ann. Clin. Microbiol. Antimicrob.* **14,** 52; 10.1186/s12941-015-0113-1 (2015).

11. Larion, M. & Miller, B. G. Homotropic allosteric regulation in monomeric mammalian glucokinase. *Arch. Biochem. Biophys.* **519,** 103–111; 10.1016/j.abb.2011.11.007 (2012).

12. Porter, C. M. & Miller, B. G. Cooperativity in monomeric enzymes with single ligand-binding sites. *Bioorg. Chem.* **43,** 44–50; 10.1016/j.bioorg.2011.11.001 (2012).

13. Sun, G. *et al.* Glucosylation of the phytoalexin N-feruloyl tyramine modulates the levels of pathogen-responsive metabolites in Nicotiana benthamiana. *Plant J.* **100,** 20–37; 10.1111/tpj.14420 (2019).

14. Del Conte, A. *et al.* RING 4.0: faster residue interaction networks with novel interaction types across over 35,000 different chemical structures. *Nucleic Acids Res.* **52,** W306-W312; 10.1093/nar/gkae337 (2024).

15. Tsai, C.-J. & Nussinov, R. A unified view of "how allostery works". *PLoS Comput. Biol.* **10,** e1003394; 10.1371/journal.pcbi.1003394 (2014).

Uncropped Scans from gels in Supplementary Figure 40.

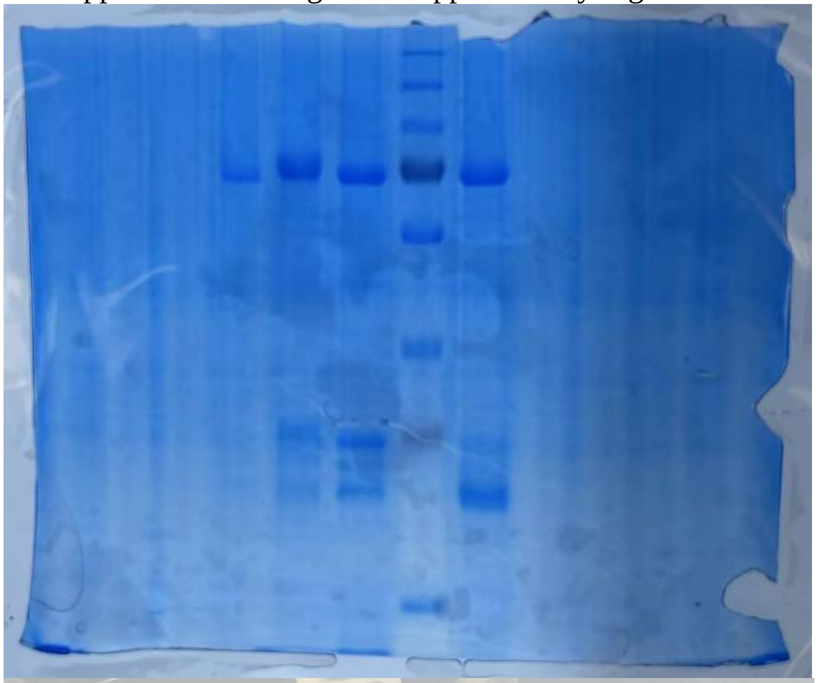

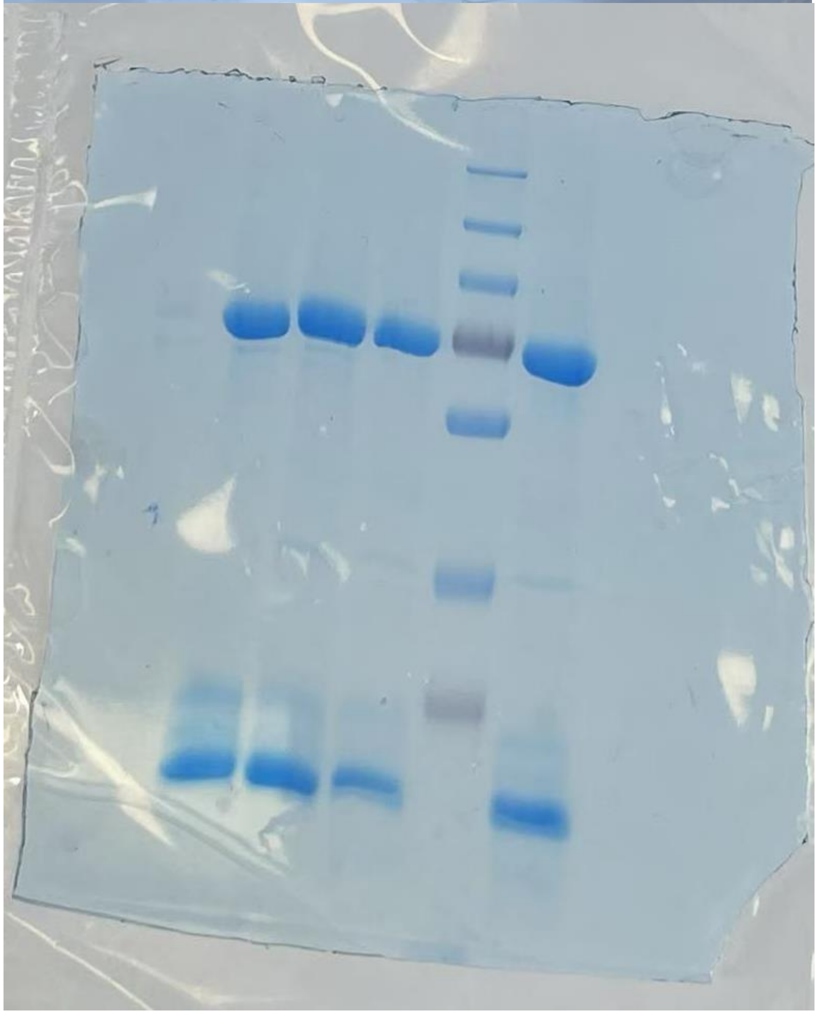

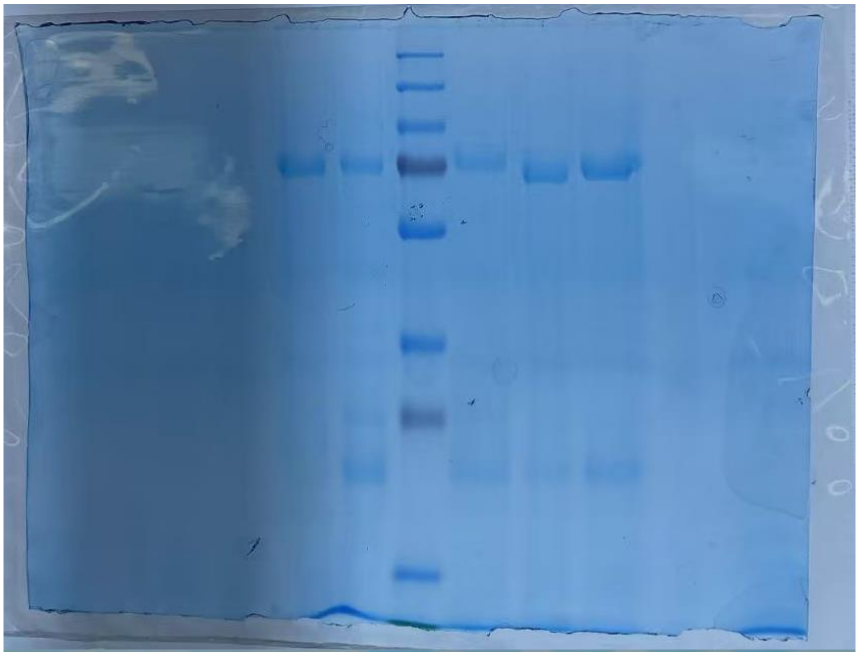

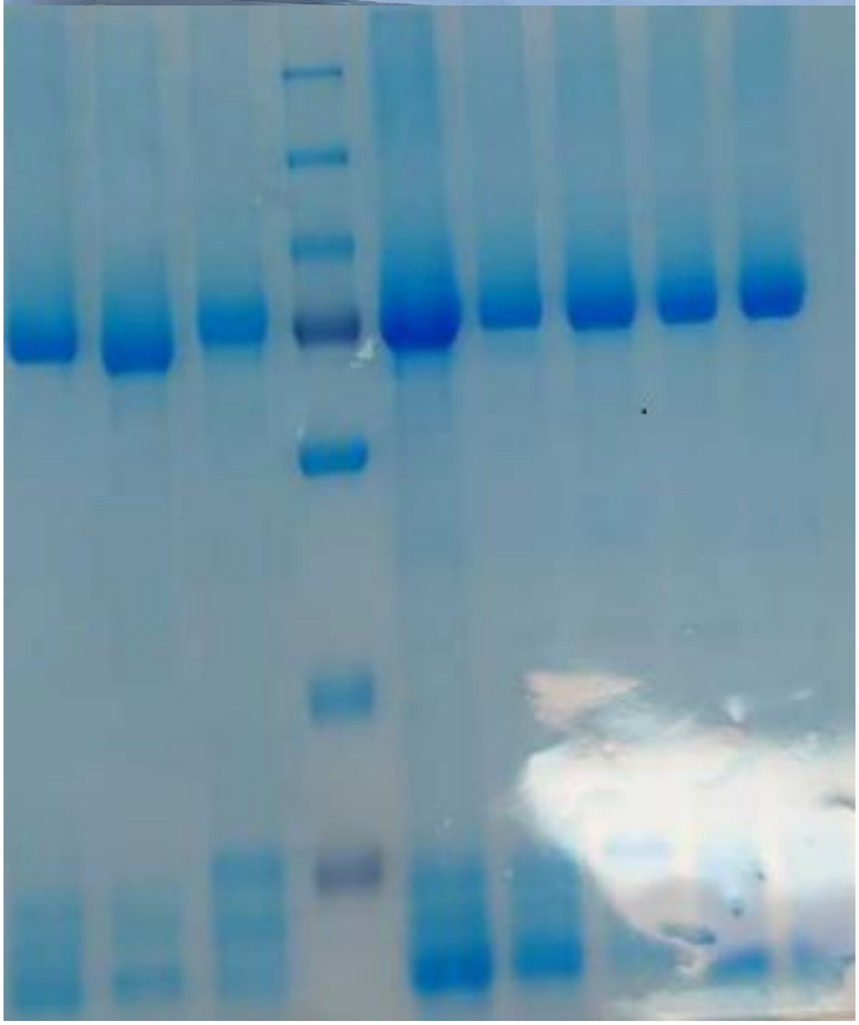

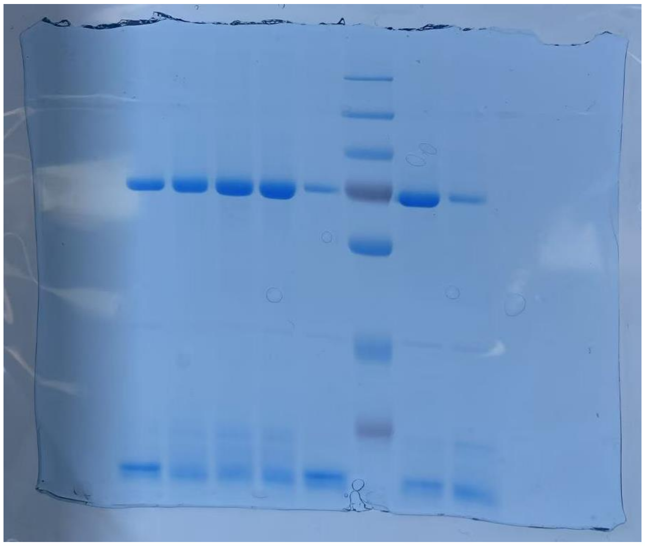

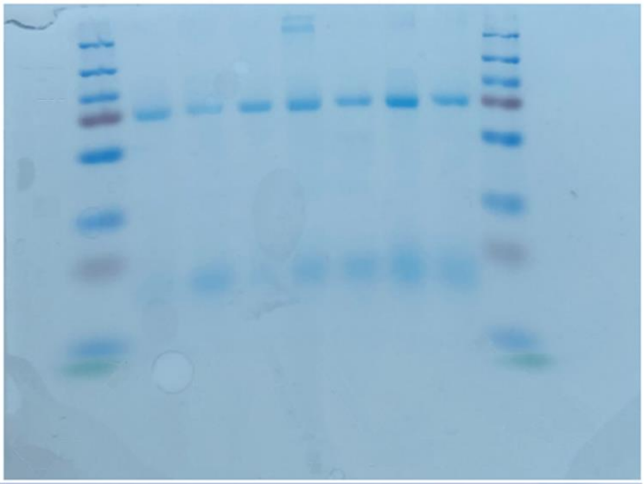

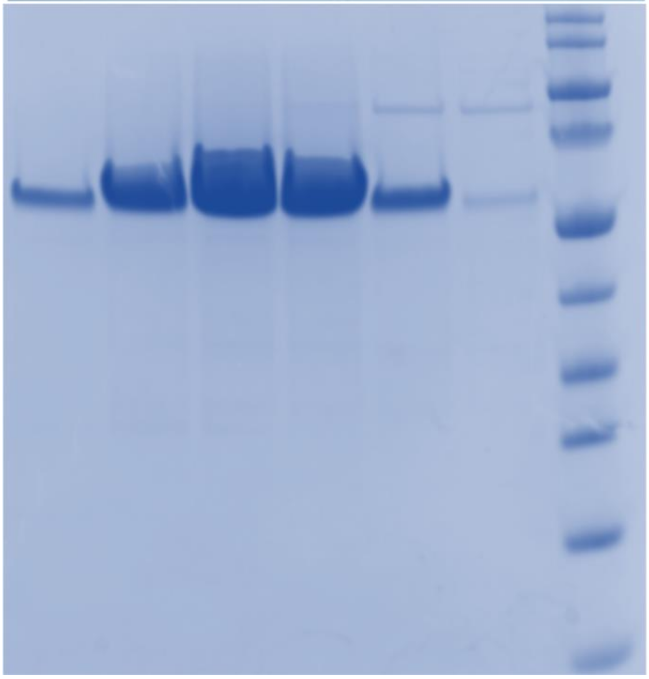

Supplement: Supplementary file 1 — Supplementary Information [file 41467_2025_58259_MOESM1_ESM.pdf]
