# Supplementary material for: β-Carotene alleviates substrate inhibition caused by asymmetric cooperativity
Source: Nat Commun. 2025 Mar 29;16:3065. doi: 10.1038/s41467-025-58259-7 (PMC11954892; doi:10.1038/s41467-025-58259-7)
Supplement: Supplementary file 2 — Description of Additional Supplementary Files [file 41467_2025_58259_MOESM2_ESM.docx]

**Description of Additional Supplementary Files**

Supplementary Data 1. PDB codes and crystallization conditions.

Supplementary Data 2. Summary of crystal parameters

Supplementary Data 3. RMSD values.

Supplementary Data 4. Kinetic parameters.

Supplementary Data 5. List and information of simulated systems.

Supplementary Data 6. Overview of data obtained by hydrogen/deuterium exchange mass spectrometry (HDX-MS).
