# Supplementary material for: β-Carotene alleviates substrate inhibition caused by asymmetric cooperativity
Source: Nat Commun. 2025 Mar 29;16:3065. doi: 10.1038/s41467-025-58259-7 (PMC11954892; doi:10.1038/s41467-025-58259-7)
Supplement: Supplementary file 9 — Reporting Summary [file 41467_2025_58259_MOESM9_ESM.pdf]

# nature portfolio

Corresponding author(s): Wilfried Schwab

Last updated by author(s): Feb 21, 2025

# Reporting Summary

Nature Portfolio wishes to improve the reproducibility of the work that we publish. This form provides structure for consistency and transparency in reporting. For further information on Nature Portfolio policies, see our Editorial Policies and the Editorial Policy Checklist.

## Statistics

For all statistical analyses, confirm that the following items are present in the figure legend, table legend, main text, or Methods section.

| n/a | Confirmed | |
|---|---|---|
| ☐ | ☒ | The exact sample size (*n*) for each experimental group/condition, given as a discrete number and unit of measurement |
| ☒ | ☐ | A statement on whether measurements were taken from distinct samples or whether the same sample was measured repeatedly |
| ☐ | ☒ | The statistical test(s) used AND whether they are one- or two-sided<br>*Only common tests should be described solely by name; describe more complex techniques in the Methods section.* |
| ☒ | ☐ | A description of all covariates tested |
| ☒ | ☐ | A description of any assumptions or corrections, such as tests of normality and adjustment for multiple comparisons |
| ☐ | ☒ | A full description of the statistical parameters including central tendency (e.g. means) or other basic estimates (e.g. regression coefficient) AND variation (e.g. standard deviation) or associated estimates of uncertainty (e.g. confidence intervals) |
| ☐ | ☒ | For null hypothesis testing, the test statistic (e.g. *F*, *t*, *r*) with confidence intervals, effect sizes, degrees of freedom and *P* value noted<br>*Give P values as exact values whenever suitable.* |
| ☒ | ☐ | For Bayesian analysis, information on the choice of priors and Markov chain Monte Carlo settings |
| ☒ | ☐ | For hierarchical and complex designs, identification of the appropriate level for tests and full reporting of outcomes |
| ☒ | ☐ | Estimates of effect sizes (e.g. Cohen's *d*, Pearson's *r*), indicating how they were calculated |

*Our web collection on statistics for biologists contains articles on many of the points above.*

## Software and code

Policy information about availability of computer code

| | |
|---|---|
| Data collection | CLARIOstar plate reader (BMG Labtech, Offenburg, Germany), all data were collected at 100 K at the beamlines Proxima 1 and Proxima 2A at the SOLEIL Synchrotron (France), using a EIGER-X 16M and EIGER-X 9M detectors, respectively (proposal numbers 2021095, 20210932, 20220373, and 20221065), the data were processed in XDS, assay was performed on CFX96 Real-Time System, Graphpad 9.0 (Prism; www.graphpad.com), Agilent HPLC following DAWN MALS detector (Wyatt Technology). Data were analysed using ASTRA software provided by the company, Prime (Schrödinger Release 2022-1, Prime, Schrödinger, LLC, New York, NY, 2022), Protein Preparation Wizard tool (Schrödinger Release 2022-1, Maestro, Schrödinger, LLC, New York, NY, 2022), TIP3P water model, Leap software implemented in AMBER (The Amber Molecular Dynamics Package, at http://ambermd.org) and AmberTools22. The protein was parametrized using the ff14SB force field 50. Antechamber and parmcheck2 tools, assigning parameters from GAFF force field 51. ACEMD 52 (Acellera, version 3.7) was used for the MD simulations, Langevin thermostat, using a Montecarlo barostat. M-SHAKE algorithm, particle mesh Ewald summation method, in-house Python script based on MDAnalysis (v2.2.0), MDtraj (v1.9.7), Matplotlib Python library, data processing by DynamX 3.0, MicroCal PEAQ-ITC, Chirascan Spectropolarimeter, BestSel for CD data evaluation, CFX96 Real-Time System (BIO-RAD, Hercules, CA, USA) thermal cycler was used for determination of melting temperatures; Chirascan V100 from Applied Photophysics. |
| Data analysis | Excel 2016, KaleidaGraph 4.5.4, Chimera 1.17.1, CLARIOStar Software Package (Version 5.21 R2, Firmware Version: 1.15, MARS: 3.20 R2), Chirascan Software Suit (Pro-Data Viewer) |

For manuscripts utilizing custom algorithms or software that are central to the research but not yet described in published literature, software must be made available to editors and reviewers. We strongly encourage code deposition in a community repository (e.g. GitHub). See the Nature Portfolio guidelines for submitting code & software for further information.

## Data

Policy information about availability of data

All manuscripts must include a data availability statement. This statement should provide the following information, where applicable:

- Accession codes, unique identifiers, or web links for publicly available datasets
- A description of any restrictions on data availability
- For clinical datasets or third party data, please ensure that the statement adheres to our policy

Accession code for NbUGT72AY1 at GenBank UHH90560.1 (protein), MT945401.1 (mRNA) and StUGT72AY2 at Genbank XP_015164078.1 (protein), XM_015308592.1 (mRNA). All X-ray crystal structures generated in this study are deposited at pdb (https://www.rcsb.org/structure/; provisional IDs: 8J2Z, 8J31, 8J2V, 8J2U, 9J9K, 9LRJ). MD trajectories and related files (topology, parameter, and coordinates) are available at Zenodo (https://zenodo.org/records/14001338). The mass spectrometry proteomics data have been deposited to the ProteomeXchange Consortium via the PRIDE78 partner repository with the dataset identifier PXD056344. Source data are provided with this paper.

## Research involving human participants, their data, or biological material

Policy information about studies with human participants or human data. See also policy information about sex, gender (identity/presentation), and sexual orientation and race, ethnicity and racism.

| | |
|---|---|
| Reporting on sex and gender | n/a |
| Reporting on race, ethnicity, or other socially relevant groupings | n/a |
| Population characteristics | n/a |
| Recruitment | n/a |
| Ethics oversight | n/a |

Note that full information on the approval of the study protocol must also be provided in the manuscript.

# Field-specific reporting

Please select the one below that is the best fit for your research. If you are not sure, read the appropriate sections before making your selection.

[x] Life sciences  [ ] Behavioural & social sciences  [ ] Ecological, evolutionary & environmental sciences

For a reference copy of the document with all sections, see nature.com/documents/nr-reporting-summary-flat.pdf

# Life sciences study design

All studies must disclose on these points even when the disclosure is negative.

| | |
|---|---|
| Sample size | The sample size is indicated in the respective legends. |
| Data exclusions | Data was not excluded |
| Replication | The enzyme studies ran for 5 years, the tests were carried out by more than ten independent persons, the wild-type enzyme was therefore analyzed more than 50 times and served as a control for the mutant experiments, which were repeated at least twice with 5 repetitions each, HDX experiments were carried out three times within 5 years. In addition to the biological replicates, at least three technical replicates were carried out. |
| Randomization | n/a |
| Blinding | n/a |

# Reporting for specific materials, systems and methods

We require information from authors about some types of materials, experimental systems and methods used in many studies. Here, indicate whether each material, system or method listed is relevant to your study. If you are not sure if a list item applies to your research, read the appropriate section before selecting a response.

## Materials & experimental systems

| n/a | Involved in the study |
| --- | --- |
| ☒ | ☐ Antibodies |
| ☒ | ☐ Eukaryotic cell lines |
| ☒ | ☐ Palaeontology and archaeology |
| ☒ | ☐ Animals and other organisms |
| ☒ | ☐ Clinical data |
| ☒ | ☐ Dual use research of concern |
| ☐ | ☒ Plants |

## Methods

| n/a | Involved in the study |
| --- | --- |
| ☒ | ☐ ChIP-seq |
| ☒ | ☐ Flow cytometry |
| ☒ | ☐ MRI-based neuroimaging |

# Dual use research of concern

Policy information about dual use research of concern

## Hazards

Could the accidental, deliberate or reckless misuse of agents or technologies generated in the work, or the application of information presented in the manuscript, pose a threat to:

| No | Yes |
| --- | --- |
| ☒ | ☐ Public health |
| ☒ | ☐ National security |
| ☒ | ☐ Crops and/or livestock |
| ☒ | ☐ Ecosystems |
| ☒ | ☐ Any other significant area |

## Experiments of concern

Does the work involve any of these experiments of concern:

| No | Yes |
| --- | --- |
| ☒ | ☐ Demonstrate how to render a vaccine ineffective |
| ☒ | ☐ Confer resistance to therapeutically useful antibiotics or antiviral agents |
| ☒ | ☐ Enhance the virulence of a pathogen or render a nonpathogen virulent |
| ☒ | ☐ Increase transmissibility of a pathogen |
| ☒ | ☐ Alter the host range of a pathogen |
| ☒ | ☐ Enable evasion of diagnostic/detection modalities |
| ☒ | ☐ Enable the weaponization of a biological agent or toxin |
| ☒ | ☐ Any other potentially harmful combination of experiments and agents |

# Plants

| | |
| --- | --- |
| Seed stocks | Juan C. Moreno and Salim Al-Babili for providing the transgenic DcLCYB1-expressing tobacco plants |
| Novel plant genotypes | n/a |
| Authentication | n/a |
